# Robust machine−learning based prognostic index using cytotoxic T lymphocyte evasion genes highlights potential therapeutic targets in colorectal cancer

**DOI:** 10.1186/s12935-024-03239-y

**Published:** 2024-01-31

**Authors:** Xu Wang, Shixin Chan, Jiajie Chen, Yuanmin Xu, Longfei Dai, Qijun Han, Zhenglin Wang, Xiaomin Zuo, Yang Yang, Hu Zhao, Ming Wang, Chen Wang, Zichen Li, Huabing Zhang, Wei Chen

**Affiliations:** 1 Department of General Surgery, The First Affiliated Hospital of Anhui Medical University, Hefei, 230032 Anhui China; 2 Department of Dermatology, The First Affiliated Hospital of Anhui Medical University, Hefei, 230032 Anhui China; 3 Department of Biochemistry and Molecular Biology, Metabolic Disease Research Center, School of Basic Medicine, Anhui Medical University, Hefei, 230032 Anhui China; 4 The First Affiliated Chuzhou Hospital of Anhui Medical University, Chuzhou, 239000 Anhui China

**Keywords:** Machine learning, Immune evasion, Cytotoxic T lymphocyte, Prognosis, Colorectal cancer

## Abstract

**Background:**

A minute fraction of patients stands to derive substantial benefits from immunotherapy, primarily attributable to immune evasion. Our objective was to formulate a predictive signature rooted in genes associated with cytotoxic T lymphocyte evasion (CERGs), with the aim of predicting outcomes and discerning immunotherapeutic response in colorectal cancer (CRC).

**Methods:**

101 machine learning algorithm combinations were applied to calculate the CERGs prognostic index (CERPI) under the cross−validation framework, and patients with CRC were separated into high− and low−CERPI groups. Relationship between immune cell infiltration levels, immune−related scores, malignant phenotypes and CERPI were further analyzed. Various machine learning methods were used to identify key genes related to both patient survival and immunotherapy benefits. Expression of HOXC6, G0S2, and MX2 was evaluated and the effects of HOXC6 and G0S2 on the viability and migration of a CRC cell line were in−vitro verified.

**Results:**

The CERPI demonstrated robust prognostic efficacy in predicting the overall survival of CRC patients, establishing itself as an independent predictor of patient outcomes. The low−CERPI group exhibited elevated levels of immune cell infiltration and lower scores for tumor immune dysfunction and exclusion, indicative of a greater potential benefit from immunotherapy. Moreover, there was a positive correlation between CERPI levels and malignant tumor phenotypes, suggesting that heightened CERPI expression contributes to both the occurrence and progression of tumors. Thirteen key genes were identified, and their expression patterns were scrutinized through the analysis of single−cell datasets. Notably, HOXC6, G0S2, and MX2 exhibited upregulation in both CRC cell lines and tissues. Subsequent knockdown experiments targeting G0S2 and HOXC6 resulted in a significant suppression of CRC cell viability and migration.

**Conclusion:**

We developed the CERPI for effectively predicting survival and response to immunotherapy in patients, and these results may provide guidance for CRC diagnosis and precise treatment.

**Supplementary Information:**

The online version contains supplementary material available at 10.1186/s12935-024-03239-y.

## Introduction

Colorectal cancer (CRC) constitutes a significant global menace to human health, emerging as the third most prevalent malignancy and the second foremost contributor to cancer−associated fatalities on a worldwide scale. It is widely recognized as a significant medical and health issue [1]. By 2030, the amount of CRC cases is expected to approach 2.2 million, with over 1.1 million deaths [2]. Data gleaned from cancer surveys reveals a persistent rise in CRC incidence in China. This malignancy has ascended to become the fourth most prevalent cancer and the fifth primary contributor to cancer−related mortalities in the country [3]. As of now, the precise mechanisms driving the development of CRC remain elusive. Empirical findings in the realm of evidence−based medicine propose a nuanced interconnection between CRC incidence and various factors, including genetic mutations, a diet rich in fats, inflammatory processes, immune responses, and perturbations in the gut microbiota [4]. With advancements in CRC diagnostic techniques and treatment options, patients diagnosed with early−stage CRC can attain a 5−year survival rate of up to 90% [5]. However, the symptoms of early stage CRC are often overlooked, and most patients are diagnosed at intermediate or advanced stages. Even after the removal of the primary tumor, 30–50% of cases with tumor recurrence were still observed [6]. In recent years, the emergence of targeted therapeutic modalities and the implementation of immunotherapy approaches have expanded the array of treatment alternatives available to CRC patients. Immunotherapy with the immune checkpoint inhibitor (ICI) Programmed Death Receptor 1 (PD−1) has shown efficacy in patients with mismatch repair deficiency or high microsatellite instability (MSI) in metastatic CRC [7]. However, these treatment methods benefit a minute fraction of patients. Enhancing the effectiveness of treatment for intermediate− and advanced−stage CRC is challenging for researchers. Therefore, conducting in−depth studies on the potential mechanisms underlying CRC development, identifying early diagnostic markers, and exploring treatment targets remains essential.

T cells exhibit distinctive characteristics in their anti−cancer localization, demonstrating both direct effector functions and the ability to elicit auxiliary responses through the recruitment of other immune components. Additionally, T lymphocytes can expand in vitro and establish memory compartments, which are pivotal attributes in anti−tumor surveillance [8]. Previous studies have advanced the notion that CD4+ and CD8+ T cells infiltrated into malignant tumors not only signifies the ongoing host−driven anti−tumor response, but also bears a direct association with the prognosis of patients with cancer [9, 10]. Cytotoxic T lymphocytes (CTL), often identified as CD8+ T cells, stand as pivotal agents in anti−cancer immunity and constitute the primary focus of efforts in cancer immunotherapy [11]. The resistance to immune checkpoint inhibitors arises when there is an excessive activation of CD8+ T cells, leading to their differentiation into an exhausted phenotype within the immune system [12]. In most immunotherapeutic approaches, the precise recognition and targeted elimination of tumor cells by CD8+ T cells are imperative, with immune evasion standing as the predominant factor contributing to resistance in immunotherapeutic interventions.[13]. Several previous researches have focused on exploring the potential mechanisms of immune evasion in various solid tumors. Zhang et al. [14] demonstrated that retinoic acid−inducible gene−I contributes to immune evasion by regulating the ubiquitination of PD−L1 in colon cancer. Travelli et al. [15] suggested that T cell immune evasion in breast cancer could be counteracted by extracellular nicotinamide phosphoribosyltransferase. ZNF652 acts as a potential biomarker for immunotherapy in triple−negative breast cancer because its loss is related to PD−L1−mediated immune evasion [16]. FBXL6 overexpression in hepatocytes activates immune evasion in hepatocellular carcinoma [17]. In a recent investigation [18], an extensive genome−wide CRISPR screening was conducted on diverse genetically modified mouse cancer cell lines, cultured in conjunction with CTL; they identified 182 CTL evasion−related genes (CERGs), which can increase either the susceptibility or resilience of cancer cells to CTL−induced toxicity in mouse cancer models, were identified.

In recent decades, there has been rapid advancement in the field of machine learning. It is not only widely applied in healthcare−related fields, such as drug discovery and disease diagnosis, its utilization also extends widely to other domains, including mechanics, robotics, and image recognition [19–23]. Furthermore, machine learning has been widely used in emerging technologies, such as pathomics and radiomics. Some researches [24–28] also used the combination of machine learning algorithms instead of traditional method to construct models using transcriptomic data for predicting outcome or therapeutic responses in patients with malignant tumors, and the prediction efficiency of these models was significantly improved.

This study used 31 core CERGs to perform consensus clustering for identifying two CERG−related molecular subtypes of CRC and prognosis−related differentially expressed genes (DEGs) between the two subtypes. A combination of 10 machine methods was applied to develop a prognostic signature and calculate the CTL evasion−related prognostic index (CERPI) using seven CRC cohorts. CERPI was significantly correlated with patient survival, clinical characteristics, immune cell infiltration, and malignant cancer phenotypes. To identify the key signature genes, data from seven immunotherapy clinical cohorts were used. The expression of 13 key signature genes was analyzed using bulk and single−cell data. G0S2, HOXC6, and MX2 expression was validated using qRT−PCR and immunohistochemistry, and the effects of G0S2 and HOXC6 on CRC cell viability and migration were verified in vitro.

## Materials and methods

### Data collection and processing

Transcription data, single−cell sequencing data and relevant clinical information were retrieved from The Cancer Genome Atlas (TCGA, ID: TCGA-COAD and TCGA-READ), Gene Expression Omnibus (GEO, ID: GSE17536, GSE17537, GSE29621, GSE38832, GSE39582, GSE72970, GSE100797, GSE179351, GSE35640, GSE78220, and GSE91061), Tumor Immune Dysfunction and Exclusion (TIDE) (https://tide.dfci.harvard.edu/, ID: PRJEB25780), iMvigor210 (http://research-pub.gene.com/IMvigor210CoreBiologies, ID: iMvigor210), Firehose (http://gdac.broadinstitute.org), the Xena Browser (https://xenabrowser.net/datapages/), and Tumor Immune Single-cell Hub 2 (TISCH2, http://tisch.comp-genomics.org/home/, ID: EMTAB8107, GSE108989, GSE146771, and GSE166555) databases. Among these datasets, seven (TCGA-CRC, GSE17536, GSE17537, GSE29621, GSE38832, GSE39582, and GSE72970) with complete follow-up information of patients with CRC were used to calculate the CERPI using a combination of machine learning algorithms and to evaluate the correlation between clinical characteristics, tumor microenvironment (TME), and CERPI. Seven immunotherapy-related datasets (GSE100797, GSE179351, GSE35640, GSE78220, and GSE91061, PRJEB25780, and iMvigor210) were used to construct the model for predicting immunotherapy benefits using abundant machine learning methods in patients with various cancer types. mRNA expression, copy number, DNA methylation, and mutation data of 20 cancer types were used to investigate the genetic aberrations of CERGs and evaluate the relationship between signature genes and malignant prototypes in cancers using z-score algorithms. Four single-cell datasets (EMTAB8107, GSE108989, GSE146771, and GSE166555) were used to analyze the expression levels of key genes of prognostic and predictive signatures in different single cell types. Transcription data from TCGA database were transformed from fragments per kilobase million into transcripts per million using R software (version 4.2.1). TCGA-COAD and TCGA-READ datasets were merged into TCGA-CRC cohort, six CRC datasets were merged into the GEO-Meta cohort, batch effects were mitigated through the implementation of the Combat algorithm, and the normalization and transformation of expression data were carried out using the log2 formula with the assistance of the sva R package. Patients with incomplete clinical information or survival times were excluded from this study.

### Comprehensive analyses of genetic alterations and biological functions of CERGs in cancers

CERGs were extracted from a previous study [18] and immune-related genes (IRGs) were retrieved from the ImmPort database (https://www.immport.org/shared/home), and the insertion genes between these two gene sets were identified as core CERGs. The locations of these core CERGs on the human chromosomes were analyzed and further visualized using RCircos package. Copy number variation (CNV), mRNA expression levels, differential methylation, and Pearson's correlation between the expression and methylation levels of the core CERGs were also analyzed. The assessment of the relationship between copy number segment values and expression values for each gene involved the computation of Pearson's correlation coefficient. To ascertain the mutual exclusivity of genes within each cancer type, a significance threshold of q value 0.05 was applied. The differential methylation status of individual genes in tumor and normal samples was determined through the Wilcoxon signed rank test, with genes exhibiting significant hypomethylation or hypermethylation identified based on a *p*−value cutoff of 0.05. The correlation between the transcriptional expression of CERGs and the Beta value of the promoter DNA methylation was investigated using Pearson's correlation, with significance determined by a *p*−value < 0.05. Gene Ontology (GO) and Kyoto Encyclopedia of Genes and Genomes (KEGG) analyses were executed to delve deeper into the biological functions and pathways pertinent to these CERGs using xiantao online website (www.xiantaozi.com).

### Identification of CERG-related molecular subtypes

Consensus clustering is an unsupervised clustering method, it is a common research method for cancer subtype classification. Samples can be divided into several subtypes according to different omics data, so as to identify new disease subtypes or compare and analyze between different subtypes. Utilizing the expression profiles of the 31 fundamental CERGs, the TCGA-CRC cohort underwent a stratification into two distinct clusters through the application of the consensus clustering method. Principal Component Analysis (PCA) was employed, utilizing the stats R package, to assess the discernibility between the two clusters. Subsequently, clinical attributes and the expression patterns of core CERGs within the identified clusters were visually represented in a heatmap, constructed using the pheatmap R package. Single−sample gene set enrichment analysis (ssGSEA) was conducted, employing the gsva R package, to scrutinize pathways associated with the two clusters. The immune landscape within CERG-related subtypes were explored involved the implementation of the movics R package, assessing immune-related scores, expression of immune checkpoints, and levels of immune cell infiltration between CERG-related subtypes A and B. DEGs between these subtypes were pinpointed using the limma package, with criteria set at |Fold Change| > 1.5 and an adjusted *p*−value < 0.05. Further insights into the biological functions and pathways of DEGs were gained through GO and KEGG analyses. Univariate Cox regression was applied to identify prognosis−related DEGs, warranting subsequent analysis.

### Calculation of CERPI using combination of machine learning algorithms

Transcription data of seven CRC datasets were used to calculate the CERPI via combination of machine learning methods, comprising Coxboost, partial least squares Regression for Cox (plsRcox), least absolute shrinkage and selection operator (Lasso), Elastic Network (Enet), Ridge, StepCox, Random Survival Forest, Supervised Principal Components, survival Support Vector Machine (survival−SVM), and Generalized Boosted Regression Modeling (GBM). The area C-index of each algorithm was computed and shown in the heatmap, sorted by the average C-index values in seven CRC cohorts. The algorithm demonstrating the highest average C-index values was recognized as the optimal method for predicting the overall survival (OS) of patients, which was calculated based on this optimal method using the predict function of R software. Patients within each cohort, and the GEO-Meta cohort, were stratified into high− and low-CERPI groups according to the median CERPI values. The OS of CRC patients across the seven cohorts was then compared using the Kaplan–Meier method and log-rank tests. Meta−analysis was performed to determine whether there was significant heterogeneity among the seven datasets. We also collected 56 published articles (Additional file 1: Table S1) that constructed prognostic signatures for survival prediction in patients with CRC and compared the AUC values of our CERPI with those of published signatures using two-sided t-tests.

### CERPI for clinical application

Four datasets, TCGA−CRC, GSE39582, GSE17536, and GSE72970, contain complete clinical data, including the TNM staging information, Chi−Square tests were employed to compare the clinical characteristics between groups categorized as high and low based on CERPI values and presented using pie charts. TCGA-CRC and GSE39582 datasets have the largest number of CRC patients, uni− and multi−variate cox regression analyses were performed in these two datasets to screen out independent prognostic factors, and significant factors were included to construct the nomogram model, calibration plots were utilized to assess disparities between actual survival rates and predicted survival probabilities.

TME in Different CERPI Groups

Tracking Tumor Immunophenotype (http://biocc.hrbmu.edu.cn/TIP/) is a website which uses 'ssGSEA' and 'CIBERSORT' methods to evaluate the anti-cancer immunity and immune cell abundance in malignant tumors across seven-step Cancer-Immunity Cycle. Profiling the status of anti-cancer immunity across seven-step Cancer-Immunity Cycle including release of cancer cell antigens (Step 1), cancer antigen presentation (Step 2), priming and activation (Step 3), trafficking of immune cells to tumors (Step 4), infiltration of immune cells into tumors (Step 5), recognition of cancer cells by T cells (Step 6) and killing of cancer cells (Step 7). The expression levels of biomarkers in these seven steps were compared and visualized. Cases of four TCGA-CRC representative immune subtypes [29] in two CERPI groups were drawn into a block diagram and compared using the Chi-squared method. Spearman analyses were performed to analyze the correlation between immune cell abundance, the seven-step Cancer-Immunity Cycle, and the calculated CERPI values.

### CERPI for predicting immunotherapy benefits in patients with CRC

Tumor microenvironment (TME) scores, encompassing stromal, immune, and ESTIMATE scores, were assessed in both low− and high−CERPI groups through the application of the Wilcoxon signed-rank test. TIDE scores were obtained from the TIDE website, while IPS data were acquired from The Cancer Immunome Atlas (TCIA, https://tcia.at/). The assessment of tumor immune escape probability utilized the TIDE score, where a higher score indicated an elevated likelihood of immune escape and reduced efficacy of immunotherapy. Immune Cell Proportion Scores (IPS) was employed to anticipate patient responses to diverse immune checkpoint inhibitor (ICI) therapies, encompassing PD-1/PD-L1/PD-L2, CTLA-4, and combination therapies such as PD-1/PD-L1/PD-L2 and CTLA-4 blockade, these scores between the two groups were also compared. To validate these findings, HE−stained images of TCGA-CRC cohort were retrieved from TCGA website, and the infiltrated immune cell abundance in low− and high−CERPI samples was visualized and compared.

### Evaluation of CERPI in Pan−cancer using Z−score method

Gene sets related to cancer hallmarks, including angiogenesis, epithelial to mesenchymal transition (EMT), and cell cycle were extracted from a previous study [30], and the gene sets were applied for z−score calculation using the gsva R package. The quantification of each gene set was expressed in terms of angiogenesis z−score, EMT z−score, Cell Cycle z−score, and CERPI z−score, respectively. Associations between CERPI and malignant biological processes in various cancers were analyzed using Pearson’s correlation method.

### Identification of Immunotherapy−related signature genes using abundant machine learning methods

Seven public datasets containing complete RNA−seq data and immunotherapy response information were applied to construct a binary classification model for predicting immunotherapy responses in patients with malignant tumors, patients with different immunotherapy responses were classified into Complete Response (CR)/Partial Response (PR) and Stable Disease (SD)/Progressive Disease (PD) groups. Twelve algorithms, namely, Lasso, Ridge, Enet, Stepglm, SVM, glmBoost, Linear Discriminant Analysis, plsRglm, RandomForest, GBM, XGBoost, and NaiveBayes, were used to construct the model. The C−indices of each combination of these algorithms were calculated and sorted by the Area Under Curve (AUC) value, and the genes contained in the algorithm with the highest average AUC were identified as immunotherapy−related genes. Insertion genes between the prognostic signature and immunotherapy−related genes were identified as key genes related to both patient prognosis and immunotherapy outcomes, Wilcoxon tests were employed to compare the mRNA expression levels of these genes between tumor and adjacent normal samples in the TCGA−CRC cohort.

### Analysis of the expression of key genes in different cell types using single−cell datasets

Single−cell expression matrices of the EMTAB8107, GSE108989, GSE146771, and GSE166555 datasets were downloaded from the TISCH database (http://tisch.comp-genomics.org/home/). Cellular classifications were ascribed based on the expression levels of distinct marker genes utilizing the Monaco Immune Database within the Celldex package. Subsequently, the visualization of immunotherapy−related signature genes across various cell types was undertaken for further elucidation.

### Cell culture

A human intestinal epithelial and five CRC cell lines (NCM-460, HT-29, RHO, SW620, HCT-116, and SW480) were purchased from the American Typical Culture Center. We incubated the cells in Dulbecco’s modified Eagle’s medium (DMEM) containing 10% fetal bovine serum (FBS; Lonsera, Austria) and 1% double antibiotics (streptomycin and penicillin) in 5% CO2 at 37 °C.

### RNA Isolation and qRT−PCR

Total RNA extraction was executed through the utilization of TRIzol reagent (Life Technologies, Carlsbad, CA, USA), with subsequent complementary DNA (cDNA) synthesis facilitated by a PrimeScript RT kit (Vazyme, Nanjing, China). The concentration of cDNA was quantified using TB Green Premix Ex Taq II (GenStar, Guangdong, China) and a LightCycler480 System (Applied Biosystems, Waltham, MA, United States). Relative expression levels of HOXC6, G0S2, and MX2 were determined employing the 2−ΔΔCt method, with GAPDH serving as the internal control. Differential gene expression across distinct cell lines was assessed utilizing Student’s *t*−test. Primer sequences are shown in Additional file 1: Table S2.

### Sample collection and immunohistochemistry staining

Nineteen normal colorectal tissues and 20 tumor tissues were acquired from patients who underwent surgical resection at the First Affiliated Hospital of Anhui Medical University, and subsequently preserved in formalin. Ethical approval for all experiments was granted by the Ethics Committee. Xinle Biological Company conducted the embedding, sectioning, and staining with hematoxylin and eosin. Subsequently, the sections were treated with xylene and ethanol and hydrated under running water. Antigen retrieval was conducted with a sodium citrate antigen retrieval solution (Solarbio, China). Tissue sections were subjected to incubation using a universal two−step assay kit (pv-9000; ZSGB-BIO, China) in conjunction with antibodies sourced from Zenbio, China. Subsequently, the antibody complexes were visualized using DAB, and the sections were counterstained with hematoxylin.

### Cell transfection

For the transfection of si−RNAs (TsingkeBiotech, Beijing, China) (Additional file 1: Table S3), RKO cells were cultivated until they reached 60% confluency, and transfection was carried out using Lipofectamine 3000 (Invitrogen, Shanghai, China), following the manufacturer's recommended protocols. After a 72−h incubation period and thorough washing, the cells were prepared for subsequent experimental procedures.

### Western blotting

Protein extraction was performed using RIPA buffer (Beyotime, China), supplemented with protease and phosphatase inhibitors. Western blotting procedures adhered to established protocols, as previously described [31]. The primary antibodies sourced from Zenbio, China, comprised anti-G0S2 and anti-HOXC6.

### Cell viability assay

A total of 1500 cells were meticulously dispensed into individual wells of 96−well plates. Subsequently, the cells were cultured for 0, 24, 48, or 72 h in the presence of siRNA. Following that, the cells were subjected to exposure to the CCK-8 solution (C0038, Beyotime, Shanghai, China) for an additional hour. Cell viability was evaluated by quantifying the optical density at 450 nm. The results were analyzed using *t*−tests in GraphPad Prism software (version 9.4).

### Colony formation assay

To elucidate the impact of G0S2 and HOXC6 expression on the proliferation of human CRC cells, RKO cells that had undergone transfection (1000 cells per well) were seeded into six−well plates. After a 10−day incubation period, colony formation was quantified.

### Wound healing assay

RKO cells were plated in six−well plates at a density of 1.5 × 106 cells per well. Upon cellular adhesion to the well surface, a precise scratch was made using the tip of a 200 μL pipette. Throughout the experiment, cells were cultured in DMEM supplemented with 2% FBS. Observations and photomicrographs of the scratch areas were captured at distinct time points, specifically at 0 and 24 h post PBS wash. To quantitatively assess cell migration, the distances covered within the scratch after 24 h were computed using ImageJ software.

### Transwell assay

Transwell chambers (Corning, NY, USA) were used to conduct the migration experiments. RKO cells, having undergone prior transfection, were prepared at a concentration of 5 × 104 cells and suspended in 200 μL of serum−free medium. Subsequently, these cells were introduced into the upper chambers of the Transwell system, while the lower chambers were filled with medium containing 10% FBS. After a 48−h incubation period, the cells residing in the upper chambers were meticulously eliminated, and the cells on the opposing side of the membrane were fixed using a 4% formaldehyde solution. Following fixation, the cells were stained with crystal violet, and their microscopic images were captured.

## Results

### Genetic alterations and biological functions of CERGs in cancers

**Fig. 1:**
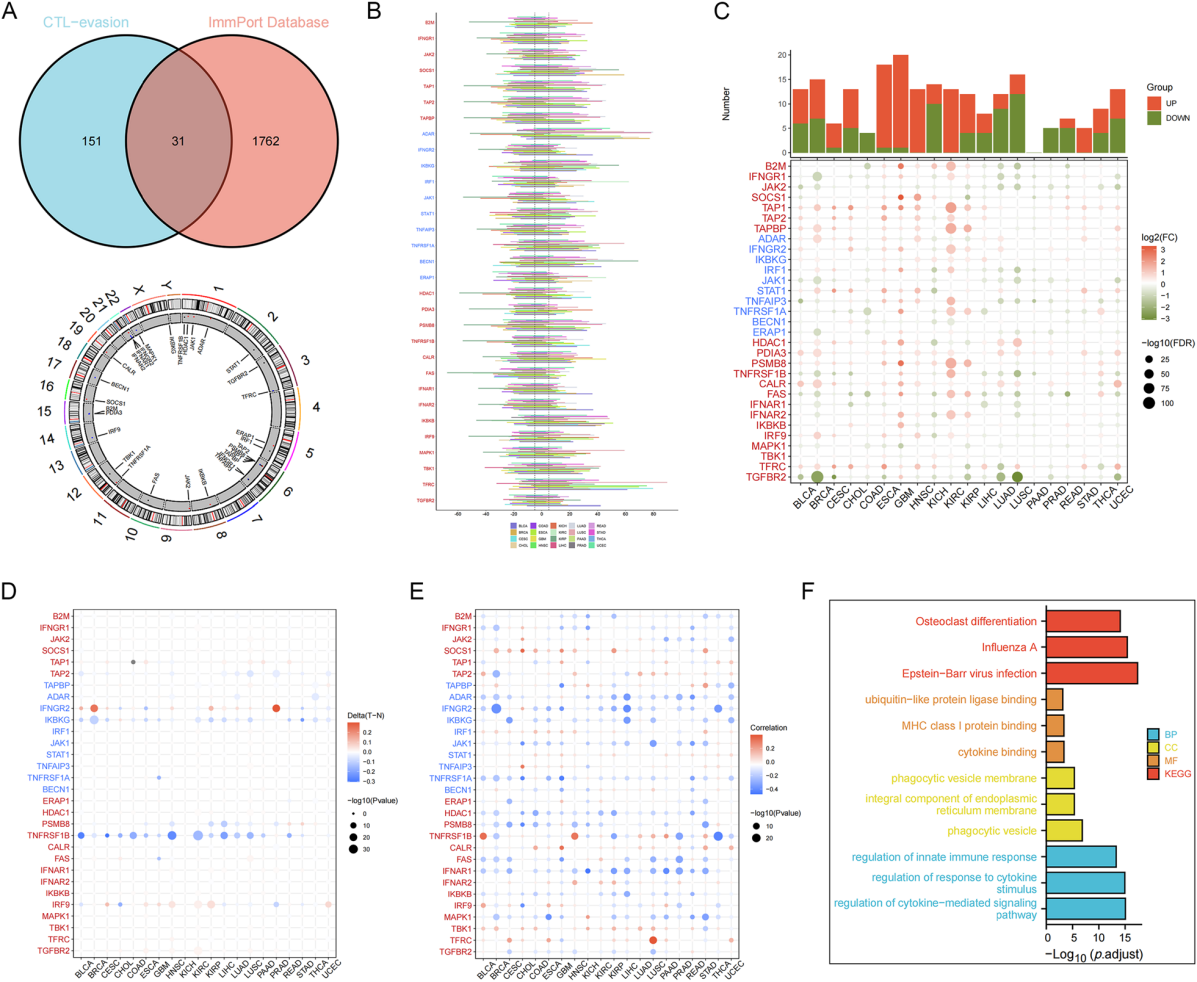
Genetic alterations and biological functions of CERGs in cancers. **A** The locations of CNVs in these genes on human chromosomes; **B** The findings revealed a pervasive trend with somatic copy number alterations manifesting at notably elevated frequencies, affecting a substantial portion of the samples across a wide spectrum of cancer types; **C** Most core CERGs exhibited distinct expression profiles in at least one cancer type; **D** DNA methylation patterns of core CERGs in the 20 cancer types; **E** Correlation between gene expression levels and DNA methylation status; **F** GO and KEGG analyses unveiled that these 31 genes predominantly participated in biological functions and pathways associated with the immune system

A total of 182 CTL−evasion−related genes and 1793 IRGs were observed (Additional file 1: Table S4), 31 core CERGs were screened, and the locations of CNVs in these genes on human chromosomes are shown (Fig. 1A). The findings revealed a pervasive trend with somatic copy number alterations manifesting at notably elevated frequencies, affecting a substantial portion of the samples across a wide spectrum of cancer types (Fig. 1B). Our investigation also revealed that most core CERGs exhibited distinct expression profiles in at least one cancer type. Notably, certain genes displayed consistent expression patterns when subjected to the cross−cancer analysis. Specifically, TAP1, TAP2, TAPBP, PSMB8, and CALR were significantly upregulated in 17, 14, 13, 15, and 15 distinct cancer types, respectively (Fig. 1C). Conversely, JAK2 was downregulated in 17 cancer types. The DNA methylation patterns of core CERGs in the 20 cancer types were also observed, and some of these genes, such as IKBKG and TNFRSF1B, showed consistent hypomethylation (Fig. 1D). While variations in the methylation patterns of core CERGs were evident, a consistent inverse correlation was observed between gene expression levels and DNA methylation status (Fig. 1E). Results of GO and KEGG analyses unveiled that these 31 genes predominantly participated in biological functions and pathways associated with the immune system (Fig. 1F). Moreover, Expression and prognostic significance of 31 core CERGs in TCGA−CRC dataset are shown in Additional file 1: Figure S1.

### Identification of CERGs−related molecular subtypes

**Fig. 2:**
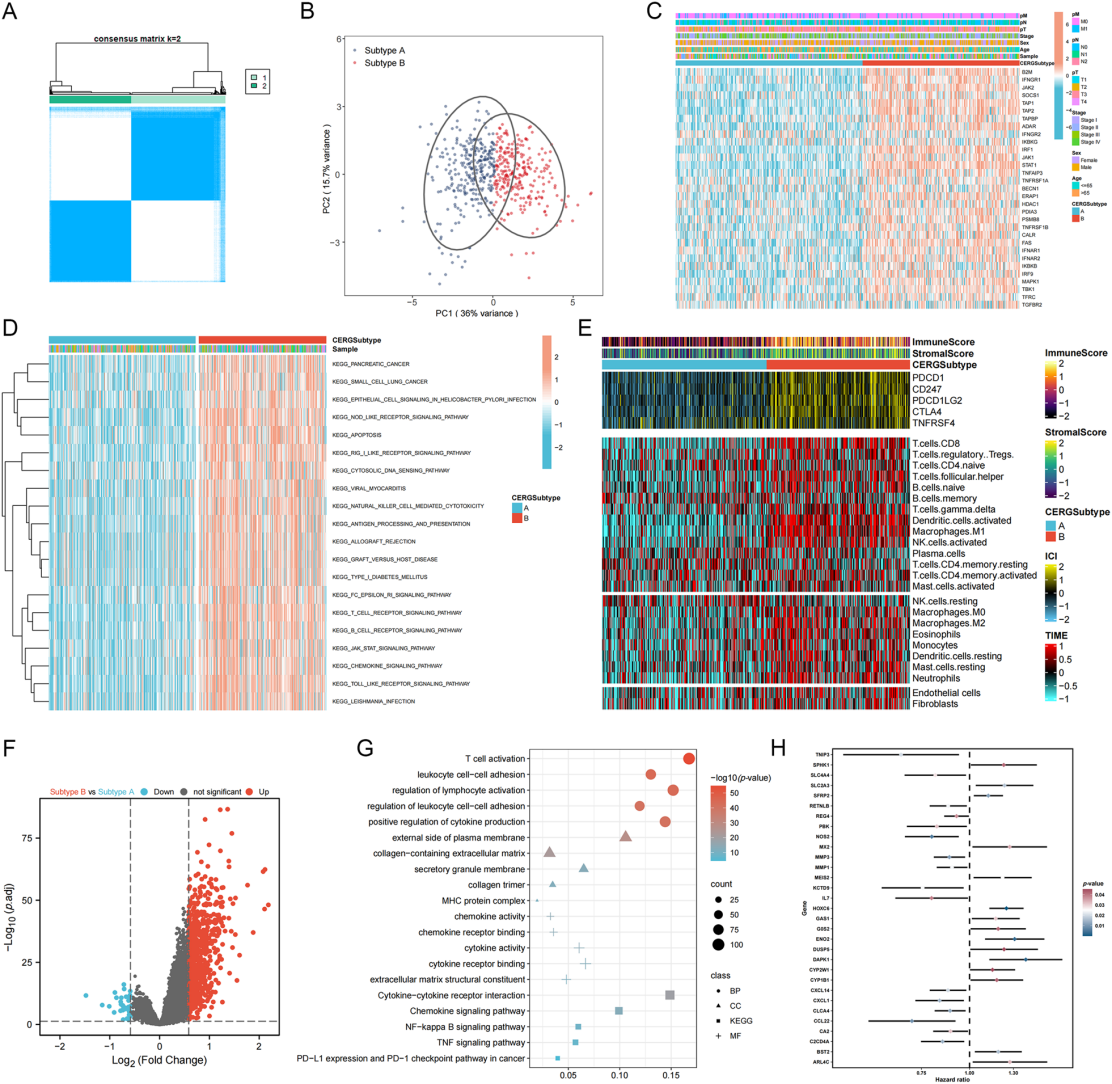
Identification of CERGs−related molecular subtypes. **A** Patients from TCGA−CRC datasets were divided into two distinct subtypes, **A** and **B**; **B** PCA revealed a good distinction between the two subtypes; **C** Associations between the subtypes, CERGs expression, and clinical features; **D** Subtype B showed higher expression levels of CERGs, and many cancer− and immune−related pathways were enriched in subtype B; **E** The TME status of the two subtypes; **F** DEGs between the two subtypes were screened out; **G** These DEGs were mainly related to immune−related biological functions, cellular components, molecular functions, and pathways; **H** 31 prognosis−related DEGs were finally identified after selection using Univariate Cox method

Based on the expression of 31 core CERGs, patients from TCGA-CRC datasets were divided into two distinct subtypes, A and B (Fig. 2A). PCA revealed a good distinction between the two subtypes (Fig. 2B). Associations between the subtypes, CERGs expression, and clinical features are shown in a heatmap (Fig. 2C). Subtype B showed higher expression levels of CERGs, and many cancer− and immune−related pathways were enriched in subtype B (Fig. 2D). The TME status of the two subtypes was also evaluated, and subtype B showed higher TME scores, immune checkpoint expression, and immune cell infiltration levels (Fig. 2E). DEGs between the two subtypes were screened out (Fig. 2F). These DEGs were mainly related to immune−related biological functions, cellular components, molecular functions, and pathways (Fig. 2G). 31 prognosis−related DEGs were finally identified after selection using Univariate Cox method (Fig. 2H).

### Calculation of CERPI using combination of 10 machine learning algorithms

**Fig. 3:**
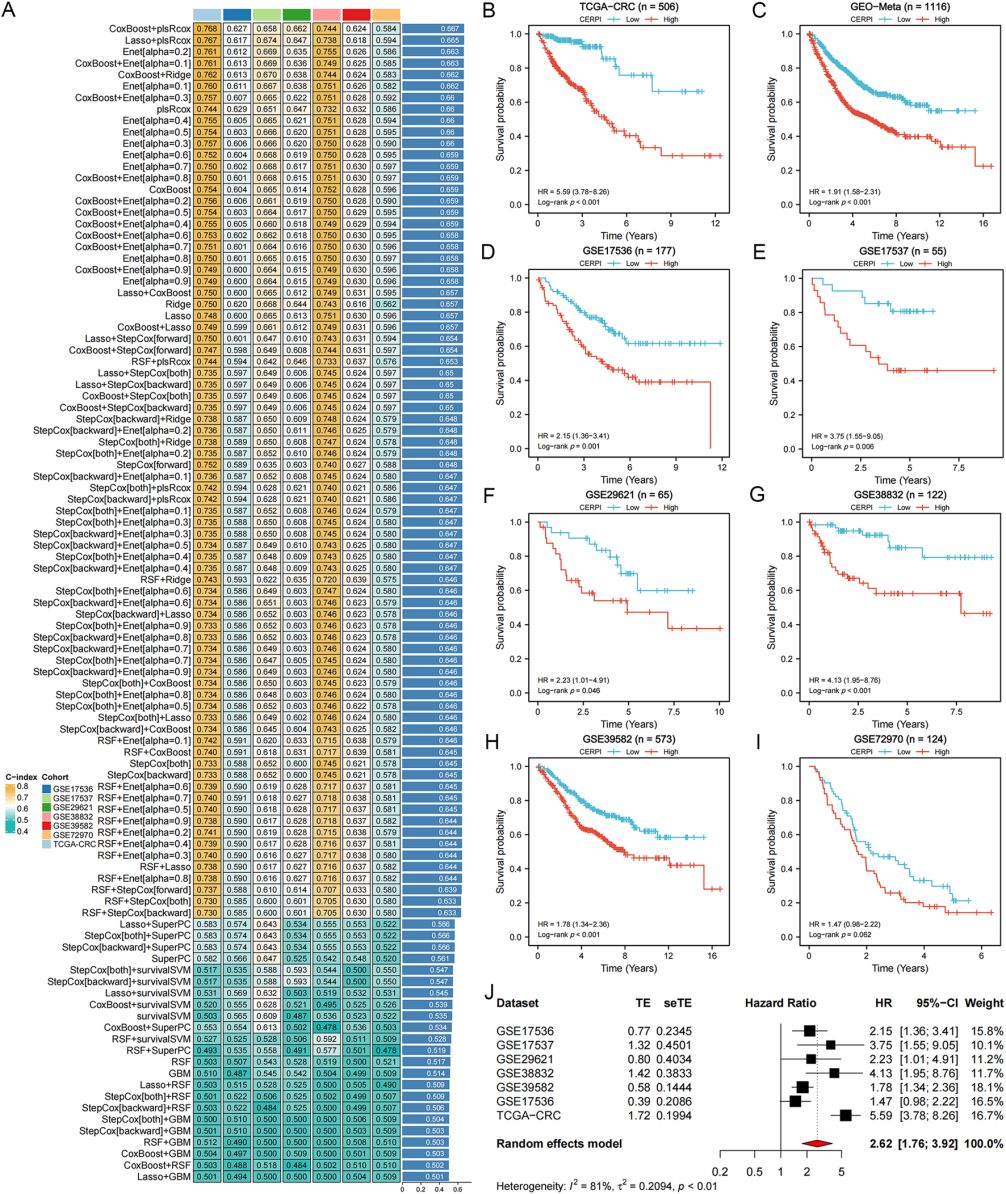
Calculation of CERPI using combination of 10 machine learning algorithms. **A** comprehensive analysis involving 101 machine learning algorithms was conducted to compute the CERPI; **B**−**I** Patients within the high−CERPI group exhibited significantly shorter OS durations compared to those in the low−CERPI group across multiple datasets; **J** The results of the meta−analysis did not show any evidence of heterogeneity among these seven CRC cohorts

**Fig. 4:**
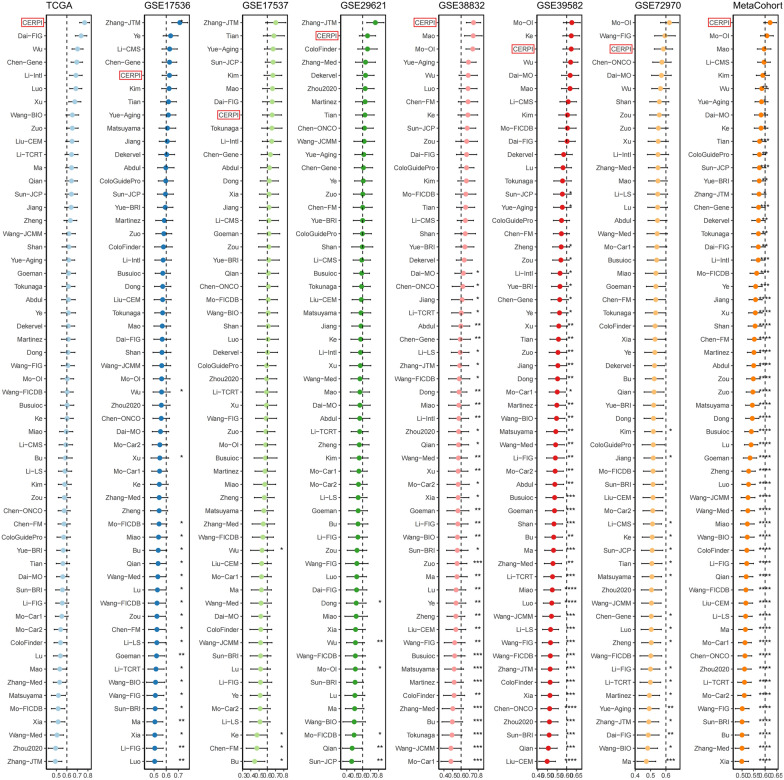
A comparative analysis of the C−index for the CERPI in relation to the other 56 published signatures was conducted. Remarkably, the CERPI consistently outperformed nearly all other models across all examined datasets. *p < 0.05; **p < 0.01; ***p < 0.001; ****p < 0.0001

A comprehensive analysis involving 101 machine learning algorithms was conducted to compute the CERP. The algorithms were organized based on their average C−index values in colorectal cancer cohorts (Additional file 1: Table S5). Coxboost + plsRcox had the highest average C−index value, 0.667, and was selected as the optimal method, and CERPI was calculated based on this algorithm (Fig. 3A). Patients within the CRC cohorts were stratified into groups categorized as low−CERPI and high−CERPI based on the median value of CERPI. Patients within the high−CERPI group exhibited significantly shorter OS durations compared to those in the low−CERPI group across multiple datasets (HR = 5.59, *p* < 0.001, Fig. 3B), GEO-Meta (HR = 1.91, *p* < 0.001, Fig. 3C), GSE17536 (HR = 2.15, *p* = 0.001, Fig. 3D), GSE17537 (HR = 3.75, *p* = 0.006, Fig. 3E), GSE29621 (HR = 2.23, *p* = 0.046, Fig. 3F), GSE38832 (HR = 4.13, *p* < 0.001, Fig. 3G), GSE39582 (HR = 1.78, *p* < 0.001, Fig. 3H), and GSE72970 (HR = 1.47, *p* = 0.062, Fig. 3I) datasets. Moreover, the results of the meta−analysis did not show any evidence of heterogeneity among these seven CRC cohorts (Fig. 3J). Furthermore, we conducted a comparative analysis of the C-index for the CERPI in relation to the other 56 published signatures (Fig. 4). Remarkably, the CERPI consistently outperformed nearly all other models across all examined datasets. the majority of models exhibited commendable performance within their respective training datasets but displayed relatively diminished performance in external datasets. This observation may be ascribed to the limited generalizability of the models, often stemming from overfitting. In contrast, our signature underwent dimensionality reduction using two machine learning algorithms, consequently enhancing its potential for extrapolation across diverse datasets.

### CERPI for clinical application

**Fig. 5:**
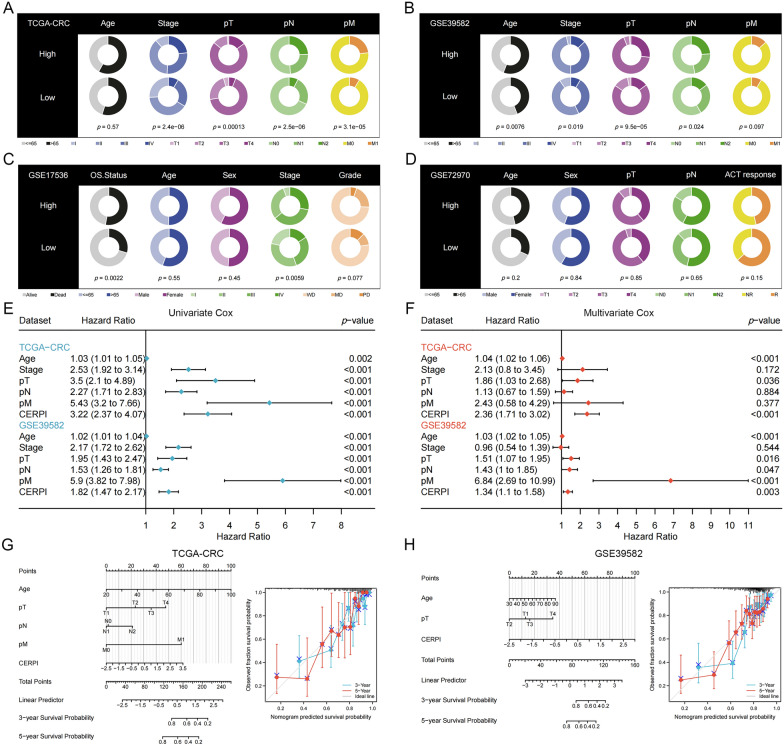
CERPI for clinical applications. The high−CERPI patients were more likely to have advanced−stage CRC in TCGA−CRC (*n* = 506) (**A**), GSE39582 (*n* = 573) (**B**), and GSE17536 (*n* = 177) (C) datasets; however, this trend was not significant in the GSE72970 (n = 124) dataset (**D**). TCGA−CRC and GSE39582 datasets were used to perform univariate (**E**) and multivariate (**F**) Cox regression analyses. Clinical features that exhibited significant associations with patient prognosis in both univariate and multivariate analyses were utilized in the construction of the nomogram model. Calibration plots demonstrated a high level of prediction accuracy for the nomograms in the TCGA−CRC dataset (**G**) and the GSE39582 dataset (**H**)

The high−CERPI patients were more likely to have advanced−stage CRC in TCGA-CRC (Fig. 5A), GSE39582 (Fig. 5B), and GSE17536 (Fig. 5C) datasets; however, this trend was not significant in the GSE72970 dataset (Fig. 5D). TCGA−CRC and GSE39582 contained the largest number of patients with CRC and complete clinical information, and these two datasets were used to perform univariate (Fig. 5E) and multivariate (Fig. 5F) Cox regression analyses. The results suggested that CERPI remained significantly related to patient OS, indicating that CERPI is an independent predictor of patient OS. Clinical features that exhibited significant associations with patient prognosis in both univariate and multivariate analyses were utilized in the construction of the nomogram model. Calibration plots demonstrated a high level of prediction accuracy for the nomograms in the TCGA−CRC dataset (Fig. 5G) and the GSE39582 dataset (Fig. 5H).

### TME in different CERPI groups

**Fig. 6:**
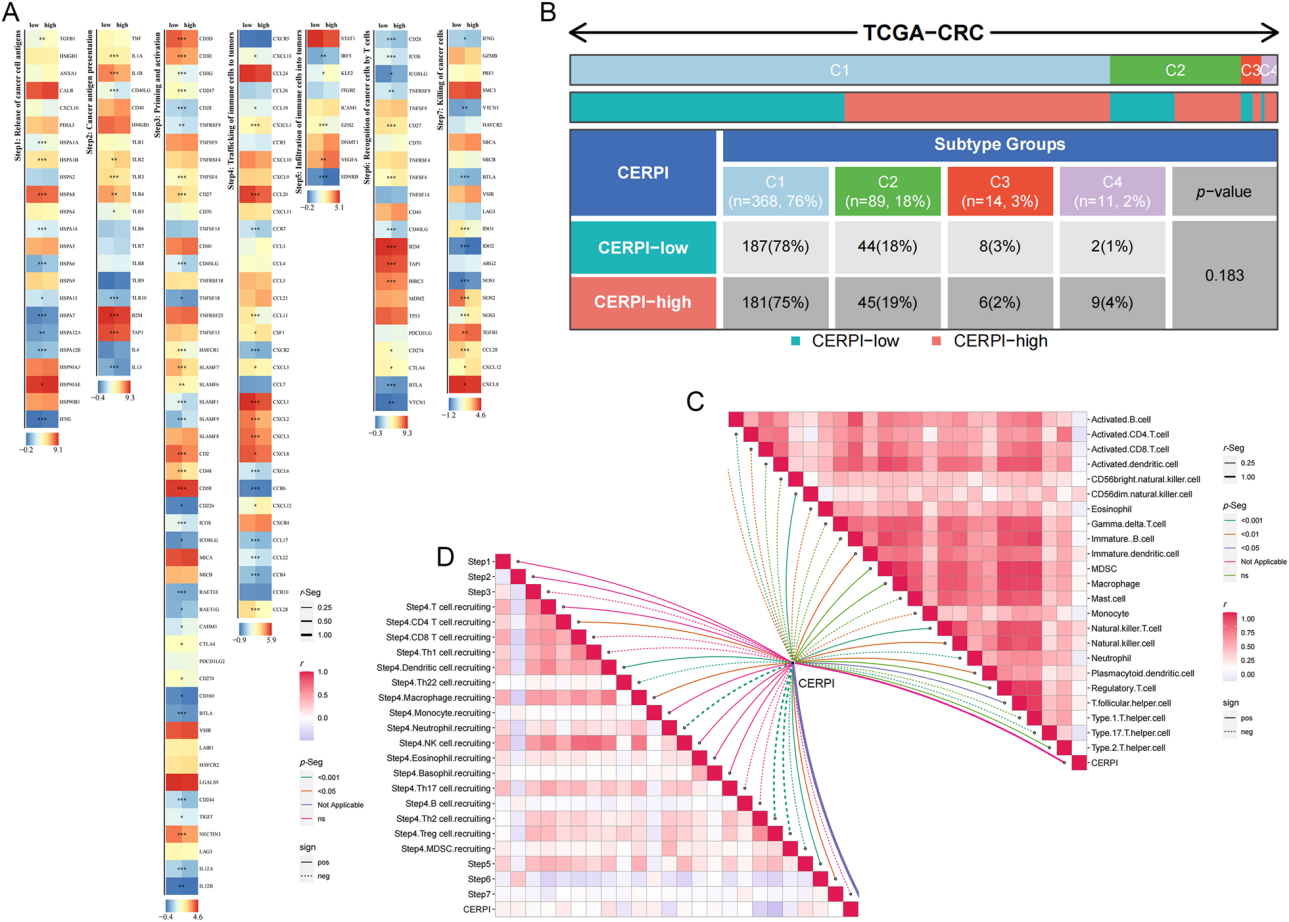
Relationship between CERPI and seven−step Cancer−Immunity Cycle, immune subtypes, and immune cell infiltration. **A** The majority of marker genes associated with the seven−step Cancer−Immunity Cycle displayed varying expression levels between the two groups; **B** There were no significant differences observed in the proportions of immune subtypes between the two groups; **C** Correlation between CERPI and the seven−step Cancer−Immunity Cycle; **D** Relationship between CERPI and immune cell infiltration levels. *p < 0.05; **p < 0.01; ***p < 0.001

**Fig. 7:**
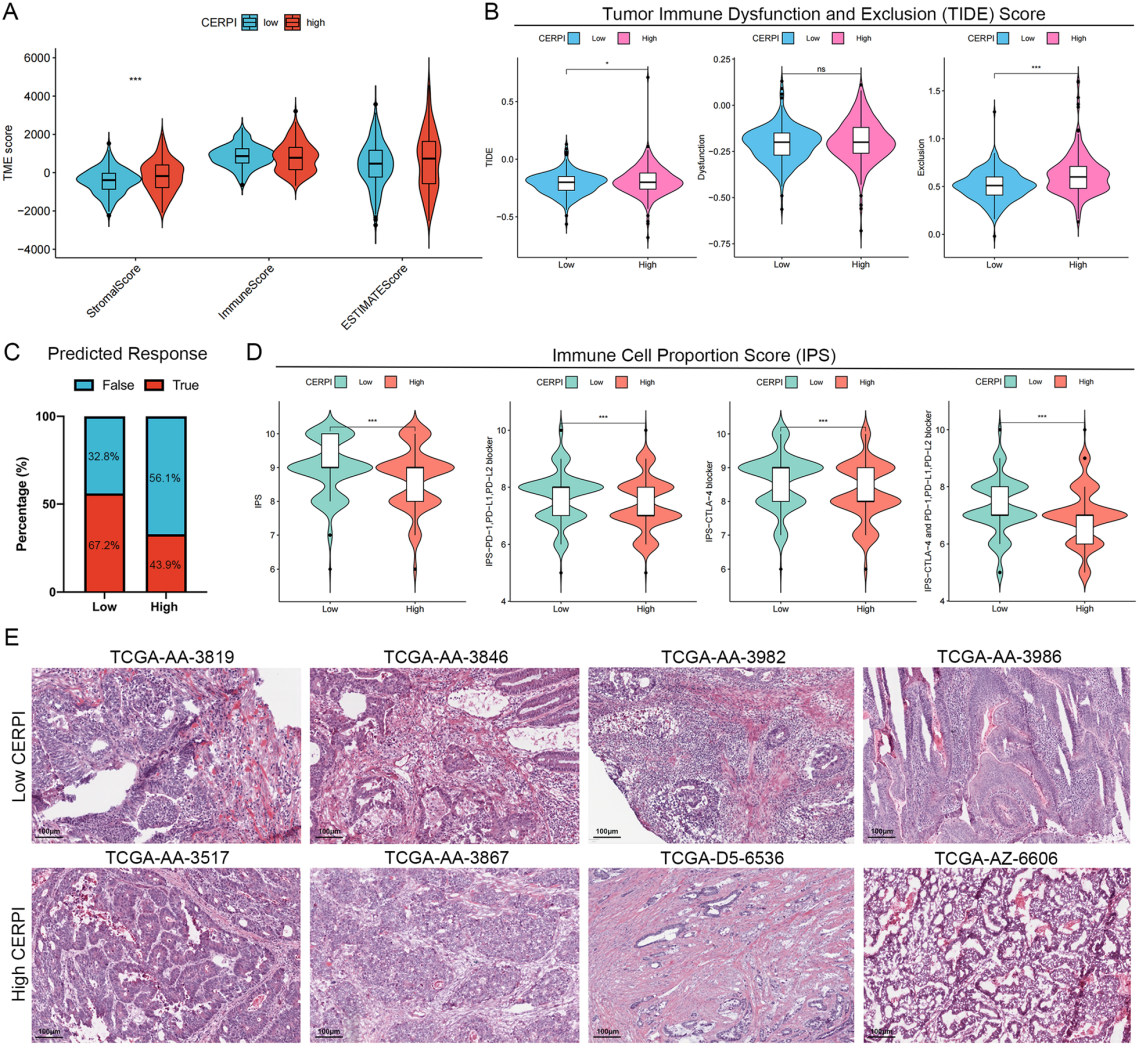
Immune−related scores revealed different immunotherapy benefits in high− and low−CERPI groups. **A** The high−CERPI group demonstrated elevated stromal scores compared to the low−CERPI group; **B** Within the high−CERPI group, patients exhibited heightened TIDE and immune exclusion scores; **C** The TIDE algorithm was further employed to predict patient responses to immune checkpoint blockade therapy, revealing a higher proportion of responders in the low−CERPI group; **D** The low−CERPI patients had a higher IPS after receiving PD−1, PD−L1, PD−L2, and CTLA−4 monotherapy or combination therapy; **E** HE staining of slides from patients in TCGA−CRC cohort showed more immune cells infiltration around tumor cells in low−CERPI patients than in high−CERPI patients. ns *p* > 0.05; *p < 0.05; ***p < 0.001

The majority of marker genes associated with the seven−step Cancer-Immunity Cycle displayed varying expression levels between the two groups (Fig. 6A). Nevertheless, there were no significant differences observed in the proportions of immune subtypes between the two groups (Fig. 6B). CERPI exhibited a positive correlation with the infiltration levels of CD56dim natural killer, natural killer T, plasmacytoid, and T follicular helper cells, and negatively correlated with the infiltration levels of activated B, CD4+, and CD8+ T cells; eosinophils; monocytes; neutrophils; and type 17 T helper cells (Fig. 6C). Moreover, CERPI was positively correlated with dendritic cell recruitment, macrophage recruitment, infiltration of immune cells into tumors, and recognition of cancer cells by T cells and negatively associated with recruitment of neutrophils and Th22, Th2, Treg, and myeloid−derived suppressor cells (MDSCs) (Fig. 6D). The high-CERPI group demonstrated elevated stromal scores compared to the low-CERPI group (Fig. 7A). Within the high-CERPI group, patients exhibited heightened TIDE and immune exclusion scores, indicating an increased likelihood of immune evasion, while patients in the low-CERPI group were more prone to benefit from immunotherapy (Fig. 7B). The TIDE algorithm was further employed to predict patient responses to immune checkpoint blockade therapy, revealing a higher proportion of responders in the low-CERPI group (Fig. 7C). The low-CERPI patients had a higher IPS after receiving PD-1, PD-L1, PD-L2, and CTLA-4 monotherapy or combination therapy, indicating better therapeutic responses (Fig. 7D). HE staining of slides from patients in TCGA-CRC cohort showed more immune cells infiltration around tumor cells in low-CERPI patients than in high-CERPI patients (Fig. 7E).

### Evaluation of CERPI in various cancer types

**Fig. 8:**
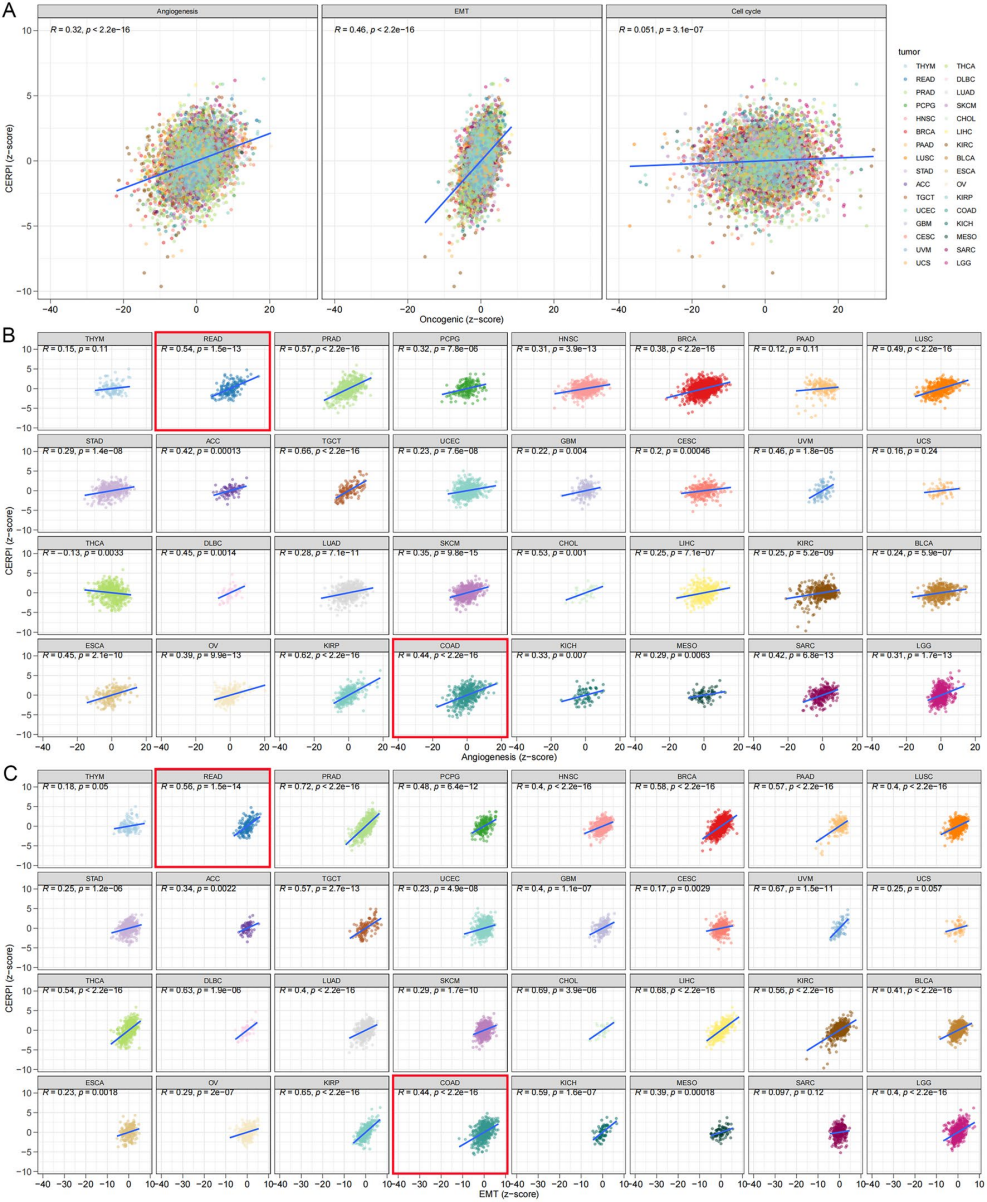
Evaluation of CERPI in various cancer types. Positive correlations between the CERPI and angiogenesis, EMT, as well as cell cycle progression (**A**) were observed. Associations between angiogenesis (**B**), EMT (**C**), and CERPI in 32 different cancer types are also shown

The findings indicated a positive correlation between the CERP and angiogenesis (Fig. 8A), Epithelial−Mesenchymal Transition (EMT) (Fig. 8B), as well as cell cycle progression (Fig. 8C). Associations between angiogenesis, EMT, and CERPI in 32 different cancer types are also shown (Fig. 8D–E).

### Identification of immunotherapy−related signature genes

**Fig. 9:**
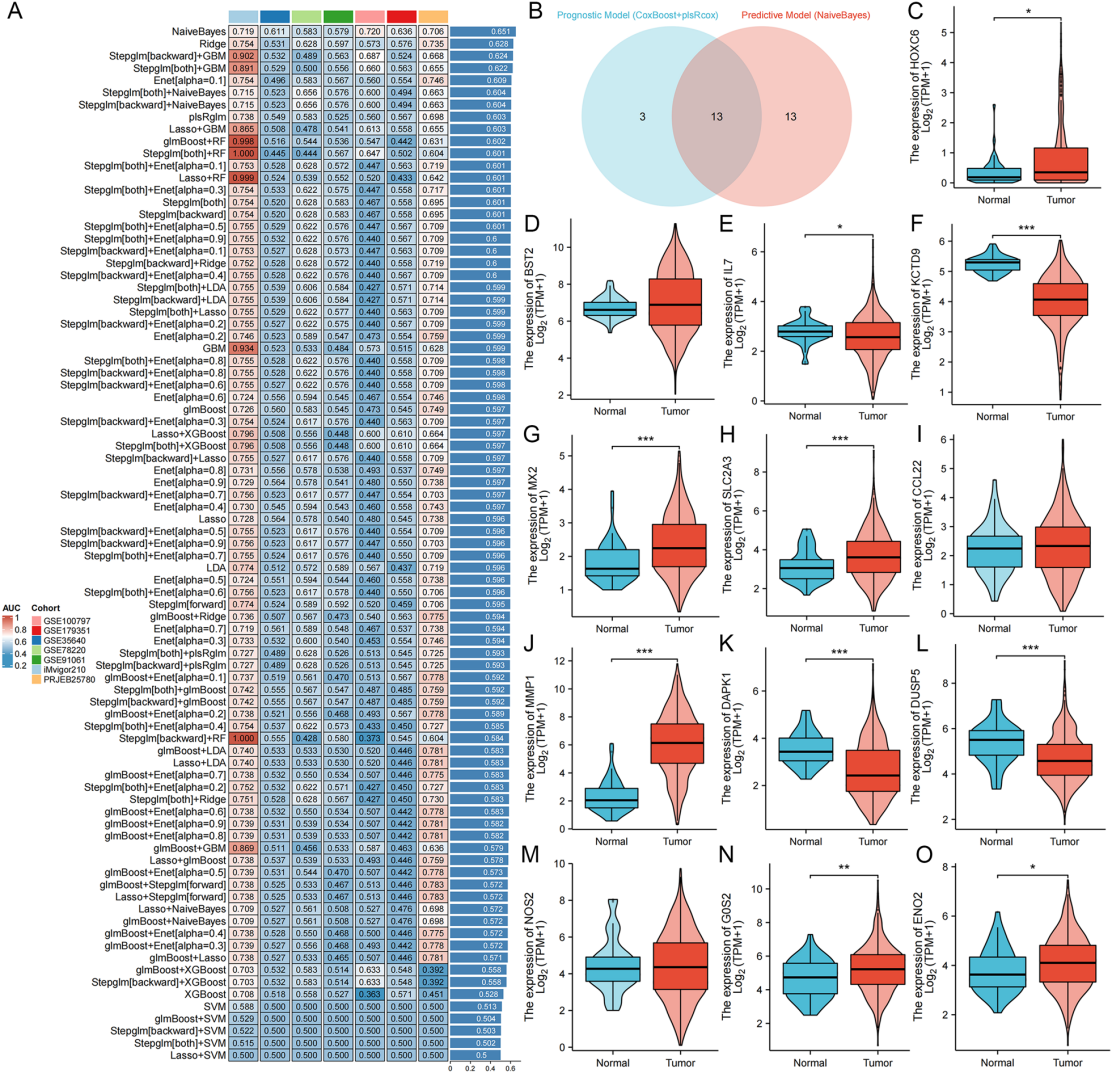
Identification of immunotherapy−related signature genes. To discern genes relevant to immunotherapy, a composite of machine learning methods was employed to formulate a model predicting the efficacy of immunotherapy across seven distinct clinical immunotherapy cohorts. NaiveBayes was identified as the optimal algorithm, with the highest average AUC value of 0.651 (**A**). Thirteen insertion genes in the prognostic− and immunotherapy−related signatures were identified as key genes (**B**). The majority of key genes exhibited differential expression between normal and tumor samples sourced from the TCGA database (**C**−**O**). *p < 0.05; **p < 0.01; ***p < 0.001

To discern genes relevant to immunotherapy, a composite of machine learning methods was employed to formulate a model predicting the efficacy of immunotherapy across seven distinct clinical immunotherapy cohorts (Additional file 1: Table S6). NaiveBayes was identified as the optimal algorithm, with the highest average AUC value of 0.651 (Fig. 9A). Thirteen insertion genes in the prognostic− and immunotherapy−related signatures were identified as key genes (Fig. 9B). The majority of key genes exhibited differential expression between normal and tumor samples sourced from the TCGA database. (Fig. 9**C**–**O**).

### Expression of key genes in different cell types

**Fig. 10:**
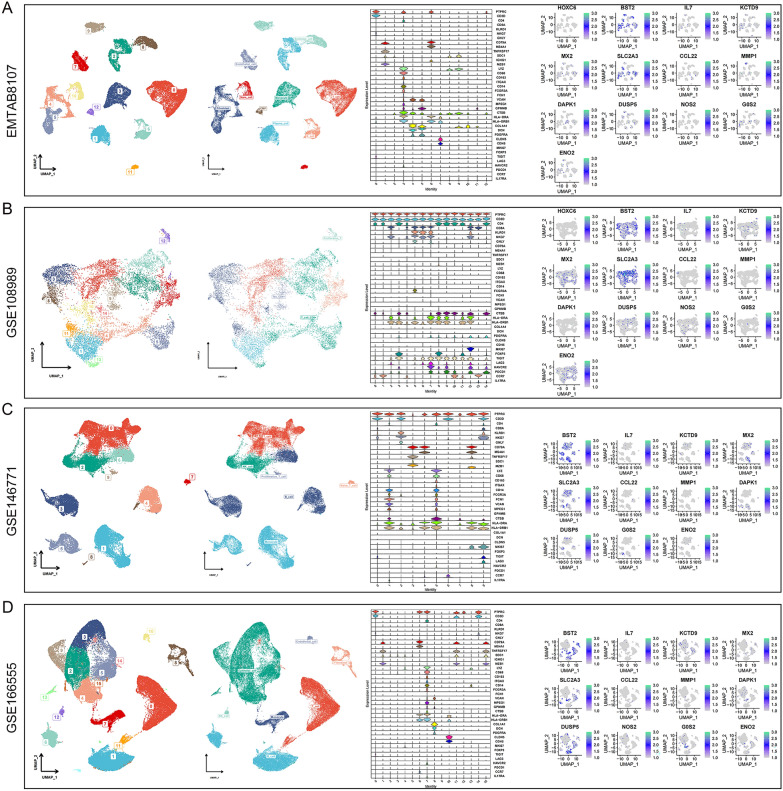
Expression of key genes in different cell types. Analysis of expression levels for 13 key genes across various cell types was conducted using four single−cell datasets. Cell type annotations were based on 39 marker genes, and the expression patterns of the 13 key genes in EMTAB8107 (**A**), GSE108989 (**B**), GSE146771 (**C**), and GSE166555 (**D**) were also delineated

Analysis of expression levels for 13 key genes across various cell types was conducted using four single−cell datasets. Cell type annotations were based on 39 marker genes, and the expression patterns of the 13 key genes in EMTAB8107 (Fig. 10A), GSE108989 (Fig. 10B), GSE146771 (Fig. 10C), and GSE166555 (Fig. 10D) were also delineated.

### Expression of HOXC6, G0S2, and MX2 in CRC Cell Lines and Tissues

**Fig. 11:**
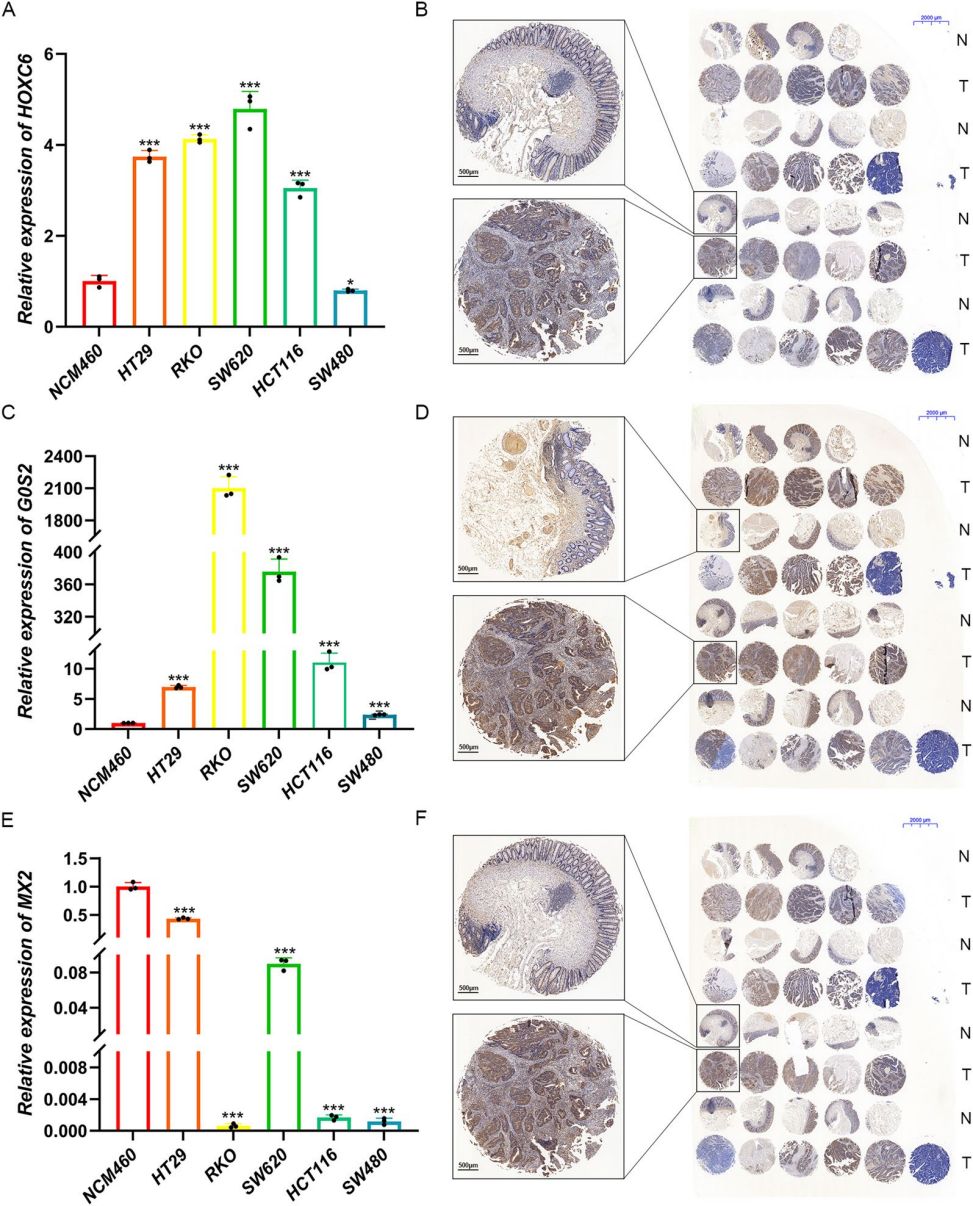
Expression of HOXC6, G0S2, and MX2 in CRC cell lines and tissues. HOXC6 (**A**) and G0S2 (**C**) were upregulated in most CRC cell lines compared to the intestinal epithelial cell line, whereas MX2 (**E**) was significantly downregulated. Nineteen normal colorectal tissues and 20 tumor tissues were colloected, all three genes showed higher protein expression levels in CRC tissues than in normal tissues (**B**, **D**, and **F**). *p < 0.05; ***p < 0.001

The mRNA levels of three genes in the human intestinal epithelial and five CRC cell lines were tested using qRT−PCR, and the protein expression levels of these genes in tumor and adjacent normal tissues were evaluated via immunohistochemical staining. HOXC6 (Fig. 11A) and G0S2 (Fig. 11C) were upregulated in most CRC cell lines compared to the intestinal epithelial cell line, whereas MX2 (Fig. 11E) was significantly downregulated. All three genes showed higher protein expression levels in CRC tissues than in normal tissues (Fig. 11B, D, and F; Additional file 1: Figure S2).

### Knocking down HOXC6 and G0S2 inhibited proliferation and migration in RKO cell line

**Fig. 12:**
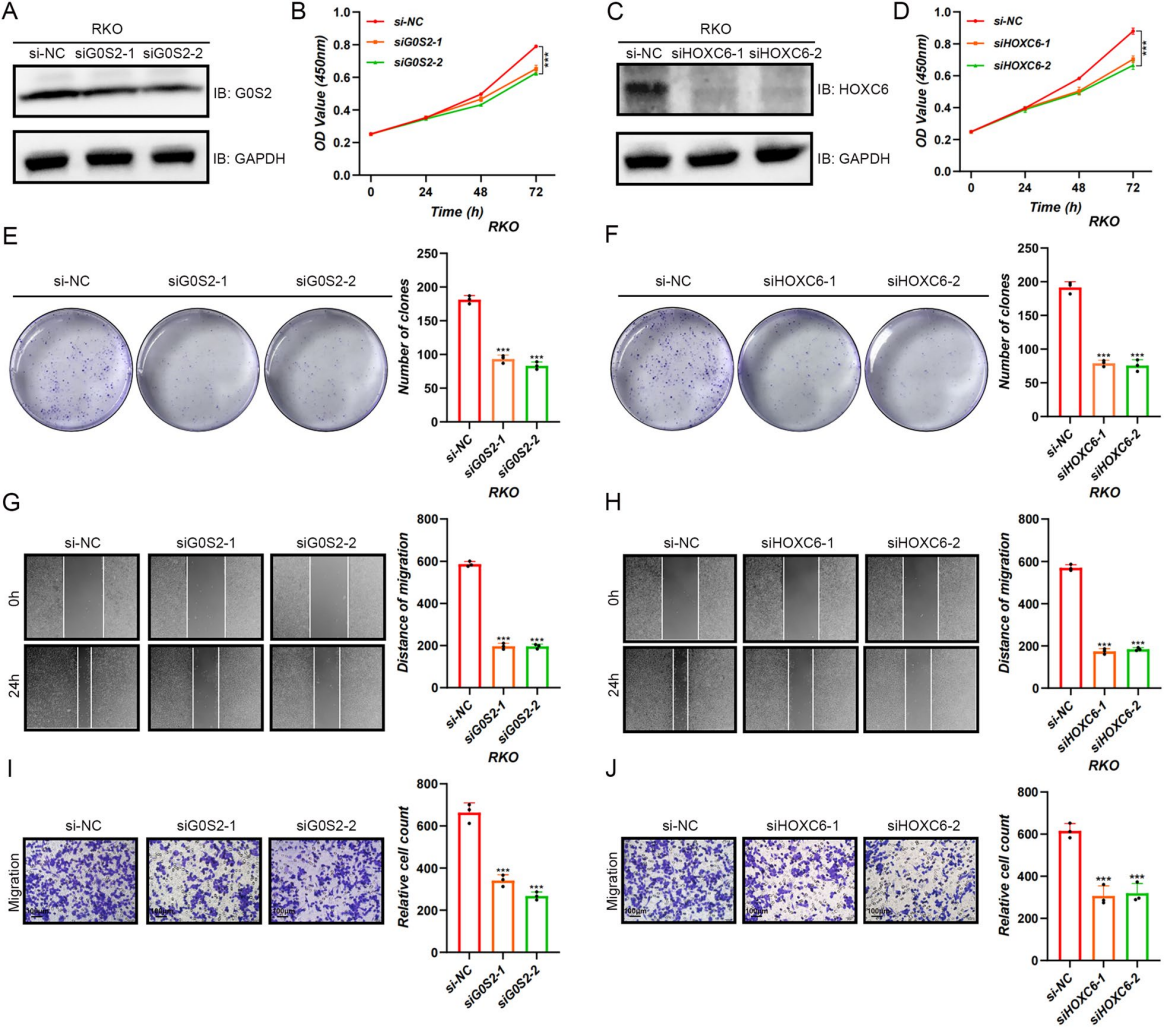
Knocking down HOXC6 and G0S2 inhibited proliferation and migration in RKO cell line. The protein expression of HOXC6 (**A**) and G0S2 (**C**) was decreased using two different si−RNA sequences. The results of the CCK−8 (*n* = 5) (**B** and **D**) and clone formation (**E**–**F**) experiments suggest that knocking down HOXC6 and G0S2 significantly inhibited the proliferative abilities of the RKO cell line. Wound healing assays. (*n* = 3) (**G**−**H**) and transwell assays (*n* = 3) (**I**–**J**) showed that the migration of the RKO cell line was suppressed after the knockdown of HOXC6 and G0S2. ***p < 0.001

Since the effects of HOXC6 and G0S2 on CRC cells have not been well studied, the protein expression of HOXC6 (Fig. 12A) and G0S2 (Fig. 12C) was decreased using two different si−RNA sequences. The results of the CCK-8 (Fig. 12B, D) and clone formation (Fig. 12E, F) experiments suggest that knocking down HOXC6 and G0S2 significantly inhibited the proliferative abilities of the RKO cell line. Wound healing assays (Fig. 12G, H) and transwell assays (Fig. 12I, J) revealed that the migration of the RKO cell line was inhibited following the knockdown of HOXC6 and G0S2.

## Discussion

Numerous serum and pathological indicators, including carcinoembryonic antigen (CEA) and TNM staging systems, have found widespread clinical application in the diagnosis, treatment guidance, and outcome prediction for patients with colorectal cancer (CRC). However, these indicators lack precision in predicting patient survival and therapeutic outcomes. In addressing this issue, additional biomarkers for CRC have been identified, such as tumor burden mutations, MSI, and neoantigen load. Despite their recognition, the predictive capacities of these methods are constrained by their low prevalence in the population or moderate effectiveness [32–34]. Therefore, developing a new approach for predicting outcomes and guiding clinical therapy that can be used in most patients with CRC is of great importance.

In this study, CNV, expression levels, differential methylation, and related biological functions of 31 core CERGs were analyzed. Most of these genes were differentially expressed and mainly correlated with immune responses. Using the gene expression profiles, patients from the TCGA-CRC dataset were stratified into two distinct molecular subtypes. Subtype B had higher immune checkpoints expression and immune cell abundance, subtype B may exhibit a more favorable response to ICI therapy. DEGs between two subtypes were identified, GO and KEGG analyses revealed that these DEGs might participate in biological processes and pathways related to immune evasion. In a study by Yamamoto et al. [35], it was proposed that autophagy might play a role in immune evasion in pancreatic cancer through the degradation of MHC-I. Immune evasion can also be promoted by Arid5a through enhancing chemokine expression [36]. Kearney et al. [37] demonstrated that tumor immune evasion can be caused by loss of TNF sensitivity. Combination of 10 machine learning methods were used for developing the CERG related signature, CoxBoost combined with plsRcox method had the highest average C-index compared with signatures constructed using traditional Lasso-Cox method [38–40], and it showed higher efficiency in predicting patient outcome. Furthermore, 58 published signatures related to different tumor phenotypes were collected, which showed higher AUC values than most published signatures, and optimal efficiency was observed for TCGA-CRC, GSE38832, and Metacohort. Prognostic signatures are rarely applied for clinical use because of overfitting and poor performance in external validation cohorts; The TCGA-CRC dataset served as the training cohort, while the validation of the model was conducted on six additional CRC datasets, and various machine learning algorithms were applied, which made the prediction performance much more reliable and robust. CERPI also correlated with clinical characteristics, including OS status and TNM staging. Cox regression analyses showed that CERPI emerged as an independent predictor of survival for CRC patients. Nomograms serve as widely employed predictive tools within the realm of oncology, particularly in the context of forecasting cancer prognosis [41, 42]. Using these variables, nomogram models were constructed and verified using calibration plots.

TME comprises cellular components such as stromal cells, endothelial cells, immune cells, and noncellular components [43]. It assumes a pivotal role in the initiation and advancement of tumors, along with influencing chemotherapy resistance in these malignancies [44]. The condition of anti−cancer immunity was characterized through the delineation of a seven−step Cancer-Immunity Cycle, including the release of cancer cell antigens, cancer antigen presentation, priming and activation, trafficking of immune cells to tumors, infiltration of immune cells into tumors, recognition of cancer cells by T cells, and killing of cancer cells [45]. CERPI exhibited correlations with the expression of marker genes and the recruitment of diverse immune cell types, encompassing neutrophils, CD4+ T cells, dendritic cells, Th22 cells, Th2 cells, and MDSCs. These immune cell types are notably associated with CRC development and therapeutic outcomes [46–51]. The TME score, IPS, and TIDE were used to evaluate the benefits of immunotherapy in patients with CRC, and low-CERPI patients might benefit more from anti-PD1 or anti-CTLA4 ICI therapy. We further used pan−cancer data to analyze the relevance between CERPI and malignant tumor phenotypes, inducing vasculature, activating invasion and metastasis, and sustaining proliferative signaling, which have been identified as basic hallmarks of cancer [52]. CERPI was positively correlated with angiogenesis, EMT, and cell cycle, indicating that CERPI was significantly related to multiple processes of tumor occurrence and development. To screen biomarkers related to prognosis and immunotherapy efficacy, a combination of machine learning methods was performed using seven immunotherapy cohorts; NaiveBayes was the optimal algorithm with the highest AUC values, thirteen key genes after insertion in the prognostic and immunotherapy−related signature genes. Out of the 13 key genes, ten exhibited significant upregulation or downregulation in CRC tissues when compared to normal tissues, and their expression in different single cell types was further analyzed. Most of the key genes were widely expressed in immune cells. Since HOXC6, G0S2, and MX2 have not been well studied in CRC, qRT−PCR was conducted on normal intestinal epithelial cells and CRC cell lines. Additionally, immunohistochemistry experiments were employed to assess protein expression levels in CRC tissues and adjacent normal tissues. The analysis revealed a significant elevation in the expression of HOXC6 and G0S2 in both CRC cell lines and tissues, while MX2 expression was upregulated specifically in CRC tissues. Notably, MX2 expression exhibited a relatively lower level in CRC cell lines. A previous study [53] indicated that MX2 plays an important role in innate immunity against HIV-1, suggesting that MX2 might produce a marked effect by regulating anti−tumor immunity without directly affecting tumor cells in CRC. Therefore, we performed an in vitro experiment to explore the effects of G0S2 and HOXC6 knockdown on CRC cells and found that their knockdown significantly inhibited the growth and migration of the RKO cell line, suggesting that G0S2 and HOXC6 are potential diagnostic and therapeutic targets for CRC.

This study had some limitations. First, the signature was constructed and validated solely using publicly available datasets, which might have resulted in a selection bias. More clinical in−house cohorts should be applied to verify our findings. Second, additional clinical information, such as tumor markers and surgical information, should be considered. Finally, Further comprehensive in vitro and in vivo experiments are imperative to delve into the molecular functions of the signature genes concerning growth, metastasis, and anti−tumor immunity in CRC.

## Conclusion

Robust machine learning algorithms were applied to calculate the prognostic index based on CERGs, which can effectively predict clinical outcomes, immune landscapes, and immunotherapy responses in patients with CRC. The results can provide new insights in the diagnosis and precise treatment of CRC. The key genes G0S2 and HOXC6 promote the proliferation and migration of CRC cell lines.

### Supplementary Information

**Additional file 1: Figure S1.** (A) Expression and (B) prognostic significance of 31 core CERGs in TCGA-CRC dataset.** Figure S2.** IHC score of HOXC6 (A), G0S2 (B), and MX2 (C) in normal tissues and CRC. **p < 0.01; ***p < 0.001.** Table S1.** Published signatures applied for model comparison.** Table S2.** Sequences for qRT-PCR primers.** Table S3.** Detailed si-RNA sequences used in the study.** Table S4.** 182 CERGs from published research and 1793 IRGs from Immport database.** Table S5.** Published signatures applied for model comparison. C-index of each combination of machine learning method for developing the prognostic signature.** Table S6.** AUC value of each combination of machine learning method for constructing the immunotherapy-related signature.

## Data Availability

Transcription and single cell sequencing data can be found from online repositories. Additional information concerning the organoids supporting the findings of this study is available from the corresponding author upon reasonable request.

## Abbreviations

CERGs: Genes associated with cytotoxic T lymphocyte evasion
CRC: Colorectal cancer
CERPI: CERGs prognostic index
ICI: Immune checkpoint inhibitor
PD-1: Programmed death receptor 1
MSI: Microsatellite instability
CTL: Cytotoxic T lymphocytes
TIDE: Tumor immune dysfunction and exclusion
IRGs: Immune−related genes
CNV: Copy number variation
GO: Gene ontology
KEGG: Kyoto encyclopedia of genes and genomes
PCA: Principal component analysis
ssGSEA: Single−sample gene set enrichment analysis
DEGs: Differentially expressed genes
plsRcox: Partial least squares regression for cox
Lasso: Least absolute shrinkage and selection operator
Enet: Elastic network
survival−SVM: Survival support vector machine
GBM: Generalized boosted regression modeling
OS: Overall survival
TME: Tumor microenvironment
IPS: Immune cell proportion score
CR: Complete response
PR: Partial response
SD: Stable disease
PD: Progressive disease
AUC: Area under curve
DMEM: Dulbecco’s modified Eagle’s medium
FBS: Fetal bovine serum
cDNA: Complementary DNA
MDSCs: Myeloid−derived suppressor cells
EMT: Epithelial−Mesenchymal Transition
CEA: Carcinoembryonic antigen
