# Supplementary material for: Robust machine−learning based prognostic index using cytotoxic T lymphocyte evasion genes highlights potential therapeutic targets in colorectal cancer
Source: Cancer Cell Int. 2024 Jan 31;24:52. doi: 10.1186/s12935-024-03239-y (PMC10829178; doi:10.1186/s12935-024-03239-y)
Supplement: Supplementary file 1 — Additional file 1: Figure S1. (A) Expression and (B) prognostic significance of 31 core CERGs in TCGA-CRC dataset. Figure S2. IHC score of HOXC6 (A), G0S2 (B), and MX2 (C) in normal tissues and CRC. **p < 0.01; ***p < 0.001. Table S1. Published signatures applied for model comparison. Table S2. Sequences for qRT-PCR primers. Table S3. Detailed si-RNA sequences used in the study. Table S4. 182 CERGs from published research and 1793 IRGs from Immport database. Table S5. Published signatures applied for model comparison. C-index of each combination of machine learning method for developing the prognostic signature. Table S6. AUC value of each combination of machine learning method for constructing the immunotherapy-related signature. [file 12935_2024_3239_MOESM1_ESM.zip › Supplementary Material/Supplementary Table S1.docx]

| **Supplementary Table S1: Published signatures applied for model comparison.** | | | | | |
| --- | --- | --- | --- | --- | --- |
| **Model** | **PMID** | **Type** | **Author** | **ENSEMBL** | **Coef** |
| Abdul | 27609023 | mRNA | Abdul | ENSG00000012048 | -0.3567 |
| Abdul | 27609023 | mRNA | Abdul | ENSG00000174206 | 0.1240 |
| Abdul | 27609023 | mRNA | Abdul | ENSG00000147183 | -0.1401 |
| Abdul | 27609023 | mRNA | Abdul | ENSG00000140279 | -0.0424 |
| Abdul | 27609023 | mRNA | Abdul | ENSG00000189037 | -0.2025 |
| Abdul | 27609023 | mRNA | Abdul | ENSG00000139926 | 0.1038 |
| Abdul | 27609023 | mRNA | Abdul | ENSG00000125861 | -0.2956 |
| Abdul | 27609023 | mRNA | Abdul | ENSG00000148841 | 0.1219 |
| Abdul | 27609023 | mRNA | Abdul | ENSG00000172590 | 0.0273 |
| Abdul | 27609023 | mRNA | Abdul | ENSG00000134250 | 0.1748 |
| Abdul | 27609023 | mRNA | Abdul | ENSG00000117859 | -0.2194 |
| Abdul | 27609023 | mRNA | Abdul | ENSG00000116703 | -0.0565 |
| Abdul | 27609023 | mRNA | Abdul | ENSG00000177058 | -0.0005 |
| Abdul | 27609023 | mRNA | Abdul | ENSG00000184985 | -0.3010 |
| Abdul | 27609023 | mRNA | Abdul | ENSG00000197912 | 0.1790 |
| Abdul | 27609023 | mRNA | Abdul | ENSG00000071539 | 0.0291 |
| Abdul | 27609023 | mRNA | Abdul | ENSG00000162618 | 0.1500 |
| Abdul | 27609023 | mRNA | Abdul | ENSG00000172292 | -0.0390 |
| Ahluwalia | 31387239 | mRNA | Ahluwalia | ENSG00000166913 | 1.3244 |
| Ahluwalia | 31387239 | mRNA | Ahluwalia | ENSG00000104738 | 0.9895 |
| Ahluwalia | 31387239 | mRNA | Ahluwalia | ENSG00000108829 | 0.8416 |
| Ahluwalia | 31387239 | mRNA | Ahluwalia | ENSG00000176978 | -0.9676 |
| Alajez | 27935967 | mRNA | Alajez | ENSG00000165617 | 0.0153 |
| Alajez | 27935967 | mRNA | Alajez | ENSG00000197757 | 0.3017 |
| Alajez | 27935967 | mRNA | Alajez | ENSG00000162849 | -0.0126 |
| Alajez | 27935967 | mRNA | Alajez | ENSG00000196754 | 0.1002 |
| Alajez | 27935967 | mRNA | Alajez | ENSG00000146469 | 0.1017 |
| Bai | 32096169 | mRNA | Bai | ENSG00000174564 | 0.3850 |
| Bai | 32096169 | mRNA | Bai | ENSG00000171643 | 0.3630 |
| Bai | 32096169 | mRNA | Bai | ENSG00000225950 | 0.2730 |
| Bai | 32096169 | mRNA | Bai | ENSG00000188710 | 0.2610 |
| Bai | 32096169 | mRNA | Bai | ENSG00000105697 | 0.1530 |
| Bai | 32096169 | mRNA | Bai | ENSG00000115598 | 0.0975 |
| Bai | 32096169 | mRNA | Bai | ENSG00000198049 | 0.0810 |
| Bai | 32096169 | mRNA | Bai | ENSG00000258839 | 0.0500 |
| Bai | 32096169 | mRNA | Bai | ENSG00000106178 | -0.0880 |
| Bai | 32096169 | mRNA | Bai | ENSG00000184009 | -0.1160 |
| Bai | 32096169 | mRNA | Bai | ENSG00000143365 | -0.1320 |
| Bai | 32096169 | mRNA | Bai | ENSG00000104432 | -0.2530 |
| Bai | 32096169 | mRNA | Bai | ENSG00000158485 | -0.3640 |
| Bai | 32096169 | mRNA | Bai | ENSG00000087266 | -0.4290 |
| Bu | 34035992 | mRNA | Bu | ENSG00000205593 | 0.4069 |
| Bu | 34035992 | mRNA | Bu | ENSG00000019186 | 0.2379 |
| Bu | 34035992 | mRNA | Bu | ENSG00000135439 | 0.1485 |
| Bu | 34035992 | mRNA | Bu | ENSG00000113119 | 0.0723 |
| Bu | 34035992 | mRNA | Bu | ENSG00000135218 | 0.0555 |
| Bu | 34035992 | mRNA | Bu | ENSG00000104921 | 0.0484 |
| Bu | 34035992 | mRNA | Bu | ENSG00000105656 | 0.0297 |
| Bu | 34035992 | mRNA | Bu | ENSG00000204310 | 0.0158 |
| Bu | 34035992 | mRNA | Bu | ENSG00000136802 | 0.0119 |
| Bu | 34035992 | mRNA | Bu | ENSG00000115457 | 0.0056 |
| Bu | 34035992 | mRNA | Bu | ENSG00000128564 | 0.0046 |
| Bu | 34035992 | mRNA | Bu | ENSG00000060762 | 0.0003 |
| Bu | 34035992 | mRNA | Bu | ENSG00000146674 | 0.0000 |
| Bu | 34035992 | mRNA | Bu | ENSG00000105329 | -0.0004 |
| Bu | 34035992 | mRNA | Bu | ENSG00000111912 | -0.0149 |
| Bu | 34035992 | mRNA | Bu | ENSG00000104687 | -0.0194 |
| Bu | 34035992 | mRNA | Bu | ENSG00000113916 | -0.0458 |
| Busuioc | 34073426 | mRNA | Busuioc | ENSG00000112175 | -0.2706 |
| Busuioc | 34073426 | mRNA | Busuioc | ENSG00000197380 | 0.1103 |
| Busuioc | 34073426 | mRNA | Busuioc | ENSG00000101210 | 0.1215 |
| Busuioc | 34073426 | mRNA | Busuioc | ENSG00000111846 | -0.0115 |
| Busuioc | 34073426 | mRNA | Busuioc | ENSG00000136231 | 0.0825 |
| Busuioc | 34073426 | mRNA | Busuioc | ENSG00000086991 | 0.0498 |
| Busuioc | 34073426 | mRNA | Busuioc | ENSG00000104332 | -0.0504 |
| Cai | 33714257 | lncRNA | Cai | ENSG00000203993 | 0.3470 |
| Cai | 33714257 | lncRNA | Cai | ENSG00000233901 | 0.2360 |
| Cai | 33714257 | lncRNA | Cai | ENSG00000247556 | 0.1580 |
| Cai | 33714257 | lncRNA | Cai | ENSG00000254810 | 0.5190 |
| Cai | 33714257 | lncRNA | Cai | ENSG00000239213 | 0.4310 |
| Cai | 33714257 | lncRNA | Cai | ENSG00000244151 | 0.4180 |
| Cai | 33714257 | lncRNA | Cai | ENSG00000265206 | 0.1690 |
| Chen-FM | 34211990 | mRNA | Chen-FM | ENSG00000175938 | 0.0520 |
| Chen-FM | 34211990 | mRNA | Chen-FM | ENSG00000100243 | 0.0050 |
| Chen-FM | 34211990 | mRNA | Chen-FM | ENSG00000163840 | -0.0030 |
| Chen-FM | 34211990 | mRNA | Chen-FM | ENSG00000145779 | -0.0060 |
| Chen-FM | 34211990 | mRNA | Chen-FM | ENSG00000138794 | -0.0190 |
| Chen-FM | 34211990 | mRNA | Chen-FM | ENSG00000011258 | -0.0230 |
| Chen-FM | 34211990 | mRNA | Chen-FM | ENSG00000186314 | -0.0230 |
| Chen-FM | 34211990 | mRNA | Chen-FM | ENSG00000196323 | -0.0500 |
| Chen-FM | 34211990 | mRNA | Chen-FM | ENSG00000138439 | -0.0590 |
| Chen-FM | 34211990 | mRNA | Chen-FM | ENSG00000125347 | -0.0700 |
| Chen-FM | 34211990 | mRNA | Chen-FM | ENSG00000107789 | -0.0790 |
| Chen-FM | 34211990 | mRNA | Chen-FM | ENSG00000131323 | -0.0870 |
| Chen-Gene | 30654001 | mRNA | Chen-Gene | ENSG00000102265 | 0.3424 |
| Chen-Gene | 30654001 | mRNA | Chen-Gene | ENSG00000186417 | 0.2082 |
| Chen-Gene | 30654001 | mRNA | Chen-Gene | ENSG00000151882 | 0.1149 |
| Chen-Gene | 30654001 | mRNA | Chen-Gene | ENSG00000163710 | 0.0834 |
| Chen-Gene | 30654001 | mRNA | Chen-Gene | ENSG00000124939 | -0.0991 |
| Chen-Gene | 30654001 | mRNA | Chen-Gene | ENSG00000080493 | -0.1068 |
| Chen-Gene | 30654001 | mRNA | Chen-Gene | ENSG00000168646 | -0.1080 |
| Chen-Gene | 30654001 | mRNA | Chen-Gene | ENSG00000196611 | -0.1516 |
| Chen-Gene | 30654001 | mRNA | Chen-Gene | ENSG00000050344 | -0.2564 |
| Chen-ONCO | 29221110 | mRNA | Chen-ONCO | ENSG00000127329 | -0.0080 |
| Chen-ONCO | 29221110 | mRNA | Chen-ONCO | ENSG00000188811 | -0.1330 |
| Chen-ONCO | 29221110 | mRNA | Chen-ONCO | ENSG00000108439 | -0.7150 |
| Chen-ONCO | 29221110 | mRNA | Chen-ONCO | ENSG00000175893 | -0.7810 |
| Chen-ONCO | 29221110 | mRNA | Chen-ONCO | ENSG00000145725 | -1.0040 |
| Chen-ONCO | 29221110 | mRNA | Chen-ONCO | ENSG00000183530 | -1.0770 |
| Chen-ONCO | 29221110 | mRNA | Chen-ONCO | ENSG00000171097 | 0.8230 |
| Chen-OTT | 34103941 | mRNA | Chen-OTT | ENSG00000106483 | 0.0354 |
| Chen-OTT | 34103941 | mRNA | Chen-OTT | ENSG00000149591 | 0.0194 |
| Chen-OTT | 34103941 | mRNA | Chen-OTT | ENSG00000079308 | 0.0073 |
| Cheng-CMR | 30214289 | mRNA | Cheng-CMR | ENSG00000115414 | 0.0205 |
| Cheng-CMR | 30214289 | mRNA | Cheng-CMR | ENSG00000106366 | 0.1106 |
| Cheng-CMR | 30214289 | mRNA | Cheng-CMR | ENSG00000186340 | 0.0228 |
| Cheng-IJMS | 33390777 | lncRNA | Cheng-IJMS | ENSG00000232065 | 0.3811 |
| Cheng-IJMS | 33390777 | lncRNA | Cheng-IJMS | ENSG00000247095 | 0.2088 |
| Cheng-IJMS | 33390777 | lncRNA | Cheng-IJMS | ENSG00000264247 | 0.1463 |
| Cheng-IJMS | 33390777 | lncRNA | Cheng-IJMS | ENSG00000233223 | 0.6290 |
| Cheng-IJMS | 33390777 | lncRNA | Cheng-IJMS | ENSG00000272913 | 0.2212 |
| Cheng-IJMS | 33390777 | lncRNA | Cheng-IJMS | ENSG00000257883 | 0.1463 |
| Chu | 32626715 | lncRNA | Chu | ENSG00000152034 | 17.1340 |
| Chu | 32626715 | lncRNA | Chu | ENSG00000147883 | -0.9350 |
| Chu | 32626715 | lncRNA | Chu | ENSG00000273733 | 1.6790 |
| Chu | 32626715 | lncRNA | Chu | ENSG00000239828 | -36.2720 |
| ColoFinder | 26989635 | mRNA | ColoFinder | ENSG00000134982 | NA |
| ColoFinder | 26989635 | mRNA | ColoFinder | ENSG00000133703 | NA |
| ColoFinder | 26989635 | mRNA | ColoFinder | ENSG00000076242 | NA |
| ColoFinder | 26989635 | mRNA | ColoFinder | ENSG00000095002 | NA |
| ColoFinder | 26989635 | mRNA | ColoFinder | ENSG00000116062 | NA |
| ColoFinder | 26989635 | mRNA | ColoFinder | ENSG00000171862 | NA |
| ColoFinder | 26989635 | mRNA | ColoFinder | ENSG00000141646 | NA |
| ColoFinder | 26989635 | mRNA | ColoFinder | ENSG00000163513 | NA |
| ColoFinder | 26989635 | mRNA | ColoFinder | ENSG00000141510 | NA |
| ColoGuidePro | 22991413 | mRNA | ColoGuidePro | ENSG00000075213 | 0.1000 |
| ColoGuidePro | 22991413 | mRNA | ColoGuidePro | ENSG00000085741 | 0.1000 |
| ColoGuidePro | 22991413 | mRNA | ColoGuidePro | ENSG00000135318 | 0.0500 |
| ColoGuidePro | 22991413 | mRNA | ColoGuidePro | ENSG00000187908 | 0.0400 |
| ColoGuidePro | 22991413 | mRNA | ColoGuidePro | ENSG00000197888 | -0.0200 |
| ColoGuidePro | 22991413 | mRNA | ColoGuidePro | ENSG00000102837 | -0.0800 |
| ColoGuidePro | 22991413 | mRNA | ColoGuidePro | ENSG00000138755 | -0.1000 |
| ColoLipidGene | 25749516 | mRNA | ColoLipidGene | ENSG00000165029 | -0.0263 |
| ColoLipidGene | 25749516 | mRNA | ColoLipidGene | ENSG00000151726 | 0.0047 |
| ColoLipidGene | 25749516 | mRNA | ColoLipidGene | ENSG00000204310 | 0.1913 |
| ColoLipidGene | 25749516 | mRNA | ColoLipidGene | ENSG00000099194 | 0.0621 |
| Dai-FIG | 33343640 | mRNA | Dai-FIG | ENSG00000167191 | 0.6130 |
| Dai-FIG | 33343640 | mRNA | Dai-FIG | ENSG00000137312 | 0.5990 |
| Dai-FIG | 33343640 | mRNA | Dai-FIG | ENSG00000174564 | 0.5960 |
| Dai-FIG | 33343640 | mRNA | Dai-FIG | ENSG00000106683 | 0.4020 |
| Dai-FIG | 33343640 | mRNA | Dai-FIG | ENSG00000102962 | -0.4210 |
| Dai-FIG | 33343640 | mRNA | Dai-FIG | ENSG00000114738 | -0.4650 |
| Dai-MO | 29377588 | mRNA | Dai-MO | ENSG00000143369 | 0.2590 |
| Dai-MO | 29377588 | mRNA | Dai-MO | ENSG00000129451 | 0.2010 |
| Dai-MO | 29377588 | mRNA | Dai-MO | ENSG00000100453 | 0.0430 |
| Dai-MO | 29377588 | mRNA | Dai-MO | ENSG00000102854 | 0.0380 |
| Dai-MO | 29377588 | mRNA | Dai-MO | ENSG00000205420 | -0.0150 |
| Dai-MO | 29377588 | mRNA | Dai-MO | ENSG00000115009 | -0.0470 |
| Dai-MO | 29377588 | mRNA | Dai-MO | ENSG00000133627 | -0.0520 |
| Dai-MO | 29377588 | mRNA | Dai-MO | ENSG00000104432 | -0.1020 |
| Dai-MO | 29377588 | mRNA | Dai-MO | ENSG00000108578 | -0.1160 |
| Dai-MO | 29377588 | mRNA | Dai-MO | ENSG00000134326 | -0.1210 |
| Dai-MO | 29377588 | mRNA | Dai-MO | ENSG00000130818 | -0.1680 |
| Dai-MO | 29377588 | mRNA | Dai-MO | ENSG00000089127 | -0.2170 |
| Dai-MO | 29377588 | mRNA | Dai-MO | ENSG00000091127 | -0.2360 |
| Dai-MO | 29377588 | mRNA | Dai-MO | ENSG00000144485 | -0.2870 |
| Dai-MO | 29377588 | mRNA | Dai-MO | ENSG00000100985 | -0.3020 |
| Dekervel | 24486594 | mRNA | Dekervel | ENSG00000099860 | 0.5960 |
| Dekervel | 24486594 | mRNA | Dekervel | ENSG00000107949 | 0.5430 |
| Dekervel | 24486594 | mRNA | Dekervel | ENSG00000125629 | 0.5380 |
| Dekervel | 24486594 | mRNA | Dekervel | ENSG00000141510 | -0.1770 |
| Dekervel | 24486594 | mRNA | Dekervel | ENSG00000104765 | -0.4160 |
| Dekervel | 24486594 | mRNA | Dekervel | ENSG00000135698 | -1.0000 |
| Dong | 30655785 | mRNA | Dong | ENSG00000081479 | 0.2530 |
| Dong | 30655785 | mRNA | Dong | ENSG00000138795 | 0.1390 |
| Dong | 30655785 | mRNA | Dong | ENSG00000146674 | 0.0840 |
| Dong | 30655785 | mRNA | Dong | ENSG00000163909 | 0.0620 |
| Dong | 30655785 | mRNA | Dong | ENSG00000099860 | 0.0580 |
| Dong | 30655785 | mRNA | Dong | ENSG00000107611 | 0.0480 |
| Dong | 30655785 | mRNA | Dong | ENSG00000145423 | 0.0360 |
| Dong | 30655785 | mRNA | Dong | ENSG00000105173 | -0.1900 |
| Fan | 29227531 | lncRNA | Fan | ENSG00000259347 | 0.8131 |
| Fan | 29227531 | lncRNA | Fan | ENSG00000517726 | 0.1337 |
| Fan | 29227531 | lncRNA | Fan | ENSG00000253405 | 0.0633 |
| Fu | 33203797 | mRNA | Fu | ENSG00000152192 | 1.2040 |
| Fu | 33203797 | mRNA | Fu | ENSG00000198681 | 1.1277 |
| Fu | 33203797 | mRNA | Fu | ENSG00000139910 | 1.1272 |
| Fu | 33203797 | mRNA | Fu | ENSG00000161652 | 0.8646 |
| Fu | 33203797 | mRNA | Fu | ENSG00000173930 | 0.8003 |
| Ge | 30881123 | mRNA | Ge | ENSG00000121989 | 0.0515 |
| Ge | 30881123 | mRNA | Ge | ENSG00000134982 | -0.0908 |
| Ge | 30881123 | mRNA | Ge | ENSG00000134516 | -0.0069 |
| Ge | 30881123 | mRNA | Ge | ENSG00000177084 | 0.0090 |
| Gharib | 32155177 | mRNA | Gharib | ENSG00000101473 | 0.6590 |
| Gharib | 32155177 | mRNA | Gharib | ENSG00000134240 | 0.2010 |
| Gharib | 32155177 | mRNA | Gharib | ENSG00000197142 | 0.0840 |
| Gharib | 32155177 | mRNA | Gharib | ENSG00000169710 | 0.0290 |
| Gharib | 32155177 | mRNA | Gharib | ENSG00000099194 | 0.1190 |
| Giráldez | 22833293 | mRNA | Giráldez | ENSG00000196754 | 0.7106 |
| Giráldez | 22833293 | mRNA | Giráldez | ENSG00000197747 | 0.5291 |
| Giráldez | 22833293 | mRNA | Giráldez | ENSG00000197747 | 0.5291 |
| Goeman | 33377637 | mRNA | Goeman | ENSG00000134982 | 0.0195 |
| Goeman | 33377637 | mRNA | Goeman | ENSG00000113712 | -0.2915 |
| Goeman | 33377637 | mRNA | Goeman | ENSG00000107984 | 0.0358 |
| Goeman | 33377637 | mRNA | Goeman | ENSG00000139174 | 0.1041 |
| Goeman | 33377637 | mRNA | Goeman | ENSG00000104332 | -0.1236 |
| Goeman | 33377637 | mRNA | Goeman | ENSG00000145423 | 0.2797 |
| Goeman | 33377637 | mRNA | Goeman | ENSG00000164736 | 0.0123 |
| Gong | 32351322 | mRNA | Gong | ENSG00000183571 | 0.0676 |
| Gong | 32351322 | mRNA | Gong | ENSG00000125414 | 0.0648 |
| Gong | 32351322 | mRNA | Gong | ENSG00000175877 | 0.0120 |
| Gong | 32351322 | mRNA | Gong | ENSG00000152192 | 0.0042 |
| Gong | 32351322 | mRNA | Gong | ENSG00000100625 | 0.0031 |
| Gong | 32351322 | mRNA | Gong | ENSG00000196260 | 0.0012 |
| Gong | 32351322 | mRNA | Gong | ENSG00000104899 | 0.0008 |
| Gong | 32351322 | mRNA | Gong | ENSG00000196092 | 0.0008 |
| Gu | 29749517 | lncRNA | Gu | ENSG00000260267 | 0.3844 |
| Gu | 29749517 | lncRNA | Gu | ENSG00000261040 | -0.2715 |
| Gu | 29749517 | lncRNA | Gu | ENSG00000253417 | -0.2810 |
| Gu | 29749517 | lncRNA | Gu | ENSG00000255964 | -0.4740 |
| Gu | 29749517 | lncRNA | Gu | ENSG00000246451 | -0.6862 |
| Hao | 20077526 | mRNA | Hao | ENSG00000107984 | -0.0106 |
| Hao | 20077526 | mRNA | Hao | ENSG00000254087 | -0.1455 |
| Hao | 20077526 | mRNA | Hao | ENSG00000071054 | 0.1153 |
| Hao | 20077526 | mRNA | Hao | ENSG00000101871 | -0.1273 |
| Hao | 20077526 | mRNA | Hao | ENSG00000137575 | 0.0310 |
| He | 34193169 | mRNA | He | ENSG00000140986 | 2.6420 |
| He | 34193169 | mRNA | He | ENSG00000110888 | 0.1942 |
| He | 34193169 | mRNA | He | ENSG00000158941 | -0.0616 |
| He | 34193169 | mRNA | He | ENSG00000103342 | -0.0658 |
| He | 34193169 | mRNA | He | ENSG00000163319 | -0.4546 |
| Hu | 24809982 | lncRNA | Hu | ENSG00000276012 | 0.2433 |
| Hu | 24809982 | lncRNA | Hu | ENST00000425086 | 0.0783 |
| Hu | 24809982 | lncRNA | Hu | ENSG00000235527 | -0.0017 |
| Hu | 24809982 | lncRNA | Hu | ENSG00000235527 | -0.1430 |
| Hu | 24809982 | lncRNA | Hu | CR622106 | -0.1936 |
| Hu | 24809982 | lncRNA | Hu | ENSG00000235527 | -0.2086 |
| Huang | 34178621 | mRNA | Huang | ENSG00000156127 | 0.2530 |
| Huang | 34178621 | mRNA | Huang | ENSG00000165443 | 0.1470 |
| Huang | 34178621 | mRNA | Huang | ENSG00000114115 | -0.1720 |
| Huang | 34178621 | mRNA | Huang | ENSG00000006757 | -0.1830 |
| Huang-ACP | 33833937 | mRNA | Huang-ACP | ENSG00000100628 | 0.3082 |
| Huang-ACP | 33833937 | mRNA | Huang-ACP | ENSG00000135406 | 0.0701 |
| Huang-ACP | 33833937 | mRNA | Huang-ACP | ENSG00000154165 | -0.0651 |
| Huang-ACP | 33833937 | mRNA | Huang-ACP | ENSG00000165799 | -0.1112 |
| Huang-ACP | 33833937 | mRNA | Huang-ACP | ENSG00000100721 | -0.1428 |
| Huang-BR | 32128213 | lncRNA | Huang-BR | ENSG00000261780 | 1.7500 |
| Huang-BR | 32128213 | lncRNA | Huang-BR | ENSG00000237187 | 1.4900 |
| Huang-BR | 32128213 | lncRNA | Huang-BR | ENSG00000225187 | 1.1800 |
| Huang-BR | 32128213 | lncRNA | Huang-BR | ENSG00000171889 | 0.7200 |
| Huang-BR | 32128213 | lncRNA | Huang-BR | ENSG00000225746 | 0.6700 |
| Huang-BR | 32128213 | lncRNA | Huang-BR | ENSG00000250742 | 0.5700 |
| Huang-BR | 32128213 | lncRNA | Huang-BR | ENSG00000267242 | -1.7500 |
| Huang-DO | 31853237 | mRNA | Huang-DO | ENSG00000112715 | 0.3215 |
| Huang-DO | 31853237 | mRNA | Huang-DO | ENSG00000147889 | 0.2031 |
| Huang-DO | 31853237 | mRNA | Huang-DO | ENSG00000157168 | -1.2119 |
| Ji | 32500031 | mRNA | Ji | ENSG00000091542 | 0.5710 |
| Ji | 32500031 | mRNA | Ji | ENSG00000047188 | -0.4155 |
| Jiang | 33377636 | mRNA | Jiang | ENSG00000184012 | 0.3193 |
| Jiang | 33377636 | mRNA | Jiang | ENSG00000172005 | 0.2244 |
| Jiang | 33377636 | mRNA | Jiang | ENSG00000070882 | 0.1450 |
| Jiang | 33377636 | mRNA | Jiang | ENSG00000171951 | 0.1303 |
| Jiang | 33377636 | mRNA | Jiang | ENSG00000118785 | 0.0853 |
| Jiang | 33377636 | mRNA | Jiang | ENSG00000204936 | 0.0253 |
| Jiang | 33377636 | mRNA | Jiang | ENSG00000156802 | -0.1824 |
| Jiang | 33377636 | mRNA | Jiang | ENSG00000141401 | -0.1933 |
| Jiang | 33377636 | mRNA | Jiang | ENSG00000197892 | -0.2652 |
| Jiang | 33377636 | mRNA | Jiang | ENSG00000156006 | -0.2847 |
| Jiang | 33377636 | mRNA | Jiang | ENSG00000162390 | -0.3018 |
| Jiang | 33377636 | mRNA | Jiang | ENSG00000006555 | -0.3122 |
| Jiang | 33377636 | mRNA | Jiang | ENSG00000188373 | 0.3791 |
| Jiang | 33377636 | mRNA | Jiang | ENSG00000176485 | 0.1914 |
| Jiang | 33377636 | mRNA | Jiang | ENSG00000152763 | -0.3017 |
| Ke | 32863096 | mRNA | Ke | ENSG00000112715 | 0.3000 |
| Ke | 32863096 | mRNA | Ke | ENSG00000005381 | 0.1890 |
| Ke | 32863096 | mRNA | Ke | ENSG00000160691 | 0.1710 |
| Ke | 32863096 | mRNA | Ke | ENSG00000122133 | 0.1640 |
| Ke | 32863096 | mRNA | Ke | ENSG00000082175 | 0.1570 |
| Ke | 32863096 | mRNA | Ke | ENSG00000198753 | 0.1540 |
| Ke | 32863096 | mRNA | Ke | ENSG00000166333 | 0.1110 |
| Ke | 32863096 | mRNA | Ke | ENSG00000145826 | 0.0770 |
| Ke | 32863096 | mRNA | Ke | ENSG00000188015 | 0.0680 |
| Ke | 32863096 | mRNA | Ke | ENSG00000109072 | 0.0680 |
| Ke | 32863096 | mRNA | Ke | ENSG00000080293 | 0.0630 |
| Ke | 32863096 | mRNA | Ke | ENSG00000153993 | 0.0580 |
| Ke | 32863096 | mRNA | Ke | ENSG00000269335 | 0.0330 |
| Ke | 32863096 | mRNA | Ke | ENSG00000101074 | 0.0310 |
| Ke | 32863096 | mRNA | Ke | ENSG00000113389 | 0.0210 |
| Ke | 32863096 | mRNA | Ke | ENSG00000122861 | 0.0050 |
| Ke | 32863096 | mRNA | Ke | ENSG00000164520 | 0.0000 |
| Ke | 32863096 | mRNA | Ke | ENSG00000156234 | -0.0660 |
| Ke | 32863096 | mRNA | Ke | ENSG00000139574 | -0.0690 |
| Ke | 32863096 | mRNA | Ke | ENSG00000168229 | -0.1460 |
| Ke | 32863096 | mRNA | Ke | ENSG00000131323 | -0.1640 |
| Ke | 32863096 | mRNA | Ke | ENSG00000125257 | -0.2670 |
| Ke | 32863096 | mRNA | Ke | ENSG00000001617 | -0.3550 |
| Kim | 31578316 | mRNA | Kim | ENSG00000166848 | 0.8870 |
| Kim | 31578316 | mRNA | Kim | ENSG00000150093 | 0.7580 |
| Kim | 31578316 | mRNA | Kim | ENSG00000156976 | 0.5430 |
| Kim | 31578316 | mRNA | Kim | ENSG00000071054 | 0.5390 |
| Kim | 31578316 | mRNA | Kim | ENSG00000155366 | 0.3300 |
| Kim | 31578316 | mRNA | Kim | ENSG00000106211 | 0.3190 |
| Kim | 31578316 | mRNA | Kim | ENSG00000262406 | -0.1330 |
| Kim | 31578316 | mRNA | Kim | ENSG00000164045 | -0.3630 |
| Kim | 31578316 | mRNA | Kim | ENSG00000015475 | -0.5040 |
| Kim | 31578316 | mRNA | Kim | ENSG00000004455 | -0.5360 |
| Kim | 31578316 | mRNA | Kim | ENSG00000110958 | -0.6960 |
| Li-CMS | 32494194 | mRNA | Li-CMS | ENSG00000150093 | 0.6432 |
| Li-CMS | 32494194 | mRNA | Li-CMS | ENSG00000100625 | 0.3413 |
| Li-CMS | 32494194 | mRNA | Li-CMS | ENSG00000121879 | 0.2717 |
| Li-CMS | 32494194 | mRNA | Li-CMS | ENSG00000161638 | 0.1874 |
| Li-CMS | 32494194 | mRNA | Li-CMS | ENSG00000196338 | 0.1382 |
| Li-CMS | 32494194 | mRNA | Li-CMS | ENSG00000141668 | 0.1270 |
| Li-CMS | 32494194 | mRNA | Li-CMS | ENSG00000107984 | 0.0732 |
| Li-CMS | 32494194 | mRNA | Li-CMS | ENSG00000163735 | -0.0288 |
| Li-CMS | 32494194 | mRNA | Li-CMS | ENSG00000114251 | -0.0768 |
| Li-CMS | 32494194 | mRNA | Li-CMS | ENSG00000081041 | -0.1207 |
| Li-CMS | 32494194 | mRNA | Li-CMS | ENSG00000163739 | -0.1391 |
| Li-CMS | 32494194 | mRNA | Li-CMS | ENSG00000182580 | -0.1591 |
| Li-CMS | 32494194 | mRNA | Li-CMS | ENSG00000144771 | -0.2820 |
| Li-CMS | 32494194 | mRNA | Li-CMS | ENSG00000133216 | -0.3110 |
| Li-CMS | 32494194 | mRNA | Li-CMS | ENSG00000141378 | -0.4696 |
| Li-FIG | 34017356 | mRNA | Li-FIG | ENSG00000128342 | 0.7660 |
| Li-FIG | 34017356 | mRNA | Li-FIG | ENSG00000110944 | 0.5010 |
| Li-FIG | 34017356 | mRNA | Li-FIG | ENSG00000128564 | 0.3840 |
| Li-FIG | 34017356 | mRNA | Li-FIG | ENSG00000156427 | 0.3510 |
| Li-FIG | 34017356 | mRNA | Li-FIG | ENSG00000145147 | 0.1790 |
| Li-FIG | 34017356 | mRNA | Li-FIG | ENSG00000151882 | -0.1900 |
| Li-FM | 32211413 | lncRNA | Li-FM | ENSG00000228630 | 0.1507 |
| Li-FM | 32211413 | lncRNA | Li-FM | ENSG00000205293 | 0.1177 |
| Li-FM | 32211413 | lncRNA | Li-FM | ENSG00000278910 | 0.1057 |
| Li-FM | 32211413 | lncRNA | Li-FM | ENSG00000261175 | 0.0173 |
| Li-FM | 32211413 | lncRNA | Li-FM | ENSG00000229331 | -0.1327 |
| Li-FM | 32211413 | lncRNA | Li-FM | ENSG00000237438 | -0.1419 |
| Li-IntI | 32771948 | mRNA | Li-IntI | ENSG00000163794 | 0.5036 |
| Li-IntI | 32771948 | mRNA | Li-IntI | ENSG00000159167 | 0.2063 |
| Li-IntI | 32771948 | mRNA | Li-IntI | ENSG00000160801 | 0.1689 |
| Li-IntI | 32771948 | mRNA | Li-IntI | ENSG00000163083 | 0.1624 |
| Li-IntI | 32771948 | mRNA | Li-IntI | ENSG00000171951 | 0.0936 |
| Li-IntI | 32771948 | mRNA | Li-IntI | ENSG00000131015 | 0.0906 |
| Li-IntI | 32771948 | mRNA | Li-IntI | ENSG00000105697 | 0.0124 |
| Li-IntI | 32771948 | mRNA | Li-IntI | ENSG00000130513 | -0.0062 |
| Li-IntI | 32771948 | mRNA | Li-IntI | ENSG00000175426 | -0.0242 |
| Li-IntI | 32771948 | mRNA | Li-IntI | ENSG00000151623 | -0.0602 |
| Li-IntI | 32771948 | mRNA | Li-IntI | ENSG00000115008 | -0.0618 |
| Li-IntI | 32771948 | mRNA | Li-IntI | ENSG00000042832 | -0.0702 |
| Li-IntI | 32771948 | mRNA | Li-IntI | ENSG00000178473 | -0.0788 |
| Li-IntI | 32771948 | mRNA | Li-IntI | ENSG00000146858 | -0.1021 |
| Li-IntI | 32771948 | mRNA | Li-IntI | ENSG00000065325 | -0.1176 |
| Li-IntI | 32771948 | mRNA | Li-IntI | ENSG00000229314 | -0.1196 |
| Li-JCA | 32127941 | mRNA | Li-JCA | ENSG00000204140 | 0.2120 |
| Li-JCA | 32127941 | mRNA | Li-JCA | ENSG00000070886 | 0.0790 |
| Li-JCA | 32127941 | mRNA | Li-JCA | ENSG00000011677 | -0.0770 |
| Li-JCA | 32127941 | mRNA | Li-JCA | ENSG00000135443 | -0.3770 |
| Li-LS | 32888940 | mRNA | Li-LS | ENSG00000197272 | 1.7778 |
| Li-LS | 32888940 | mRNA | Li-LS | ENSG00000177455 | 0.3649 |
| Li-LS | 32888940 | mRNA | Li-LS | ENSG00000204381 | 0.2720 |
| Li-LS | 32888940 | mRNA | Li-LS | ENSG00000174564 | 0.0798 |
| Li-LS | 32888940 | mRNA | Li-LS | ENSG00000127863 | 0.0642 |
| Li-LS | 32888940 | mRNA | Li-LS | ENSG00000132463 | -0.0483 |
| Li-LS | 32888940 | mRNA | Li-LS | ENSG00000102962 | -0.5739 |
| Li-TCRT | 34080453 | mRNA | Li-TCRT | ENSG00000101489 | 1.1488 |
| Li-TCRT | 34080453 | mRNA | Li-TCRT | ENSG00000102021 | 0.7699 |
| Li-TCRT | 34080453 | mRNA | Li-TCRT | ENSG00000146453 | 0.6121 |
| Li-TCRT | 34080453 | mRNA | Li-TCRT | ENSG00000164362 | 0.5093 |
| Li-TCRT | 34080453 | mRNA | Li-TCRT | ENSG00000109819 | -0.7152 |
| Li-TCRT | 34080453 | mRNA | Li-TCRT | ENSG00000196116 | -0.7990 |
| Li-TCRT | 34080453 | mRNA | Li-TCRT | ENSG00000012048 | -0.8181 |
| Li-TCRT | 34080453 | mRNA | Li-TCRT | ENSG00000149289 | -0.8421 |
| Liang | 34169901 | mRNA | Liang | ENSG00000136231 | 0.1314 |
| Liang | 34169901 | mRNA | Liang | ENSG00000101104 | 0.1171 |
| Liang | 34169901 | mRNA | Liang | ENSG00000109819 | -0.2100 |
| Liang | 34169901 | mRNA | Liang | ENSG00000187024 | 0.2094 |
| Liang | 34169901 | mRNA | Liang | ENSG00000196116 | -0.2351 |
| Lin-FIO | 34026619 | mRNA | Lin-FIO | ENSG00000170017 | 0.8160 |
| Lin-FIO | 34026619 | mRNA | Lin-FIO | ENSG00000137752 | -0.3230 |
| Lin-FIO | 34026619 | mRNA | Lin-FIO | ENSG00000114737 | -2.2300 |
| Lin-FIO | 34026619 | mRNA | Lin-FIO | ENSG00000012124 | -2.8890 |
| Liu-CEM | 33674956 | mRNA | Liu-CEM | ENSG00000137857 | 0.4796 |
| Liu-CEM | 33674956 | mRNA | Liu-CEM | ENSG00000069696 | 0.3747 |
| Liu-CEM | 33674956 | mRNA | Liu-CEM | ENSG00000179477 | 0.2338 |
| Liu-CEM | 33674956 | mRNA | Liu-CEM | ENSG00000140044 | 0.1205 |
| Liu-CEM | 33674956 | mRNA | Liu-CEM | ENSG00000087510 | 0.1149 |
| Liu-CEM | 33674956 | mRNA | Liu-CEM | ENSG00000175224 | 0.0462 |
| Liu-CEM | 33674956 | mRNA | Liu-CEM | ENSG00000105974 | 0.0100 |
| Liu-CEM | 33674956 | mRNA | Liu-CEM | ENSG00000167676 | 0.0091 |
| Liu-CEM | 33674956 | mRNA | Liu-CEM | ENSG00000079459 | -0.0137 |
| Liu-CEM | 33674956 | mRNA | Liu-CEM | ENSG00000007171 | -0.0206 |
| Liu-FIMB-1 | 33898515 | lncRNA | Liu-FIMB-1 | ENSG00000233901 | 0.3590 |
| Liu-FIMB-1 | 33898515 | lncRNA | Liu-FIMB-1 | ENSG00000229891 | -0.2340 |
| Liu-FIMB-1 | 33898515 | lncRNA | Liu-FIMB-1 | ENSG00000268388 | -0.4110 |
| Liu-FIMB-1 | 33898515 | lncRNA | Liu-FIMB-1 | ENSG00000261971 | 0.5430 |
| Liu-FIMB-1 | 33898515 | lncRNA | Liu-FIMB-1 | ENSG00000225315 | 0.3330 |
| Liu-FIMB-1 | 33898515 | lncRNA | Liu-FIMB-1 | ENSG00000265791 | 0.0270 |
| Liu-FIMB-2 | 33898515 | lncRNA | Liu-FIMB-2 | ENSG00000233901 | 0.2450 |
| Liu-FIMB-2 | 33898515 | lncRNA | Liu-FIMB-2 | ENSG00000261971 | 0.5720 |
| Liu-FIMB-2 | 33898515 | lncRNA | Liu-FIMB-2 | ENSG00000274051 | 0.3070 |
| Liu-FIMB-2 | 33898515 | lncRNA | Liu-FIMB-2 | ENSG00000225315 | 0.3010 |
| Liu-FIMB-2 | 33898515 | lncRNA | Liu-FIMB-2 | ENSG00000281406 | 0.1160 |
| Liu-FIMB-2 | 33898515 | lncRNA | Liu-FIMB-2 | ENSG00000272502 | 0.0620 |
| Liu-FIMB-2 | 33898515 | lncRNA | Liu-FIMB-2 | ENSG00000204876 | -0.0660 |
| Liu-FIMB-2 | 33898515 | lncRNA | Liu-FIMB-2 | ENSG00000234072 | -0.2080 |
| Liu-FIMB-2 | 33898515 | lncRNA | Liu-FIMB-2 | ENSG00000219023 | -0.3510 |
| Liu-FIMB-2 | 33898515 | lncRNA | Liu-FIMB-2 | ENSG00000260920 | -0.9050 |
| Liu-FIO | 31824849 | lncRNA | Liu-FIO | ENSG00000255191 | 0.2212 |
| Liu-FIO | 31824849 | lncRNA | Liu-FIO | ENSG00000254815 | 0.2081 |
| Liu-FIO | 31824849 | lncRNA | Liu-FIO | ENSG00000205293 | 0.1214 |
| Liu-IJMS | 33302562 | lncRNA | Liu-IJMS | ENSG00000228630 | 0.1184 |
| Liu-IJMS | 33302562 | lncRNA | Liu-IJMS | ENSG00000259974 | -0.1477 |
| Liu-IJMS | 33302562 | lncRNA | Liu-IJMS | ENSG00000223806 | -0.2257 |
| Liu-JCB | 30756409 | mRNA | Liu-JCB | ENSG00000079308 | 0.2666 |
| Liu-JCB | 30756409 | mRNA | Liu-JCB | ENSG00000120885 | 0.1855 |
| Liu-JCB | 30756409 | mRNA | Liu-JCB | ENSG00000124882 | -0.1042 |
| Liu-JCB | 30756409 | mRNA | Liu-JCB | ENSG00000269404 | -0.1063 |
| Liu-JCB | 30756409 | mRNA | Liu-JCB | ENSG00000123560 | -0.1364 |
| Liu-JTM | 33413474 | mRNA | Liu-JTM | ENSG00000171136 | 0.4530 |
| Liu-JTM | 33413474 | mRNA | Liu-JTM | ENSG00000268940 | 0.2030 |
| Liu-JTM | 33413474 | mRNA | Liu-JTM | ENSG00000128564 | 0.1650 |
| Lu | 33628243 | mRNA | Lu | ENSG00000171223 | 0.2683 |
| Lu | 33628243 | mRNA | Lu | ENSG00000173801 | 0.2609 |
| Lu | 33628243 | mRNA | Lu | ENSG00000168995 | 0.1748 |
| Lu | 33628243 | mRNA | Lu | ENSG00000100106 | 0.1740 |
| Lu | 33628243 | mRNA | Lu | ENSG00000164283 | 0.1582 |
| Lu | 33628243 | mRNA | Lu | ENSG00000122367 | 0.1461 |
| Lu | 33628243 | mRNA | Lu | ENSG00000173578 | 0.0731 |
| Lu | 33628243 | mRNA | Lu | ENSG00000111886 | 0.0560 |
| Lu | 33628243 | mRNA | Lu | ENSG00000103145 | 0.0293 |
| Lu | 33628243 | mRNA | Lu | ENSG00000169903 | 0.0212 |
| Lu | 33628243 | mRNA | Lu | ENSG00000140307 | -0.0137 |
| Lu | 33628243 | mRNA | Lu | ENSG00000093000 | -0.0243 |
| Lu | 33628243 | mRNA | Lu | ENSG00000148832 | -0.0275 |
| Lu | 33628243 | mRNA | Lu | ENSG00000164902 | -0.0280 |
| Lu | 33628243 | mRNA | Lu | ENSG00000101557 | -0.0335 |
| Lu | 33628243 | mRNA | Lu | ENSG00000107949 | -0.0336 |
| Lu | 33628243 | mRNA | Lu | ENSG00000143748 | -0.0407 |
| Lu | 33628243 | mRNA | Lu | ENSG00000197771 | -0.1087 |
| Lu | 33628243 | mRNA | Lu | ENSG00000101544 | -0.1739 |
| Lu | 33628243 | mRNA | Lu | ENSG00000254470 | -0.3134 |
| Lu | 33628243 | mRNA | Lu | ENSG00000137073 | -0.4162 |
| Lu | 33628243 | mRNA | Lu | ENSG00000188163 | -0.5606 |
| Lu | 33628243 | mRNA | Lu | FLJ90680 | 0.0112 |
| Lu | 33628243 | mRNA | Lu | ENSG00000183508 | -0.0291 |
| Luo | 33282950 | mRNA | Luo | ENSG00000213366 | 0.5690 |
| Luo | 33282950 | mRNA | Luo | ENSG00000115361 | 0.3266 |
| Luo | 33282950 | mRNA | Luo | ENSG00000160282 | 0.3138 |
| Luo | 33282950 | mRNA | Luo | ENSG00000123360 | 0.2605 |
| Luo | 33282950 | mRNA | Luo | ENSG00000197943 | 0.2519 |
| Luo | 33282950 | mRNA | Luo | ENSG00000134201 | 0.2062 |
| Luo | 33282950 | mRNA | Luo | ENSG00000136267 | 0.1908 |
| Luo | 33282950 | mRNA | Luo | ENSG00000133256 | 0.1502 |
| Luo | 33282950 | mRNA | Luo | ENSG00000151224 | 0.1302 |
| Luo | 33282950 | mRNA | Luo | ENSG00000169169 | 0.1239 |
| Luo | 33282950 | mRNA | Luo | ENSG00000163624 | -0.0706 |
| Luo | 33282950 | mRNA | Luo | ENSG00000116771 | -0.0955 |
| Luo | 33282950 | mRNA | Luo | ENSG00000136872 | -0.1118 |
| Luo | 33282950 | mRNA | Luo | ENSG00000003987 | -0.2218 |
| Luo | 33282950 | mRNA | Luo | ENSG00000163082 | -0.3518 |
| Luo | 33282950 | mRNA | Luo | ENSG00000176153 | -0.4177 |
| Luo | 33282950 | mRNA | Luo | ENSG00000140287 | -0.6306 |
| Ma | 33680920 | mRNA | Ma | ENSG00000187151 | 0.3730 |
| Ma | 33680920 | mRNA | Ma | ENSG00000172216 | 0.1110 |
| Ma | 33680920 | mRNA | Ma | ENSG00000258839 | 0.1010 |
| Ma | 33680920 | mRNA | Ma | ENSG00000213903 | 0.0310 |
| Ma | 33680920 | mRNA | Ma | ENSG00000102003 | 0.0130 |
| Ma | 33680920 | mRNA | Ma | ENSG00000163515 | -0.0220 |
| Ma | 33680920 | mRNA | Ma | ENSG00000112175 | -0.0510 |
| Ma | 33680920 | mRNA | Ma | ENSG00000163739 | -0.0550 |
| Ma | 33680920 | mRNA | Ma | ENSG00000164251 | -0.0810 |
| Ma | 33680920 | mRNA | Ma | ENSG00000109819 | -0.1410 |
| Ma | 33680920 | mRNA | Ma | ENSG00000253729 | -0.1940 |
| Ma | 33680920 | mRNA | Ma | ENSG00000183134 | -0.0350 |
| Ma | 33680920 | mRNA | Ma | FGAB1 | -0.1830 |
| Mao | 33732694 | mRNA | Mao | ENSG00000122861 | 0.3049 |
| Mao | 33732694 | mRNA | Mao | ENSG00000125378 | 0.1927 |
| Mao | 33732694 | mRNA | Mao | ENSG00000115590 | -0.0968 |
| Mao | 33732694 | mRNA | Mao | ENSG00000139292 | -0.1572 |
| Mao | 33732694 | mRNA | Mao | ENSG00000163734 | -0.1689 |
| Mao | 33732694 | mRNA | Mao | ENSG00000100453 | -0.2983 |
| Mao | 33732694 | mRNA | Mao | ENSG00000168229 | -0.5107 |
| Martinez | 30537927 | mRNA | Martinez | ENSG00000169826 | -0.1922 |
| Martinez | 30537927 | mRNA | Martinez | ENSG00000057019 | 0.0274 |
| Martinez | 30537927 | mRNA | Martinez | ENSG00000099860 | 0.0521 |
| Martinez | 30537927 | mRNA | Martinez | ENSG00000125869 | 0.2817 |
| Martinez | 30537927 | mRNA | Martinez | ENSG00000135338 | -0.1246 |
| Martinez | 30537927 | mRNA | Martinez | ENSG00000186007 | 0.0165 |
| Martinez | 30537927 | mRNA | Martinez | ENSG00000113389 | -0.0733 |
| Martinez | 30537927 | mRNA | Martinez | ENSG00000152104 | 0.3016 |
| Martinez | 30537927 | mRNA | Martinez | ENSG00000059804 | 0.0895 |
| Martinez | 30537927 | mRNA | Martinez | ENSG00000169908 | -0.1160 |
| Matsuyama | 32657428 | mRNA | Matsuyama | ENSG00000133110 | 0.5669 |
| Matsuyama | 32657428 | mRNA | Matsuyama | ENSG00000168542 | 0.4193 |
| Matsuyama | 32657428 | mRNA | Matsuyama | ENSG00000163430 | 0.3786 |
| Matsuyama | 32657428 | mRNA | Matsuyama | ENSG00000164692 | 0.3392 |
| Matsuyama | 32657428 | mRNA | Matsuyama | ENSG00000115414 | 0.0663 |
| Matsuyama | 32657428 | mRNA | Matsuyama | ENSG00000060982 | -0.1568 |
| Matsuyama | 32657428 | mRNA | Matsuyama | ENSG00000050165 | -0.1997 |
| Miao | 32953273 | mRNA | Miao | ENSG00000173175 | 0.1465 |
| Miao | 32953273 | mRNA | Miao | ENSG00000198610 | 0.0283 |
| Miao | 32953273 | mRNA | Miao | ENSG00000211445 | 0.0110 |
| Miao | 32953273 | mRNA | Miao | ENSG00000176170 | 0.0075 |
| Miao | 32953273 | mRNA | Miao | ENSG00000158125 | -0.0293 |
| Miao | 32953273 | mRNA | Miao | ENSG00000156006 | -0.0631 |
| Mo-Car1 | 30933267 | mRNA | Mo-Car1 | ENSG00000162909 | 5.2400 |
| Mo-Car1 | 30933267 | mRNA | Mo-Car1 | ENSG00000073282 | 3.0670 |
| Mo-Car1 | 30933267 | mRNA | Mo-Car1 | ENSG00000057663 | 0.5620 |
| Mo-Car1 | 30933267 | mRNA | Mo-Car1 | ENSG00000108443 | -0.0450 |
| Mo-Car1 | 30933267 | mRNA | Mo-Car1 | ENSG00000168010 | -0.0950 |
| Mo-Car1 | 30933267 | mRNA | Mo-Car1 | ENSG00000096717 | -0.1780 |
| Mo-Car1 | 30933267 | mRNA | Mo-Car1 | ENSG00000034693 | -0.6960 |
| Mo-Car1 | 30933267 | mRNA | Mo-Car1 | ENSG00000198382 | -0.9570 |
| Mo-Car1 | 30933267 | mRNA | Mo-Car1 | ENSG00000145414 | -1.1240 |
| Mo-Car2 | 30933267 | mRNA | Mo-Car2 | ENSG00000162909 | 0.7950 |
| Mo-Car2 | 30933267 | mRNA | Mo-Car2 | ENSG00000073282 | 0.4070 |
| Mo-Car2 | 30933267 | mRNA | Mo-Car2 | ENSG00000057663 | 0.1260 |
| Mo-Car2 | 30933267 | mRNA | Mo-Car2 | ENSG00000034693 | -0.0090 |
| Mo-Car2 | 30933267 | mRNA | Mo-Car2 | ENSG00000198382 | -0.0180 |
| Mo-Car2 | 30933267 | mRNA | Mo-Car2 | ENSG00000168010 | -0.0790 |
| Mo-Car2 | 30933267 | mRNA | Mo-Car2 | ENSG00000145414 | -0.0960 |
| Mo-Car2 | 30933267 | mRNA | Mo-Car2 | ENSG00000096717 | -0.3240 |
| Mo-Car2 | 30933267 | mRNA | Mo-Car2 | ENSG00000108443 | -0.4120 |
| Mo-FICDB | 34211976 | mRNA | Mo-FICDB | ENSG00000150093 | 0.7701 |
| Mo-FICDB | 34211976 | mRNA | Mo-FICDB | ENSG00000113361 | 0.7659 |
| Mo-FICDB | 34211976 | mRNA | Mo-FICDB | ENSG00000107796 | 0.6310 |
| Mo-FICDB | 34211976 | mRNA | Mo-FICDB | ENSG00000151914 | 0.4995 |
| Mo-FICDB | 34211976 | mRNA | Mo-FICDB | ENSG00000198934 | 0.4941 |
| Mo-FICDB | 34211976 | mRNA | Mo-FICDB | ENSG00000179163 | 0.4319 |
| Mo-FICDB | 34211976 | mRNA | Mo-FICDB | ENSG00000152661 | 0.2274 |
| Mo-FICDB | 34211976 | mRNA | Mo-FICDB | ENSG00000106397 | 0.2229 |
| Mo-FICDB | 34211976 | mRNA | Mo-FICDB | ENSG00000006016 | 0.2182 |
| Mo-FICDB | 34211976 | mRNA | Mo-FICDB | ENSG00000135318 | 0.2159 |
| Mo-FICDB | 34211976 | mRNA | Mo-FICDB | ENSG00000107984 | 0.1861 |
| Mo-FICDB | 34211976 | mRNA | Mo-FICDB | ENSG00000058085 | 0.1347 |
| Mo-FICDB | 34211976 | mRNA | Mo-FICDB | ENSG00000163739 | -0.1613 |
| Mo-FICDB | 34211976 | mRNA | Mo-FICDB | ENSG00000107562 | -0.2058 |
| Mo-FICDB | 34211976 | mRNA | Mo-FICDB | ENSG00000106484 | -0.2061 |
| Mo-FICDB | 34211976 | mRNA | Mo-FICDB | ENSG00000164136 | -0.2940 |
| Mo-FICDB | 34211976 | mRNA | Mo-FICDB | ENSG00000113657 | -0.3017 |
| Mo-FICDB | 34211976 | mRNA | Mo-FICDB | ENSG00000152952 | -0.3064 |
| Mo-FICDB | 34211976 | mRNA | Mo-FICDB | ENSG00000196569 | -0.3587 |
| Mo-OI | 33117604 | mRNA | Mo-OI | ENSG00000141293 | 0.6650 |
| Mo-OI | 33117604 | mRNA | Mo-OI | ENSG00000196154 | 0.3290 |
| Mo-OI | 33117604 | mRNA | Mo-OI | ENSG00000162344 | 0.3190 |
| Mo-OI | 33117604 | mRNA | Mo-OI | ENSG00000184292 | 0.1820 |
| Mo-OI | 33117604 | mRNA | Mo-OI | ENSG00000139292 | -0.2280 |
| Mo-OI | 33117604 | mRNA | Mo-OI | ENSG00000108344 | -0.9390 |
| Mo-OI | 33117604 | mRNA | Mo-OI | ENSG00000102962 | -1.3040 |
| Mu | 32596355 | lncRNA | Mu | ENSG00000233901 | 0.6140 |
| Mu | 32596355 | lncRNA | Mu | ENSG00000247095 | 0.5135 |
| Mu | 32596355 | lncRNA | Mu | ENSG00000271659 | 0.7444 |
| Mu | 32596355 | lncRNA | Mu | ENSG00000179523 | 0.6305 |
| Mu | 32596355 | lncRNA | Mu | ENSG00000228506 | 0.6051 |
| Mu | 32596355 | lncRNA | Mu | ENSG00000238158 | 0.5506 |
| Mu | 32596355 | lncRNA | Mu | ENSG00000288663 | 0.5232 |
| Mu | 32596355 | lncRNA | Mu | ENSG00000262089 | 0.4871 |
| Mu | 32596355 | lncRNA | Mu | ENSG00000228437 | 0.4503 |
| Mu | 32596355 | lncRNA | Mu | ENSG00000272913 | 0.3720 |
| Mu | 32596355 | lncRNA | Mu | ENSG00000256546 | 0.3566 |
| Mu | 32596355 | lncRNA | Mu | ENSG00000253308 | 0.3483 |
| Mu | 32596355 | lncRNA | Mu | ENSG00000272275 | 0.3052 |
| Mu | 32596355 | lncRNA | Mu | ENSG00000259955 | -1.0771 |
| Qian | 33747044 | mRNA | Qian | ENSG00000134259 | 0.9440 |
| Qian | 33747044 | mRNA | Qian | ENSG00000125255 | 0.8440 |
| Qian | 33747044 | mRNA | Qian | ENSG00000197943 | 0.4990 |
| Qian | 33747044 | mRNA | Qian | ENSG00000163794 | 0.4680 |
| Qian | 33747044 | mRNA | Qian | ENSG00000122641 | 0.1390 |
| Qian | 33747044 | mRNA | Qian | ENSG00000146469 | 0.0670 |
| Qian | 33747044 | mRNA | Qian | ENSG00000170323 | 0.0570 |
| Qian | 33747044 | mRNA | Qian | ENSG00000242076 | 0.0510 |
| Qian | 33747044 | mRNA | Qian | ENSG00000163515 | 0.0040 |
| Qian | 33747044 | mRNA | Qian | ENSG00000211640 | 0.0030 |
| Qian | 33747044 | mRNA | Qian | ENSG00000163734 | -0.0190 |
| Qian | 33747044 | mRNA | Qian | ENSG00000164251 | -0.0270 |
| Qian | 33747044 | mRNA | Qian | ENSG00000181092 | -0.2490 |
| Qian | 33747044 | mRNA | Qian | ENSG00000180914 | -0.3040 |
| Qian | 33747044 | mRNA | Qian | ENSG00000064300 | -0.4360 |
| Qian | 33747044 | mRNA | Qian | ENSG00000086991 | -1.2530 |
| Qian | 33747044 | mRNA | Qian | ENSG00000158485 | -4.7260 |
| Qian | 33747044 | mRNA | Qian | ENSG00000211829 | 0.2670 |
| Qin | 33959151 | lncRNA | Qin | ENSG00000236081 | 0.3071 |
| Qin | 33959151 | lncRNA | Qin | ENSG00000245526 | 0.2826 |
| Qin | 33959151 | lncRNA | Qin | ENSG00000261105 | 0.2742 |
| Qin | 33959151 | lncRNA | Qin | ENSG00000171889 | 0.1507 |
| Qin | 33959151 | lncRNA | Qin | ENSG00000196758 | -0.1931 |
| Qin | 33959151 | lncRNA | Qin | ENSG00000235366 | -0.2275 |
| Qin | 33959151 | lncRNA | Qin | ENSG00000225506 | -0.2466 |
| Shan | 33660944 | mRNA | Shan | ENSG00000196924 | -0.0822 |
| Shan | 33660944 | mRNA | Shan | ENSG00000152377 | 0.2876 |
| Shan | 33660944 | mRNA | Shan | ENSG00000018408 | 0.2045 |
| Shan | 33660944 | mRNA | Shan | ENSG00000106366 | 0.1647 |
| Shan | 33660944 | mRNA | Shan | ENSG00000075618 | 0.1109 |
| Shan | 33660944 | mRNA | Shan | ENSG00000115380 | 0.0855 |
| Shan | 33660944 | mRNA | Shan | ENSG00000007952 | -0.0416 |
| Shan | 33660944 | mRNA | Shan | ENSG00000169429 | -0.1852 |
| Shan | 33660944 | mRNA | Shan | ENSG00000026025 | -0.7074 |
| Shan | 33660944 | mRNA | Shan | ENSG00000197405 | 0.3770 |
| Sun-BRI | 32953885 | mRNA | Sun-BRI | ENSG00000115361 | 7.0631 |
| Sun-BRI | 32953885 | mRNA | Sun-BRI | ENSG00000131480 | 0.8140 |
| Sun-BRI | 32953885 | mRNA | Sun-BRI | ENSG00000136960 | 0.0654 |
| Sun-BRI | 32953885 | mRNA | Sun-BRI | ENSG00000196839 | 0.0604 |
| Sun-BRI | 32953885 | mRNA | Sun-BRI | ENSG00000152642 | -0.0554 |
| Sun-BRI | 32953885 | mRNA | Sun-BRI | ENSG00000157184 | -0.1034 |
| Sun-JCP | 30132881 | mRNA | Sun-JCP | ENSG00000171951 | 0.0652 |
| Sun-JCP | 30132881 | mRNA | Sun-JCP | ENSG00000211445 | 0.0095 |
| Sun-JCP | 30132881 | mRNA | Sun-JCP | ENSG00000016490 | 0.0003 |
| Sun-JCP | 30132881 | mRNA | Sun-JCP | ENSG00000156006 | -0.0840 |
| Sun-JCP | 30132881 | mRNA | Sun-JCP | ENSG00000163808 | -0.1195 |
| Tian | 28796930 | mRNA | Tian | ENSG00000124006 | 0.4983 |
| Tian | 28796930 | mRNA | Tian | ENSG00000197930 | 0.4074 |
| Tian | 28796930 | mRNA | Tian | ENSG00000151748 | 0.3065 |
| Tian | 28796930 | mRNA | Tian | ENSG00000157103 | 0.2530 |
| Tian | 28796930 | mRNA | Tian | ENSG00000169213 | 0.2231 |
| Tian | 28796930 | mRNA | Tian | ENSG00000105971 | 0.0772 |
| Tian | 28796930 | mRNA | Tian | ENSG00000174938 | 0.0768 |
| Tian | 28796930 | mRNA | Tian | ENSG00000171951 | 0.0471 |
| Tian | 28796930 | mRNA | Tian | ENSG00000186340 | 0.0408 |
| Tian | 28796930 | mRNA | Tian | ENSG00000156535 | 0.0388 |
| Tian | 28796930 | mRNA | Tian | ENSG00000152104 | 0.0159 |
| Tian | 28796930 | mRNA | Tian | ENSG00000163956 | -0.0433 |
| Tian | 28796930 | mRNA | Tian | ENSG00000132313 | -0.0645 |
| Tokunaga | 32191346 | mRNA | Tokunaga | ENSG00000275385 | NA |
| Tokunaga | 32191346 | mRNA | Tokunaga | ENSG00000172724 | NA |
| Tokunaga | 32191346 | mRNA | Tokunaga | ENSG00000108691 | NA |
| Tokunaga | 32191346 | mRNA | Tokunaga | ENSG00000137077 | NA |
| Tokunaga | 32191346 | mRNA | Tokunaga | ENSG00000277632 | NA |
| Tokunaga | 32191346 | mRNA | Tokunaga | ENSG00000275302 | NA |
| Tokunaga | 32191346 | mRNA | Tokunaga | ENSG00000271503 | NA |
| Tokunaga | 32191346 | mRNA | Tokunaga | ENSG00000108700 | NA |
| Tokunaga | 32191346 | mRNA | Tokunaga | ENSG00000169245 | NA |
| Tokunaga | 32191346 | mRNA | Tokunaga | ENSG00000169248 | NA |
| Tokunaga | 32191346 | mRNA | Tokunaga | ENSG00000156234 | NA |
| Tokunaga | 32191346 | mRNA | Tokunaga | ENSG00000138755 | NA |
| Wang-BIO | 32036725 | mRNA | Wang-BIO | ENSG00000164889 | 0.8610 |
| Wang-BIO | 32036725 | mRNA | Wang-BIO | ENSG00000140650 | 0.8370 |
| Wang-BIO | 32036725 | mRNA | Wang-BIO | ENSG00000125352 | 0.6030 |
| Wang-BIO | 32036725 | mRNA | Wang-BIO | ENSG00000177125 | 0.5270 |
| Wang-BIO | 32036725 | mRNA | Wang-BIO | ENSG00000134461 | 0.4740 |
| Wang-BIO | 32036725 | mRNA | Wang-BIO | ENSG00000184916 | 0.3420 |
| Wang-BIO | 32036725 | mRNA | Wang-BIO | ENSG00000121742 | 0.1500 |
| Wang-BIO | 32036725 | mRNA | Wang-BIO | ENSG00000121764 | 0.1440 |
| Wang-BIO | 32036725 | mRNA | Wang-BIO | ENSG00000198944 | -0.2920 |
| Wang-BIO | 32036725 | mRNA | Wang-BIO | ENSG00000027001 | -0.3360 |
| Wang-BIO | 32036725 | mRNA | Wang-BIO | ENSG00000163808 | -0.4490 |
| Wang-BIO | 32036725 | mRNA | Wang-BIO | ENSG00000163684 | -0.4630 |
| Wang-BIO | 32036725 | mRNA | Wang-BIO | ENSG00000167553 | -0.6090 |
| Wang-BIO | 32036725 | mRNA | Wang-BIO | ENSG00000118705 | -0.7800 |
| Wang-BIO | 32036725 | mRNA | Wang-BIO | ENSG00000038532 | -1.5120 |
| Wang-CMR | 30510449 | lncRNA | Wang-CMR | ENSG00000232987 | 0.3570 |
| Wang-CMR | 30510449 | lncRNA | Wang-CMR | ENSG00000231768 | 0.3170 |
| Wang-CMR | 30510449 | lncRNA | Wang-CMR | ENSG00000249894 | 0.2380 |
| Wang-CMR | 30510449 | lncRNA | Wang-CMR | ENSG00000278546 | 0.2210 |
| Wang-CMR | 30510449 | lncRNA | Wang-CMR | ENSG00000254574 | 0.1610 |
| Wang-CMR | 30510449 | lncRNA | Wang-CMR | ENSG00000228437 | 0.1390 |
| Wang-CMR | 30510449 | lncRNA | Wang-CMR | ENSG00000279605 | 0.1260 |
| Wang-CMR | 30510449 | lncRNA | Wang-CMR | ENSG00000254337 | 0.0820 |
| Wang-CMR | 30510449 | lncRNA | Wang-CMR | ENSG00000260597 | 0.0670 |
| Wang-CMR | 30510449 | lncRNA | Wang-CMR | ENSG00000253931 | 0.0600 |
| Wang-CMR | 30510449 | lncRNA | Wang-CMR | ENSG00000230798 | 0.0530 |
| Wang-CMR | 30510449 | lncRNA | Wang-CMR | ENSG00000224817 | 0.0310 |
| Wang-CMR | 30510449 | lncRNA | Wang-CMR | ENSG00000229960 | -0.1130 |
| Wang-CMR | 30510449 | lncRNA | Wang-CMR | ENSG00000238042 | -0.1310 |
| Wang-CMR | 30510449 | lncRNA | Wang-CMR | ENSG00000196758 | -0.2690 |
| Wang-FICDB | 33224957 | mRNA | Wang-FICDB | ENSG00000146453 | 0.3411 |
| Wang-FICDB | 33224957 | mRNA | Wang-FICDB | ENSG00000149289 | -0.5883 |
| Wang-FICDB | 33224957 | mRNA | Wang-FICDB | ENSG00000109819 | -0.8399 |
| Wang-FICDB | 33224957 | mRNA | Wang-FICDB | ENSG00000151846 | -0.9978 |
| Wang-FICDB | 33224957 | mRNA | Wang-FICDB | ENSG00000196116 | -1.0640 |
| Wang-FICDB | 33224957 | mRNA | Wang-FICDB | ENSG00000093167 | -1.3003 |
| Wang-FICDB | 33224957 | mRNA | Wang-FICDB | ENSG00000180113 | -2.0109 |
| Wang-FIG | 33719345 | mRNA | Wang-FIG | ENSG00000147082 | 3.5000 |
| Wang-FIG | 33719345 | mRNA | Wang-FIG | ENSG00000077935 | 0.4800 |
| Wang-FIG | 33719345 | mRNA | Wang-FIG | ENSG00000143429 | 0.3400 |
| Wang-FIG | 33719345 | mRNA | Wang-FIG | ENSG00000128886 | 0.3000 |
| Wang-FIG | 33719345 | mRNA | Wang-FIG | ENSG00000240682 | 0.2700 |
| Wang-FIG | 33719345 | mRNA | Wang-FIG | ENSG00000258839 | 0.2600 |
| Wang-FIG | 33719345 | mRNA | Wang-FIG | ENSG00000135218 | 0.1900 |
| Wang-FIG | 33719345 | mRNA | Wang-FIG | ENSG00000070159 | 0.1100 |
| Wang-FIG | 33719345 | mRNA | Wang-FIG | ENSG00000140521 | 0.1100 |
| Wang-FIG | 33719345 | mRNA | Wang-FIG | ENSG00000140416 | -0.0390 |
| Wang-FIG | 33719345 | mRNA | Wang-FIG | ENSG00000158402 | -0.0810 |
| Wang-FIG | 33719345 | mRNA | Wang-FIG | ENSG00000114904 | -0.4600 |
| Wang-FO | 33910362 | lncRNA | Wang-FO | ENSG00000179406 | 0.6742 |
| Wang-FO | 33910362 | lncRNA | Wang-FO | ENSG00000225265 | 0.4466 |
| Wang-FO | 33910362 | lncRNA | Wang-FO | ENSG00000166770 | 0.4241 |
| Wang-FO | 33910362 | lncRNA | Wang-FO | ENSG00000196741 | 0.3945 |
| Wang-FO | 33910362 | lncRNA | Wang-FO | ENSG00000242094 | -0.4840 |
| Wang-FO | 33910362 | lncRNA | Wang-FO | ENSG00000231177 | -0.9109 |
| Wang-JCMM | 32564470 | mRNA | Wang-JCMM | ENSG00000125255 | 0.6390 |
| Wang-JCMM | 32564470 | mRNA | Wang-JCMM | ENSG00000138685 | 0.3870 |
| Wang-JCMM | 32564470 | mRNA | Wang-JCMM | ENSG00000163794 | 0.3780 |
| Wang-JCMM | 32564470 | mRNA | Wang-JCMM | ENSG00000049247 | 0.2540 |
| Wang-JCMM | 32564470 | mRNA | Wang-JCMM | ENSG00000164283 | 0.1240 |
| Wang-JCMM | 32564470 | mRNA | Wang-JCMM | ENSG00000104419 | 0.0120 |
| Wang-JCMM | 32564470 | mRNA | Wang-JCMM | ENSG00000151882 | -0.0940 |
| Wang-JCMM | 32564470 | mRNA | Wang-JCMM | ENSG00000211829 | 0.1290 |
| Wang-Med | 33787596 | mRNA | Wang-Med | ENSG00000132002 | 0.5989 |
| Wang-Med | 33787596 | mRNA | Wang-Med | ENSG00000162734 | 0.0973 |
| Wang-Med | 33787596 | mRNA | Wang-Med | ENSG00000124333 | -0.1977 |
| Wang-Med | 33787596 | mRNA | Wang-Med | ENSG00000142655 | -0.1584 |
| Wang-Med | 33787596 | mRNA | Wang-Med | ENSG00000119782 | 0.2351 |
| Wang-Med | 33787596 | mRNA | Wang-Med | ENSG00000164741 | 0.1991 |
| Wang-ONCO | 29774095 | lncRNA | Wang-ONCO | ENSG00000224259 | -0.3360 |
| Wang-ONCO | 29774095 | lncRNA | Wang-ONCO | ENSG00000281881 | 0.3220 |
| Wang-ONCO | 29774095 | lncRNA | Wang-ONCO | ENSG00000171889 | -0.1340 |
| Wang-ONCO | 29774095 | lncRNA | Wang-ONCO | ENSG00000269463 | -0.2310 |
| Wei | 33149738 | lncRNA | Wei | ENSG00000237036 | 0.5219 |
| Wei | 33149738 | lncRNA | Wei | ENSG00000233901 | 0.2220 |
| Wei | 33149738 | lncRNA | Wei | ENSG00000269680 | 0.3650 |
| Wei | 33149738 | lncRNA | Wei | ENSG00000261628 | 0.2007 |
| Wei | 33149738 | lncRNA | Wei | ENSG00000259953 | 0.1612 |
| Wei | 33149738 | lncRNA | Wei | ENSG00000253930 | -0.1420 |
| Wei | 33149738 | lncRNA | Wei | ENSG00000279006 | -0.3599 |
| Wei | 33149738 | lncRNA | Wei | ENSG00000257740 | -0.5895 |
| Wu | 32908876 | mRNA | Wu | ENSG00000102962 | -0.4258 |
| Wu | 32908876 | mRNA | Wu | ENSG00000135218 | 0.1784 |
| Wu | 32908876 | mRNA | Wu | ENSG00000163751 | -0.3294 |
| Wu | 32908876 | mRNA | Wu | ENSG00000169169 | 0.1207 |
| Wu | 32908876 | mRNA | Wu | ENSG00000152049 | -0.1548 |
| Wu | 32908876 | mRNA | Wu | ENSG00000131196 | 0.2430 |
| Wu | 32908876 | mRNA | Wu | ENSG00000068831 | 0.2297 |
| Wu | 32908876 | mRNA | Wu | ENSG00000059804 | 0.2177 |
| Xia | 33663535 | mRNA | Xia | ENSG00000153936 | 0.5377 |
| Xia | 33663535 | mRNA | Xia | ENSG00000143575 | 0.6679 |
| Xia | 33663535 | mRNA | Xia | ENSG00000115677 | 0.5384 |
| Xia | 33663535 | mRNA | Xia | ENSG00000113552 | 0.4753 |
| Xia | 33663535 | mRNA | Xia | ENSG00000117632 | 0.3651 |
| Xia | 33663535 | mRNA | Xia | ENSG00000128039 | 0.3543 |
| Xia | 33663535 | mRNA | Xia | ENSG00000125826 | 0.3459 |
| Xia | 33663535 | mRNA | Xia | ENSG00000170312 | -0.3001 |
| Xia | 33663535 | mRNA | Xia | ENSG00000167588 | -0.3413 |
| Xia | 33663535 | mRNA | Xia | ENSG00000213024 | -0.6322 |
| Xu | 33324651 | mRNA | Xu | ENSG00000196730 | 0.1281 |
| Xu | 33324651 | mRNA | Xu | ENSG00000117984 | 0.0881 |
| Xu | 33324651 | mRNA | Xu | ENSG00000147889 | 0.0836 |
| Xu | 33324651 | mRNA | Xu | ENSG00000168397 | 0.0707 |
| Xu | 33324651 | mRNA | Xu | ENSG00000140474 | 0.0676 |
| Xu | 33324651 | mRNA | Xu | ENSG00000177169 | 0.0192 |
| Xu | 33324651 | mRNA | Xu | ENSG00000157168 | -0.1192 |
| Xu | 33324651 | mRNA | Xu | ENSG00000197249 | -0.1536 |
| Xue | 29254165 | lncRNA | Xue | ENSG00000245910 | -0.9210 |
| Xue | 29254165 | lncRNA | Xue | ENSG00000261780 | 0.8005 |
| Yang | 33537062 | mRNA | Yang | ENSG00000086475 | 0.0569 |
| Yang | 33537062 | mRNA | Yang | ENSG00000128567 | 0.0328 |
| Yang | 33537062 | mRNA | Yang | ENSG00000171792 | 0.0205 |
| Yang | 33537062 | mRNA | Yang | ENSG00000099194 | 0.0029 |
| Yang | 33537062 | mRNA | Yang | ENSG00000169299 | -0.1409 |
| Ye | 32175389 | mRNA | Ye | ENSG00000138495 | 0.5184 |
| Ye | 32175389 | mRNA | Ye | ENSG00000151131 | 0.1520 |
| Ye | 32175389 | mRNA | Ye | ENSG00000204220 | 0.1260 |
| Ye | 32175389 | mRNA | Ye | ENSG00000188732 | 0.0214 |
| Ye | 32175389 | mRNA | Ye | ENSG00000099974 | -0.0014 |
| Ye | 32175389 | mRNA | Ye | ENSG00000143554 | -0.0091 |
| Ye | 32175389 | mRNA | Ye | ENSG00000168036 | -0.0541 |
| Ye | 32175389 | mRNA | Ye | ENSG00000167600 | -0.0649 |
| Ye | 32175389 | mRNA | Ye | ENSG00000162390 | -0.0733 |
| Ye | 32175389 | mRNA | Ye | ENSG00000160867 | -0.0959 |
| Ye | 32175389 | mRNA | Ye | ENSG00000141655 | -0.1105 |
| Ye | 32175389 | mRNA | Ye | ENSG00000011376 | -0.1444 |
| Ye | 32175389 | mRNA | Ye | ENSG00000167065 | -0.1537 |
| Ye | 32175389 | mRNA | Ye | ENSG00000016391 | -0.1788 |
| Ye | 32175389 | mRNA | Ye | ENSG00000137171 | -0.2550 |
| Yuan | 32509568 | mRNA | Yuan | ENSG00000170122 | 0.8930 |
| Yuan | 32509568 | mRNA | Yuan | ENSG00000138792 | 0.2340 |
| Yuan | 32509568 | mRNA | Yuan | ENSG00000197757 | 0.1730 |
| Yuan | 32509568 | mRNA | Yuan | ENSG00000179593 | 0.0610 |
| Yue-Aging | 33658390 | mRNA | Yue-Aging | ENSG00000135218 | 0.0405 |
| Yue-Aging | 33658390 | mRNA | Yue-Aging | ENSG00000112715 | 0.0201 |
| Yue-Aging | 33658390 | mRNA | Yue-Aging | ENSG00000099139 | 0.0167 |
| Yue-Aging | 33658390 | mRNA | Yue-Aging | ENSG00000111206 | 0.0117 |
| Yue-Aging | 33658390 | mRNA | Yue-Aging | ENSG00000146674 | 0.0037 |
| Yue-Aging | 33658390 | mRNA | Yue-Aging | ENSG00000120885 | 0.0017 |
| Yue-Aging | 33658390 | mRNA | Yue-Aging | ENSG00000102265 | 0.0014 |
| Yue-Aging | 33658390 | mRNA | Yue-Aging | ENSG00000211896 | 0.0000 |
| Yue-Aging | 33658390 | mRNA | Yue-Aging | ENSG00000162896 | -0.0002 |
| Yue-Aging | 33658390 | mRNA | Yue-Aging | ENSG00000163739 | -0.0002 |
| Yue-Aging | 33658390 | mRNA | Yue-Aging | ENSG00000151882 | -0.0010 |
| Yue-Aging | 33658390 | mRNA | Yue-Aging | ENSG00000118705 | -0.0020 |
| Yue-Aging | 33658390 | mRNA | Yue-Aging | ENSG00000134057 | -0.0048 |
| Yue-Aging | 33658390 | mRNA | Yue-Aging | ENSG00000060762 | -0.0083 |
| Yue-Aging | 33658390 | mRNA | Yue-Aging | ENSG00000166851 | -0.0130 |
| Yue-BRI | 33376727 | mRNA | Yue-BRI | ENSG00000105281 | 0.6579 |
| Yue-BRI | 33376727 | mRNA | Yue-BRI | ENSG00000109452 | 0.3337 |
| Yue-BRI | 33376727 | mRNA | Yue-BRI | ENSG00000197122 | -0.7350 |
| Yue-BRI | 33376727 | mRNA | Yue-BRI | ENSG00000105173 | -0.7475 |
| Yue-BRI | 33376727 | mRNA | Yue-BRI | ENSG00000117676 | -0.8763 |
| Yue-BRI | 33376727 | mRNA | Yue-BRI | ENSG00000146648 | 0.8318 |
| Zeng | 28187432 | lncRNA | Zeng | ENSG00000180869 | -0.1910 |
| Zeng | 28187432 | lncRNA | Zeng | ENSG00000259240 | 0.3180 |
| Zeng | 28187432 | lncRNA | Zeng | ENSG00000248771 | -0.1630 |
| Zeng | 28187432 | lncRNA | Zeng | ENSG00000251611 | -0.3380 |
| Zhang-EBM | 32605475 | mRNA | Zhang-EBM | ENSG00000047188 | -0.2475 |
| Zhang-EBM | 32605475 | mRNA | Zhang-EBM | ENSG00000136231 | 0.1530 |
| Zhang-JCB | 30320902 | lncRNA | Zhang-JCB | ENSG00000268658 | 2.4030 |
| Zhang-JCB | 30320902 | lncRNA | Zhang-JCB | ENSG00000261824 | 1.4820 |
| Zhang-JCB | 30320902 | lncRNA | Zhang-JCB | ENSG00000267058 | 1.1798 |
| Zhang-JCB | 30320902 | lncRNA | Zhang-JCB | ENSG00000253716 | 0.9237 |
| Zhang-JCB | 30320902 | lncRNA | Zhang-JCB | ENSG00000233522 | 2.9397 |
| Zhang-JCB | 30320902 | lncRNA | Zhang-JCB | ENSG00000223768 | 2.4646 |
| Zhang-JCB | 30320902 | lncRNA | Zhang-JCB | ENSG00000225756 | 2.0437 |
| Zhang-JCB | 30320902 | lncRNA | Zhang-JCB | ENSG00000286125 | 0.7759 |
| Zhang-JTM | 31796117 | mRNA | Zhang-JTM | ENSG00000121390 | 1.1460 |
| Zhang-JTM | 31796117 | mRNA | Zhang-JTM | ENSG00000166598 | 1.0930 |
| Zhang-JTM | 31796117 | mRNA | Zhang-JTM | ENSG00000129292 | 1.0650 |
| Zhang-JTM | 31796117 | mRNA | Zhang-JTM | ENSG00000170456 | 1.0380 |
| Zhang-JTM | 31796117 | mRNA | Zhang-JTM | ENSG00000102409 | 0.7110 |
| Zhang-JTM | 31796117 | mRNA | Zhang-JTM | ENSG00000123684 | 0.7050 |
| Zhang-JTM | 31796117 | mRNA | Zhang-JTM | ENSG00000168209 | 0.6720 |
| Zhang-JTM | 31796117 | mRNA | Zhang-JTM | ENSG00000129451 | 0.4460 |
| Zhang-JTM | 31796117 | mRNA | Zhang-JTM | ENSG00000197757 | 0.2490 |
| Zhang-JTM | 31796117 | mRNA | Zhang-JTM | ENSG00000169946 | -0.6900 |
| Zhang-JTM | 31796117 | mRNA | Zhang-JTM | ENSG00000112715 | -0.8340 |
| Zhang-JTM | 31796117 | mRNA | Zhang-JTM | ENSG00000108061 | -0.9040 |
| Zhang-JTM | 31796117 | mRNA | Zhang-JTM | ENSG00000117560 | -1.2430 |
| Zhang-JTM | 31796117 | mRNA | Zhang-JTM | ENSG00000116688 | -1.4890 |
| Zhang-JTM | 31796117 | mRNA | Zhang-JTM | ENSG00000111405 | -4.2730 |
| Zhang-Med | 32569190 | mRNA | Zhang-Med | ENSG00000137801 | 0.2989 |
| Zhang-Med | 32569190 | mRNA | Zhang-Med | ENSG00000161958 | 0.3787 |
| Zhang-Med | 32569190 | mRNA | Zhang-Med | ENSG00000132693 | 0.2587 |
| Zhang-Med | 32569190 | mRNA | Zhang-Med | ENSG00000143125 | 0.2304 |
| Zhang-Med | 32569190 | mRNA | Zhang-Med | ENSG00000189334 | -0.1192 |
| Zhang-Med | 32569190 | mRNA | Zhang-Med | ENSG00000172724 | -0.3350 |
| Zhang-PeeJ1 | 30564521 | lncRNA | Zhang-PeeJ1 | ENSG00000272180 | 4.3132 |
| Zhang-PeeJ1 | 30564521 | lncRNA | Zhang-PeeJ1 | ENSG00000259347 | 0.8131 |
| Zhang-PeeJ1 | 30564521 | lncRNA | Zhang-PeeJ1 | ENSG00000244541 | 0.4497 |
| Zhang-PeeJ1 | 30564521 | lncRNA | Zhang-PeeJ1 | ENSG00000253308 | 0.1337 |
| Zhang-PeeJ1 | 30564521 | lncRNA | Zhang-PeeJ1 | ENSG00000277247 | 0.0633 |
| Zhang-PeeJ1 | 30564521 | lncRNA | Zhang-PeeJ1 | ENSG00000225335 | -1.5194 |
| Zhang-PeeJ2 | 30564521 | lncRNA | Zhang-PeeJ2 | ENSG00000180869 | -0.1910 |
| Zhang-PeeJ2 | 30564521 | lncRNA | Zhang-PeeJ2 | ENSG00000259240 | 0.3180 |
| Zhang-PeeJ2 | 30564521 | lncRNA | Zhang-PeeJ2 | ENSG00000259240 | -0.1630 |
| Zhang-PeeJ2 | 30564521 | lncRNA | Zhang-PeeJ2 | ENSG00000248771 | -0.1910 |
| Zhao | 34168974 | mRNA | Zhao | ENSG00000073282 | 1.4333 |
| Zhao | 34168974 | mRNA | Zhao | ENSG00000140474 | 0.5014 |
| Zhao | 34168974 | mRNA | Zhao | ENSG00000135047 | 0.3298 |
| Zhao | 34168974 | mRNA | Zhao | ENSG00000109971 | -0.5319 |
| Zhao | 34168974 | mRNA | Zhao | ENSG00000050748 | -0.7018 |
| Zheng | 32453965 | mRNA | Zheng | ENSG00000074317 | 0.2023 |
| Zheng | 32453965 | mRNA | Zheng | ENSG00000151224 | 0.1767 |
| Zheng | 32453965 | mRNA | Zheng | ENSG00000163794 | 0.1456 |
| Zheng | 32453965 | mRNA | Zheng | ENSG00000163283 | 0.1260 |
| Zheng | 32453965 | mRNA | Zheng | ENSG00000137285 | 0.1152 |
| Zheng | 32453965 | mRNA | Zheng | ENSG00000109158 | -0.0869 |
| Zheng | 32453965 | mRNA | Zheng | ENSG00000002726 | -0.2682 |
| Zheng | 32453965 | mRNA | Zheng | ENSG00000129422 | -0.3614 |
| Zheng | 32453965 | mRNA | Zheng | ENSG00000117399 | -0.4684 |
| Zhou2019 | 31552190 | mRNA | Zhou2019 | ENSG00000065135 | 1.0676 |
| Zhou2019 | 31552190 | mRNA | Zhou2019 | ENSG00000128590 | 0.8265 |
| Zhou2019 | 31552190 | mRNA | Zhou2019 | ENSG00000142330 | 0.8200 |
| Zhou2019 | 31552190 | mRNA | Zhou2019 | ENSG00000087074 | 0.2178 |
| Zhou2019 | 31552190 | mRNA | Zhou2019 | ENSG00000035664 | -0.0392 |
| Zhou2020 | 33102231 | mRNA | Zhou2020 | ENSG00000122025 | 1.0612 |
| Zhou2020 | 33102231 | mRNA | Zhou2020 | ENSG00000135218 | 0.6497 |
| Zhou2020 | 33102231 | mRNA | Zhou2020 | ENSG00000005884 | 0.5057 |
| Zhou2020 | 33102231 | mRNA | Zhou2020 | ENSG00000117322 | 0.4381 |
| Zhou2020 | 33102231 | mRNA | Zhou2020 | ENSG00000116824 | 0.4341 |
| Zhou2020 | 33102231 | mRNA | Zhou2020 | ENSG00000104432 | 0.2417 |
| Zhou2020 | 33102231 | mRNA | Zhou2020 | ENSG00000196352 | 0.0347 |
| Zhu2020 | 32781411 | mRNA | Zhu2020 | ENSG00000174697 | 0.1524 |
| Zhu2020 | 32781411 | mRNA | Zhu2020 | ENSG00000184502 | 0.1261 |
| Zhu2020 | 32781411 | mRNA | Zhu2020 | ENSG00000160349 | 0.1066 |
| Zhu2020 | 32781411 | mRNA | Zhu2020 | ENSG00000131015 | 0.0936 |
| Zhu2020 | 32781411 | mRNA | Zhu2020 | ENSG00000183395 | -0.4432 |
| Zhu2021 | 33747908 | mRNA | Zhu2021 | ENSG00000108515 | 0.3989 |
| Zhu2021 | 33747908 | mRNA | Zhu2021 | ENSG00000061656 | 0.1174 |
| Zhu2021 | 33747908 | mRNA | Zhu2021 | ENSG00000113739 | 0.0442 |
| Zhu2021 | 33747908 | mRNA | Zhu2021 | ENSG00000063660 | 0.0269 |
| Zhu2021 | 33747908 | mRNA | Zhu2021 | ENSG00000122884 | 0.0239 |
| Zou | 31572060 | mRNA | Zou | ENSG00000175938 | 0.1120 |
| Zou | 31572060 | mRNA | Zou | ENSG00000165140 | 0.0820 |
| Zou | 31572060 | mRNA | Zou | ENSG00000112715 | 0.0733 |
| Zou | 31572060 | mRNA | Zou | ENSG00000167508 | 0.0430 |
| Zou | 31572060 | mRNA | Zou | ENSG00000183087 | 0.0140 |
| Zou | 31572060 | mRNA | Zou | ENSG00000100243 | -0.0030 |
| Zou | 31572060 | mRNA | Zou | ENSG00000196323 | -0.0030 |
| Zou | 31572060 | mRNA | Zou | ENSG00000135679 | -0.0130 |
| Zou | 31572060 | mRNA | Zou | ENSG00000186314 | -0.0250 |
| Zou | 31572060 | mRNA | Zou | ENSG00000113328 | -0.0260 |
| Zou | 31572060 | mRNA | Zou | ENSG00000138439 | -0.0320 |
| Zou | 31572060 | mRNA | Zou | ENSG00000138794 | -0.0450 |
| Zou | 31572060 | mRNA | Zou | ENSG00000131323 | -0.0600 |
| Zou | 31572060 | mRNA | Zou | ENSG00000160208 | -0.1290 |
| Zuo | 33767290 | mRNA | Zuo | ENSG00000107317 | 0.0102 |
| Zuo | 33767290 | mRNA | Zuo | ENSG00000211445 | 0.0076 |
| Zuo | 33767290 | mRNA | Zuo | ENSG00000067225 | 0.0011 |
| Zuo | 33767290 | mRNA | Zuo | ENSG00000153395 | 0.0052 |
| Zuo | 33767290 | mRNA | Zuo | ENSG00000135220 | -0.0041 |
| Zuo | 33767290 | mRNA | Zuo | ENSG00000167315 | -0.0044 |
| Zuo | 33767290 | mRNA | Zuo | ENSG00000145293 | -0.0060 |
| Zuo | 33767290 | mRNA | Zuo | ENSG00000079462 | -0.0119 |
| Zuo | 33767290 | mRNA | Zuo | ENSG00000173175 | 0.0934 |
| Zuo | 33767290 | mRNA | Zuo | ENSG00000161533 | -0.0461 |
| Zuo | 33767290 | mRNA | Zuo | ENSG00000157184 | -0.0578 |
| Zuo | 30627052 | mRNA | Zuo | ENSG00000167311 | 0.2318 |
| Zuo | 30627052 | mRNA | Zuo | ENSG00000080224 | 0.0951 |
| Zuo | 30627052 | mRNA | Zuo | ENSG00000102265 | 0.2263 |
