# Supplementary material for: Robust machine−learning based prognostic index using cytotoxic T lymphocyte evasion genes highlights potential therapeutic targets in colorectal cancer
Source: Cancer Cell Int. 2024 Jan 31;24:52. doi: 10.1186/s12935-024-03239-y (PMC10829178; doi:10.1186/s12935-024-03239-y)
Supplement: Supplementary file 1 — Additional file 1: Figure S1. (A) Expression and (B) prognostic significance of 31 core CERGs in TCGA-CRC dataset. Figure S2. IHC score of HOXC6 (A), G0S2 (B), and MX2 (C) in normal tissues and CRC. **p < 0.01; ***p < 0.001. Table S1. Published signatures applied for model comparison. Table S2. Sequences for qRT-PCR primers. Table S3. Detailed si-RNA sequences used in the study. Table S4. 182 CERGs from published research and 1793 IRGs from Immport database. Table S5. Published signatures applied for model comparison. C-index of each combination of machine learning method for developing the prognostic signature. Table S6. AUC value of each combination of machine learning method for constructing the immunotherapy-related signature. [file 12935_2024_3239_MOESM1_ESM.zip › Supplementary Material/Supplementary Table S4.docx]

**Supplementary Table S4: 182 CERGs from published research and 1793 IRGs from Immport database.**

| Only CTL-evasion | Only ImmPort Database | CTL-evasion AND ImmPort Database |
| --- | --- | --- |
| ATG10 | AZGP1 | B2M |
| ATG101 | CANX | IFNGR1 |
| CFLAR | CD1A | JAK2 |
| FITM2 | CD1B | SOCS1 |
| GPAA1 | CD1C | TAP1 |
| GPI1 | CD1D | TAP2 |
| OTUD5 | CD1E | TAPBP |
| PTPN2 | CD4 | ADAR |
| RBCK1 | CD8A | IFNGR2 |
| STAT2 | CD8B | IKBKG |
| TRAF2 | CD74 | IRF1 |
| ATG12 | CREB1 | JAK1 |
| ATG3 | CTSB | STAT1 |
| ATG5 | CTSE | TNFAIP3 |
| CHIC2 | CTSL | TNFRSF1A |
| DNTTIP1 | CTSS | BECN1 |
| EMC8 | FCER1G | ERAP1 |
| ERP44 | FCGRT | HDAC1 |
| FADD | HFE | PDIA3 |
| IST1 | HLA-A | PSMB8 |
| MAP3K7 | HLA-B | TNFRSF1B |
| MEN1 | HLA-C | CALR |
| MOGS | HLA-DMA | FAS |
| NDUFA13 | HLA-DMB | IFNAR1 |
| NXT1 | HLA-DOA | IFNAR2 |
| OTULIN | HLA-DOB | IKBKB |
| PIGS | HLA-DPA1 | IRF9 |
| PIGU | HLA-DPB1 | MAPK1 |
| PPP2R2A | HLA-DQA1 | TBK1 |
| RB1CC1 | HLA-DQA2 | TFRC |
| RBM15 | HLA-DQB1 | TGFBR2 |
| RIC8 | HLA-DRA |  |
| RNF31 | HLA-DRB1 |  |
| SCAF4 | HLA-DRB3 |  |
| SMG7 | HLA-DRB4 |  |
| TAB1 | HLA-DRB5 |  |
| TAB2 | HLA-E |  |
| TMEM127 | HLA-F |  |
| UBE2G2 | HLA-G |  |
| UBE2N | HLA-H |  |
| VDAC2 | MR1 |  |
| VPS29 | HSPA1A |  |
| VPS35 | HSPA1B |  |
| VPS4B | HSPA1L |  |
| WIPI2 | HSPA2 |  |
| ZCCHC14 | HSPA4 |  |
| ACAD9 | HSPA5 |  |
| ACTB | HSPA6 |  |
| AGO2 | HSPA8 |  |
| AHSA1 | HSP90AA1 |  |
| ANAPC15 | HSP90AB1 |  |
| ARF6 | ICAM1 |  |
| ATG14 | IFNA1 |  |
| ATG7 | IFNA2 |  |
| ATG9A | IFNA4 |  |
| ATP13A1 | IFNA5 |  |
| ATXN7L3 | IFNA6 |  |
| BC003331 | IFNA7 |  |
| BCL2L1 | IFNA8 |  |
| BOLA3 | IFNA10 |  |
| BRAT1 | IFNA13 |  |
| BRPF1 | IFNA14 |  |
| CAD | IFNA16 |  |
| CEP55 | IFNA17 |  |
| CHMP5 | IFNA21 |  |
| COX6C | IFNG |  |
| CREBBP | KIR2DL1 |  |
| CUL3 | KIR2DL2 |  |
| DCP1A | KIR2DL3 |  |
| DET1 | KIR2DL4 |  |
| DICER1 | KIR2DS1 |  |
| DNAJC13 | KIR2DS3 |  |
| DOT1L | KIR2DS4 |  |
| DPH5 | KIR2DS5 |  |
| EIF3H | KIR3DL1 |  |
| EMC2 | KIR3DL2 |  |
| EMC3 | KLRC1 |  |
| EMC4 | KLRC2 |  |
| EMC6 | KLRC3 |  |
| F8A | KLRD1 |  |
| FAM58B | LTA |  |
| FNTB | CIITA |  |
| GALE | MICA |  |
| GLS | MICB |  |
| H2-K1 | NFYA |  |
| HCFC2 | NFYB |  |
| HDGFRP2 | NFYC |  |
| HEXIM1 | LGMN |  |
| HIRA | PSMC1 |  |
| HSPA13 | PSMC2 |  |
| INO80 | PSMC3 |  |
| IPPK | PSMC4 |  |
| JAGN1 | PSMC5 |  |
| JMJD6 | PSMC6 |  |
| KAT6A | PSMD1 |  |
| KLF16 | PSMD2 |  |
| KMT2A | PSMD3 |  |
| LIPT2 | PSMD4 |  |
| MED16 | PSMD5 |  |
| MED23 | PSMD7 |  |
| MED24 | PSMD8 |  |
| MGAT1 | PSMD10 |  |
| MTA2 | PSMD11 |  |
| N6AMT1 | PSMD13 |  |
| NCBP1 | PSME1 |  |
| NDUFAF6 | PSME2 |  |
| NPLOC4 | RELB |  |
| NUP188 | RFX5 |  |
| PCBP2 | RFXAP |  |
| PCED1B | SLC10A2 |  |
| PDCD6IP | THBS1 |  |
| PDSS2 | SEM1 |  |
| PI4KB | KLRC4 |  |
| PIGK | AP3B1 |  |
| PIGT | RFXANK |  |
| PKN2 | PSMD6 |  |
| PPP1CA | PSME3 |  |
| PPP1R8 | PSMD14 |  |
| PPP2R3C | CLEC4M |  |
| PRKCSH | IFI30 |  |
| PSMB9 | PROCR |  |
| PSMG1 | ADRM1 |  |
| PTAR1 | ECPAS |  |
| RCE1 | TRPC4AP |  |
| RGP1 | CD209 |  |
| RIC1 | UBXN1 |  |
| S100PBP | TAPBPL |  |
| SARNP | KIR2DL5A |  |
| SETD1A | ERAP2 |  |
| SETDB1 | ULBP3 |  |
| SLC25A32 | ULBP2 |  |
| SPCS1 | ULBP1 |  |
| SRRT | KIR3DL3 |  |
| SRSF7 | RAET1E |  |
| STOML2 | RAET1L |  |
| SUSD6 | UBR1 |  |
| TMEM208 | RAET1G |  |
| TRADD | PDIA2 |  |
| TRPM7 | HAMP |  |
| UBR5 | PI3 |  |
| UFC1 | CAMP |  |
| UFL1 | DEFB4A |  |
| USP7 | PPBP |  |
| UXS1 | REG3G |  |
| VPS13A | CXCL14 |  |
| VPS16 | CXCL16 |  |
| WDR7 | SLPI |  |
| WDR83 | CXCL8 |  |
| WWP2 | CXCL10 |  |
| YAP1 | CXCL9 |  |
| ZC3H3 | CXCL5 |  |
|  | CXCL11 |  |
|  | CXCL6 |  |
|  | CXCL1 |  |
|  | CXCL12 |  |
|  | CXCL13 |  |
|  | CXCL2 |  |
|  | PF4 |  |
|  | XCL1 |  |
|  | CXCL3 |  |
|  | DEFB103B |  |
|  | CCL13 |  |
|  | CCL1 |  |
|  | DEFB1 |  |
|  | CCL8 |  |
|  | ELANE |  |
|  | DEFB103A |  |
|  | DEFA3 |  |
|  | DEFA1 |  |
|  | TMSB10 |  |
|  | DEFA6 |  |
|  | DEFA5 |  |
|  | DEFA4 |  |
|  | LCN2 |  |
|  | LCN1 |  |
|  | COLEC10 |  |
|  | BPI |  |
|  | S100A9 |  |
|  | S100A8 |  |
|  | DCD |  |
|  | LCN6 |  |
|  | S100A12 |  |
|  | HTN3 |  |
|  | LCN8 |  |
|  | DEFA1B |  |
|  | CCR10 |  |
|  | CELA1 |  |
|  | DEFB106A |  |
|  | PENK |  |
|  | BPIFC |  |
|  | MMP12 |  |
|  | BPIFB6 |  |
|  | LEAP2 |  |
|  | SFTPD |  |
|  | LCN9 |  |
|  | BPIFB2 |  |
|  | PTGDS |  |
|  | TMSB4X |  |
|  | PGLYRP1 |  |
|  | ZC3HAV1 |  |
|  | TMSB15A |  |
|  | S100B |  |
|  | S100A13 |  |
|  | S100A6 |  |
|  | DEFB119 |  |
|  | DEFB107A |  |
|  | DEFB105A |  |
|  | SERPIND1 |  |
|  | DEFB129 |  |
|  | DEFB127 |  |
|  | S100P |  |
|  | S100A7 |  |
|  | DEFB104A |  |
|  | DEFB126 |  |
|  | DEFB106B |  |
|  | DEFB104B |  |
|  | DEFB107B |  |
|  | PGLYRP3 |  |
|  | PGLYRP2 |  |
|  | S100A10 |  |
|  | S100A2 |  |
|  | DEFB125 |  |
|  | DEFB123 |  |
|  | DEFB105B |  |
|  | DEFB132 |  |
|  | BPIFB3 |  |
|  | LCN12 |  |
|  | PGLYRP4 |  |
|  | S100A11 |  |
|  | S100A5 |  |
|  | S100A3 |  |
|  | S100A1 |  |
|  | DEFB128 |  |
|  | DEFB108B |  |
|  | HTN1 |  |
|  | LMBR1L |  |
|  | S100A7A |  |
|  | DEFB118 |  |
|  | COLEC12 |  |
|  | TMSB4Y |  |
|  | DEFB131A |  |
|  | DEFB134 |  |
|  | DEFB130A |  |
|  | DEFB124 |  |
|  | DEFB121 |  |
|  | DEFB116 |  |
|  | DEFB115 |  |
|  | DEFB114 |  |
|  | DEFB113 |  |
|  | DEFB112 |  |
|  | DEFB110 |  |
|  | TMSB15B |  |
|  | DEFB133 |  |
|  | S100Z |  |
|  | MAVS |  |
|  | TMSB4XP8 |  |
|  | S100A14 |  |
|  | LCN10 |  |
|  | S100A16 |  |
|  | DEFB136 |  |
|  | DEFB135 |  |
|  | DEFB117 |  |
|  | ZC3HAV1L |  |
|  | S100A7L2 |  |
|  | MBL3P |  |
|  | DEFB4B |  |
|  | BPIFB4 |  |
|  | AZU1 |  |
|  | DEFB131B |  |
|  | DEFA1A3 |  |
|  | LCN1P1 |  |
|  | S100G |  |
|  | DEFA7P |  |
|  | DEFB130B |  |
|  | DEFB108F |  |
|  | DEFB131C |  |
|  | TCHHL1 |  |
|  | TINAGL1 |  |
|  | SLC22A17 |  |
|  | WFIKKN1 |  |
|  | WFDC2 |  |
|  | IL6 |  |
|  | UMODL1 |  |
|  | TGFB1 |  |
|  | PF4V1 |  |
|  | MMP9 |  |
|  | ANOS1 |  |
|  | TLR4 |  |
|  | SPAG11B |  |
|  | A2M |  |
|  | NFKB1 |  |
|  | APOBEC3G |  |
|  | FABP6 |  |
|  | NOD2 |  |
|  | MBL2 |  |
|  | SFTPA1 |  |
|  | RBP1 |  |
|  | TLR2 |  |
|  | SLC40A1 |  |
|  | PLAU |  |
|  | IL1B |  |
|  | PAEP |  |
|  | HJV |  |
|  | MUC5AC |  |
|  | OBP2A |  |
|  | PLTP |  |
|  | MX1 |  |
|  | DDX58 |  |
|  | IFNL1 |  |
|  | IRF3 |  |
|  | SFTPA2 |  |
|  | LPA |  |
|  | LBP |  |
|  | RBP4 |  |
|  | NOX4 |  |
|  | LTF |  |
|  | IFNB1 |  |
|  | RBP5 |  |
|  | FABP7 |  |
|  | FABP5 |  |
|  | FABP3 |  |
|  | FABP2 |  |
|  | FABP4 |  |
|  | R3HDML |  |
|  | BPIFA3 |  |
|  | BPIFB1 |  |
|  | OASL |  |
|  | CRABP2 |  |
|  | CRABP1 |  |
|  | RBP7 |  |
|  | DUOX1 |  |
|  | OBP2B |  |
|  | RBP2 |  |
|  | LCN15 |  |
|  | CETP |  |
|  | FABP12 |  |
|  | FABP9 |  |
|  | BPIFA1 |  |
|  | LCNL1 |  |
|  | C8G |  |
|  | SPAG11A |  |
|  | PI15 |  |
|  | NOX1 |  |
|  | PMP2 |  |
|  | APOD |  |
|  | ORM2 |  |
|  | ORM1 |  |
|  | TNF |  |
|  | CTSG |  |
|  | PRTN3 |  |
|  | PML |  |
|  | AEN |  |
|  | CYBB |  |
|  | BPIFA2 |  |
|  | ISG20 |  |
|  | BCL3 |  |
|  | ISG20L2 |  |
|  | NOX5 |  |
|  | NOX3 |  |
|  | DUOX2 |  |
|  | TLR3 |  |
|  | IFIH1 |  |
|  | LRP1 |  |
|  | TRIM5 |  |
|  | IDO1 |  |
|  | GDF15 |  |
|  | NEDD4 |  |
|  | ADIPOQ |  |
|  | STAT3 |  |
|  | IFNL2 |  |
|  | SOCS3 |  |
|  | SEMG1 |  |
|  | TNFSF10 |  |
|  | CCL20 |  |
|  | RNASEL |  |
|  | IL15 |  |
|  | APOBEC3F |  |
|  | PLAAT4 |  |
|  | CHIT1 |  |
|  | CD40 |  |
|  | TLR7 |  |
|  | PPIA |  |
|  | ZYX |  |
|  | NLRX1 |  |
|  | PGC |  |
|  | VEGFA |  |
|  | IKBKE |  |
|  | ISG15 |  |
|  | DHX58 |  |
|  | TFR2 |  |
|  | FCN2 |  |
|  | MUC4 |  |
|  | F2R |  |
|  | ELN |  |
|  | IL27 |  |
|  | MAPT |  |
|  | LYZ |  |
|  | CCL5 |  |
|  | LEP |  |
|  | CYLD |  |
|  | KLKB1 |  |
|  | CST4 |  |
|  | CSRP1 |  |
|  | MAPK14 |  |
|  | JUN |  |
|  | ITGAV |  |
|  | IRF5 |  |
|  | CCR6 |  |
|  | IL12B |  |
|  | TLR8 |  |
|  | GNLY |  |
|  | CD81 |  |
|  | EIF2AK2 |  |
|  | APOM |  |
|  | CACYBP |  |
|  | NOD1 |  |
|  | MAPK8 |  |
|  | MAPK3 |  |
|  | BST2 |  |
|  | BPHL |  |
|  | PLA2G2A |  |
|  | GRN |  |
|  | NEWENTRY |  |
|  | PDGFRA |  |
|  | GNAI1 |  |
|  | WNT5A |  |
|  | FURIN |  |
|  | TYK2 |  |
|  | NOS2 |  |
|  | TRAF3 |  |
|  | TPT1 |  |
|  | TPM2 |  |
|  | NEO1 |  |
|  | AHNAK |  |
|  | TLR1 |  |
|  | TK2 |  |
|  | PRDX2 |  |
|  | MX2 |  |
|  | FGF2 |  |
|  | FGA |  |
|  | TCF7L2 |  |
|  | F2RL1 |  |
|  | TKFC |  |
|  | MSR1 |  |
|  | NFKBIZ |  |
|  | LMBR1 |  |
|  | EPPIN |  |
|  | SRC |  |
|  | MPO |  |
|  | ELAVL1 |  |
|  | ROBO3 |  |
|  | SP1 |  |
|  | SOD1 |  |
|  | PDF |  |
|  | DLL4 |  |
|  | ECD |  |
|  | SLC11A1 |  |
|  | DMBT1 |  |
|  | STING1 |  |
|  | SKIV2L |  |
|  | SEMG2 |  |
|  | DES |  |
|  | DCK |  |
|  | DAXX |  |
|  | TNFRSF10A |  |
|  | TNFRSF10B |  |
|  | EED |  |
|  | CCL4 |  |
|  | LIMS1 |  |
|  | LALBA |  |
|  | APOBEC3H |  |
|  | TMPRSS6 |  |
|  | SPINK5 |  |
|  | MARCO |  |
|  | TNFSF11 |  |
|  | KNG1 |  |
|  | CSK |  |
|  | KLRK1 |  |
|  | KCNH2 |  |
|  | JUND |  |
|  | CLDN4 |  |
|  | CCL28 |  |
|  | RNASE3 |  |
|  | RN7SL1 |  |
|  | IRF7 |  |
|  | IREB2 |  |
|  | ILK |  |
|  | IL18 |  |
|  | IL17A |  |
|  | LTB4R |  |
|  | APOBEC3A |  |
|  | MASP2 |  |
|  | TRIM27 |  |
|  | RELA |  |
|  | IL7R |  |
|  | IL1A |  |
|  | PTX3 |  |
|  | IFN1@ |  |
|  | SYTL1 |  |
|  | APOBEC3C |  |
|  | DDX17 |  |
|  | PTGS2 |  |
|  | HTR1A |  |
|  | SEPTIN7 |  |
|  | CD40LG |  |
|  | CD14 |  |
|  | MASP1 |  |
|  | PROC |  |
|  | MAP2K2 |  |
|  | MAP2K1 |  |
|  | HRG |  |
|  | NDRG1 |  |
|  | TRIM22 |  |
|  | LANCL1 |  |
|  | PPP4C |  |
|  | HMOX1 |  |
|  | HMGB1 |  |
|  | RNASE7 |  |
|  | ABCC4 |  |
|  | HGF |  |
|  | IFNLR1 |  |
|  | PLSCR1 |  |
|  | BACH2 |  |
|  | TANK |  |
|  | PIK3CG |  |
|  | ARRB1 |  |
|  | RSAD2 |  |
|  | STAB2 |  |
|  | PDYN |  |
|  | PDGFRB |  |
|  | PDCD1 |  |
|  | PCSK2 |  |
|  | PCSK1 |  |
|  | ARG2 |  |
|  | AQP9 |  |
|  | FASLG |  |
|  | APOH |  |
|  | BIRC5 |  |
|  | ANXA6 |  |
|  | IL22 |  |
|  | VTN |  |
|  | VIM |  |
|  | VCAM1 |  |
|  | PRDX1 |  |
|  | GFAP |  |
|  | GBP2 |  |
|  | ALB |  |
|  | SLC29A3 |  |
|  | OAS1 |  |
|  | AGER |  |
|  | UNC93B1 |  |
|  | TNFSF4 |  |
|  | NOS1 |  |
|  | ACTG1 |  |
|  | ACTA1 |  |
|  | ACO1 |  |
|  | SERPINA3 |  |
|  | CXCR1 |  |
|  | CCL15 |  |
|  | CCL14 |  |
|  | CCL16 |  |
|  | CCL19 |  |
|  | CCL18 |  |
|  | CCL17 |  |
|  | CCL26 |  |
|  | CCL22 |  |
|  | CCR3 |  |
|  | CCL4L1 |  |
|  | ACKR2 |  |
|  | CCR7 |  |
|  | CCL27 |  |
|  | CCR8 |  |
|  | ACKR4 |  |
|  | CCL2 |  |
|  | CCL21 |  |
|  | CCL7 |  |
|  | CCL3 |  |
|  | CCL11 |  |
|  | CCR5 |  |
|  | CCL23 |  |
|  | CCL25 |  |
|  | CCL3L3 |  |
|  | CCL4L2 |  |
|  | CCL3L1 |  |
|  | CCR1 |  |
|  | CCL24 |  |
|  | XCL2 |  |
|  | CXCR4 |  |
|  | CXCR6 |  |
|  | CCR4 |  |
|  | TAFA5 |  |
|  | TAFA3 |  |
|  | TAFA4 |  |
|  | TAFA1 |  |
|  | TAFA2 |  |
|  | CCL15-CCL14 |  |
|  | PTK2B |  |
|  | IL4 |  |
|  | CDH1 |  |
|  | LTBP1 |  |
|  | IL13 |  |
|  | IL10 |  |
|  | IL2 |  |
|  | PPARG |  |
|  | FGR |  |
|  | MIF |  |
|  | CRP |  |
|  | PTK2 |  |
|  | PTGDR |  |
|  | CD86 |  |
|  | HCK |  |
|  | VDR |  |
|  | OLR1 |  |
|  | GRK2 |  |
|  | TXK |  |
|  | RNASE2 |  |
|  | CD79A |  |
|  | CD79B |  |
|  | LYN |  |
|  | SYK |  |
|  | BTK |  |
|  | BLNK |  |
|  | VAV3 |  |
|  | VAV1 |  |
|  | VAV2 |  |
|  | RAC1 |  |
|  | RAC2 |  |
|  | RAC3 |  |
|  | PPP3CA |  |
|  | PPP3CB |  |
|  | PPP3CC |  |
|  | CHP1 |  |
|  | PPP3R1 |  |
|  | PPP3R2 |  |
|  | CHP2 |  |
|  | NFAT5 |  |
|  | NFATC1 |  |
|  | NFATC2 |  |
|  | NFATC3 |  |
|  | NFATC4 |  |
|  | HRAS |  |
|  | KRAS |  |
|  | NRAS |  |
|  | FOS |  |
|  | CARD11 |  |
|  | BCL10 |  |
|  | MALT1 |  |
|  | CHUK |  |
|  | NFKBIA |  |
|  | NFKBIB |  |
|  | NFKBIE |  |
|  | CD19 |  |
|  | CR2 |  |
|  | PIK3R5 |  |
|  | PIK3R1 |  |
|  | PIK3R2 |  |
|  | PIK3R3 |  |
|  | PIK3CA |  |
|  | PIK3CB |  |
|  | PIK3CD |  |
|  | AKT3 |  |
|  | AKT1 |  |
|  | AKT2 |  |
|  | GSK3B |  |
|  | INPP5D |  |
|  | CD22 |  |
|  | CD72 |  |
|  | PTPN6 |  |
|  | LILRB3 |  |
|  | FCGR2B |  |
|  | RASGRP3 |  |
|  | PLCG2 |  |
|  | PRKCB |  |
|  | IFITM1 |  |
|  | IGH |  |
|  | IGHA1 |  |
|  | IGHA2 |  |
|  | IGHD |  |
|  | IGHD1-1 |  |
|  | IGHD1-14 |  |
|  | IGHD1-20 |  |
|  | IGHD1-26 |  |
|  | IGHD1-7 |  |
|  | IGHD2-15 |  |
|  | IGHD2-2 |  |
|  | IGHD2-21 |  |
|  | IGHD2-8 |  |
|  | IGHD3-10 |  |
|  | IGHD3-16 |  |
|  | IGHD3-22 |  |
|  | IGHD3-3 |  |
|  | IGHD3-9 |  |
|  | IGHD4-11 |  |
|  | IGHD4-17 |  |
|  | IGHD4-23 |  |
|  | IGHD4-4 |  |
|  | IGHD5-12 |  |
|  | IGHD5-18 |  |
|  | IGHD5-24 |  |
|  | IGHD5-5 |  |
|  | IGHD6-13 |  |
|  | IGHD6-19 |  |
|  | IGHD6-25 |  |
|  | IGHD6-6 |  |
|  | IGHD7-27 |  |
|  | IGHE |  |
|  | IGHG1 |  |
|  | IGHG2 |  |
|  | IGHG3 |  |
|  | IGHG4 |  |
|  | IGHJ1 |  |
|  | IGHJ2 |  |
|  | IGHJ3 |  |
|  | IGHJ4 |  |
|  | IGHJ5 |  |
|  | IGHJ6 |  |
|  | IGHM |  |
|  | IGHV1-18 |  |
|  | IGHV1-2 |  |
|  | IGHV1-24 |  |
|  | IGHV1-3 |  |
|  | IGHV1-45 |  |
|  | IGHV1-46 |  |
|  | IGHV1-58 |  |
|  | IGHV1-69 |  |
|  | IGHV1-8 |  |
|  | IGHV1-38-4 |  |
|  | IGHV1-69-2 |  |
|  | IGHV2-26 |  |
|  | IGHV2-5 |  |
|  | IGHV2-70 |  |
|  | IGHV3-11 |  |
|  | IGHV3-13 |  |
|  | IGHV3-15 |  |
|  | IGHV3-16 |  |
|  | IGHV3-20 |  |
|  | IGHV3-21 |  |
|  | IGHV3-23 |  |
|  | IGHV3-30 |  |
|  | IGHV3-30-3 |  |
|  | IGHV3-30-5 |  |
|  | IGHV3-33 |  |
|  | IGHV3-35 |  |
|  | IGHV3-38 |  |
|  | IGHV3-43 |  |
|  | IGHV3-48 |  |
|  | IGHV3-49 |  |
|  | IGHV3-53 |  |
|  | IGHV3-64 |  |
|  | IGHV3-66 |  |
|  | IGHV3-7 |  |
|  | IGHV3-72 |  |
|  | IGHV3-73 |  |
|  | IGHV3-74 |  |
|  | IGHV3-9 |  |
|  | IGHV3-38-3 |  |
|  | IGHV3-69-1 |  |
|  | IGHV4-28 |  |
|  | IGHV4-30-1 |  |
|  | IGHV4-30-2 |  |
|  | IGHV4-30-4 |  |
|  | IGHV4-31 |  |
|  | IGHV4-34 |  |
|  | IGHV4-39 |  |
|  | IGHV4-4 |  |
|  | IGHV4-59 |  |
|  | IGHV4-61 |  |
|  | IGHV4-38-2 |  |
|  | IGHV5-51 |  |
|  | IGHV5-10-1 |  |
|  | IGHV6-1 |  |
|  | IGHV7-4-1 |  |
|  | IGHV7-81 |  |
|  | IGK |  |
|  | IGKC |  |
|  | IGKDEL |  |
|  | IGKJ |  |
|  | IGKJ1 |  |
|  | IGKJ2 |  |
|  | IGKJ3 |  |
|  | IGKJ4 |  |
|  | IGKJ5 |  |
|  | IGKV@ |  |
|  | IGKV1-12 |  |
|  | IGKV1-13 |  |
|  | IGKV1-16 |  |
|  | IGKV1-17 |  |
|  | IGKV1-27 |  |
|  | IGKV1-33 |  |
|  | IGKV1-37 |  |
|  | IGKV1-39 |  |
|  | IGKV1-5 |  |
|  | IGKV1-6 |  |
|  | IGKV1-8 |  |
|  | IGKV1-9 |  |
|  | IGKV1D-12 |  |
|  | IGKV1D-13 |  |
|  | IGKV1D-16 |  |
|  | IGKV1D-17 |  |
|  | IGKV1D-33 |  |
|  | IGKV1D-37 |  |
|  | IGKV1D-39 |  |
|  | IGKV1D-42 |  |
|  | IGKV1D-43 |  |
|  | IGKV1D-8 |  |
|  | IGKV2-24 |  |
|  | IGKV2-28 |  |
|  | IGKV2-30 |  |
|  | IGKV2-40 |  |
|  | IGKV2D-24 |  |
|  | IGKV2D-28 |  |
|  | IGKV2D-29 |  |
|  | IGKV2D-30 |  |
|  | IGKV2D-40 |  |
|  | IGKV3-11 |  |
|  | IGKV3-15 |  |
|  | IGKV3-20 |  |
|  | IGKV3-7 |  |
|  | IGKV3D-11 |  |
|  | IGKV3D-15 |  |
|  | IGKV3D-20 |  |
|  | IGKV3D-7 |  |
|  | IGKV4-1 |  |
|  | IGKV5-2 |  |
|  | IGKV6-21 |  |
|  | IGKV6D-21 |  |
|  | IGKV6D-41 |  |
|  | IGL |  |
|  | IGLC1 |  |
|  | IGLC2 |  |
|  | IGLC3 |  |
|  | IGLC6 |  |
|  | IGLC7 |  |
|  | IGLJ |  |
|  | IGLJ1 |  |
|  | IGLJ2 |  |
|  | IGLJ3 |  |
|  | IGLJ4 |  |
|  | IGLJ5 |  |
|  | IGLJ6 |  |
|  | IGLJ7 |  |
|  | IGLV@ |  |
|  | IGLV1-36 |  |
|  | IGLV1-40 |  |
|  | IGLV1-44 |  |
|  | IGLV1-47 |  |
|  | IGLV1-50 |  |
|  | IGLV1-51 |  |
|  | IGLV10-54 |  |
|  | IGLV11-55 |  |
|  | IGLV2-11 |  |
|  | IGLV2-14 |  |
|  | IGLV2-18 |  |
|  | IGLV2-23 |  |
|  | IGLV2-33 |  |
|  | IGLV2-8 |  |
|  | IGLV3-1 |  |
|  | IGLV3-10 |  |
|  | IGLV3-12 |  |
|  | IGLV3-16 |  |
|  | IGLV3-19 |  |
|  | IGLV3-21 |  |
|  | IGLV3-22 |  |
|  | IGLV3-25 |  |
|  | IGLV3-27 |  |
|  | IGLV3-32 |  |
|  | IGLV3-9 |  |
|  | IGLV4-3 |  |
|  | IGLV4-60 |  |
|  | IGLV4-69 |  |
|  | IGLV5-37 |  |
|  | IGLV5-39 |  |
|  | IGLV5-45 |  |
|  | IGLV5-48 |  |
|  | IGLV5-52 |  |
|  | IGLV6-57 |  |
|  | IGLV7-43 |  |
|  | IGLV7-46 |  |
|  | IGLV8-61 |  |
|  | IGLV9-49 |  |
|  | C3 |  |
|  | C5 |  |
|  | CCL3P1 |  |
|  | CKLF |  |
|  | CMA1 |  |
|  | CX3CL1 |  |
|  | CXCL17 |  |
|  | CCN1 |  |
|  | EDN1 |  |
|  | EDN2 |  |
|  | EDN3 |  |
|  | FGF10 |  |
|  | LECT2 |  |
|  | PPBPP1 |  |
|  | PROK2 |  |
|  | SAA1 |  |
|  | SAA2 |  |
|  | SBDS |  |
|  | SEMA3A |  |
|  | SEMA3B |  |
|  | SEMA3C |  |
|  | SEMA3D |  |
|  | SEMA3E |  |
|  | SEMA3F |  |
|  | SEMA3G |  |
|  | SEMA4A |  |
|  | SEMA4B |  |
|  | SEMA4C |  |
|  | SEMA4D |  |
|  | SEMA4F |  |
|  | SEMA4G |  |
|  | SEMA5A |  |
|  | SEMA5B |  |
|  | SEMA6A |  |
|  | SEMA6B |  |
|  | SEMA6C |  |
|  | SEMA6D |  |
|  | SEMA7A |  |
|  | SLIT1 |  |
|  | SLIT2 |  |
|  | TNC |  |
|  | TYMP |  |
|  | C5AR1 |  |
|  | CCR9 |  |
|  | CCRL2 |  |
|  | CMKLR1 |  |
|  | CX3CR1 |  |
|  | CXCR3 |  |
|  | CXCR5 |  |
|  | ACKR3 |  |
|  | CYSLTR1 |  |
|  | CYSLTR2 |  |
|  | ACKR1 |  |
|  | EDNRA |  |
|  | EDNRB |  |
|  | FPR1 |  |
|  | FPR2 |  |
|  | GPR17 |  |
|  | GPR32 |  |
|  | GPR33 |  |
|  | PTGDR2 |  |
|  | C5AR2 |  |
|  | CXCR2 |  |
|  | LTB4R2 |  |
|  | PLAUR |  |
|  | PLXNA1 |  |
|  | PLXNA2 |  |
|  | PLXNA3 |  |
|  | PLXNA4 |  |
|  | PLXNB1 |  |
|  | PLXNB2 |  |
|  | PLXNB3 |  |
|  | PLXNC1 |  |
|  | PLXND1 |  |
|  | PTAFR |  |
|  | ROBO1 |  |
|  | ROBO2 |  |
|  | RXFP3 |  |
|  | XCR1 |  |
|  | ADM |  |
|  | ADM2 |  |
|  | AGRP |  |
|  | AGT |  |
|  | AMBN |  |
|  | AMELX |  |
|  | AMH |  |
|  | ANGPTL5 |  |
|  | ANGPTL7 |  |
|  | APLN |  |
|  | AREG |  |
|  | MANF |  |
|  | CDNF |  |
|  | ARTN |  |
|  | AVP |  |
|  | BDNF |  |
|  | BMP1 |  |
|  | BMP10 |  |
|  | BMP15 |  |
|  | BMP2 |  |
|  | BMP3 |  |
|  | BMP4 |  |
|  | BMP5 |  |
|  | BMP6 |  |
|  | BMP7 |  |
|  | BMP8A |  |
|  | BMP8B |  |
|  | BTC |  |
|  | MYDGF |  |
|  | CALCA |  |
|  | CALCB |  |
|  | CAT |  |
|  | CCK |  |
|  | CD320 |  |
|  | CD70 |  |
|  | ADA2 |  |
|  | CER1 |  |
|  | CGA |  |
|  | CGB3 |  |
|  | CGB1 |  |
|  | CGB2 |  |
|  | CGB5 |  |
|  | CGB7 |  |
|  | CGB8 |  |
|  | CHGA |  |
|  | CHGB |  |
|  | CLCF1 |  |
|  | CLEC11A |  |
|  | CMTM1 |  |
|  | CMTM2 |  |
|  | CMTM3 |  |
|  | CMTM4 |  |
|  | CMTM5 |  |
|  | CMTM6 |  |
|  | CMTM7 |  |
|  | CMTM8 |  |
|  | CNTF |  |
|  | CORT |  |
|  | CRH |  |
|  | CSF1 |  |
|  | CSF2 |  |
|  | CSF3 |  |
|  | CSH1 |  |
|  | CSH2 |  |
|  | CSHL1 |  |
|  | CSPG5 |  |
|  | CTF1 |  |
|  | CCN2 |  |
|  | DKK1 |  |
|  | EBI3 |  |
|  | EGF |  |
|  | EPGN |  |
|  | EPO |  |
|  | EREG |  |
|  | ESM1 |  |
|  | FAM3B |  |
|  | FAM3C |  |
|  | FAM3D |  |
|  | FGF1 |  |
|  | FGF11 |  |
|  | FGF12 |  |
|  | FGF13 |  |
|  | FGF14 |  |
|  | FGF16 |  |
|  | FGF17 |  |
|  | FGF18 |  |
|  | FGF19 |  |
|  | FGF20 |  |
|  | FGF21 |  |
|  | FGF22 |  |
|  | FGF23 |  |
|  | FGF3 |  |
|  | FGF4 |  |
|  | FGF5 |  |
|  | FGF6 |  |
|  | FGF7 |  |
|  | FGF8 |  |
|  | FGF9 |  |
|  | VEGFD |  |
|  | FIGNL2 |  |
|  | FLT3LG |  |
|  | FSHB |  |
|  | GAL |  |
|  | GALP |  |
|  | GAST |  |
|  | GCG |  |
|  | GDF1 |  |
|  | GDF10 |  |
|  | GDF11 |  |
|  | GDF2 |  |
|  | GDF3 |  |
|  | GDF5 |  |
|  | GDF6 |  |
|  | GDF7 |  |
|  | GDF9 |  |
|  | GDNF |  |
|  | GH1 |  |
|  | GH2 |  |
|  | GHRH |  |
|  | GHRL |  |
|  | GIP |  |
|  | GKN1 |  |
|  | GMFB |  |
|  | GMFG |  |
|  | GNRH1 |  |
|  | GNRH2 |  |
|  | GPHA2 |  |
|  | GPHB5 |  |
|  | GPI |  |
|  | GREM1 |  |
|  | GREM2 |  |
|  | GRP |  |
|  | GUCA2A |  |
|  | HBEGF |  |
|  | HDGF |  |
|  | HDGFL3 |  |
|  | IAPP |  |
|  | IFNE |  |
|  | IFNK |  |
|  | IFNW1 |  |
|  | IGF1 |  |
|  | IGF2 |  |
|  | IL11 |  |
|  | IL12A |  |
|  | IL16 |  |
|  | IL17B |  |
|  | IL17C |  |
|  | IL17D |  |
|  | IL17F |  |
|  | IL19 |  |
|  | IL1F10 |  |
|  | IL36RN |  |
|  | IL36A |  |
|  | IL37 |  |
|  | IL36B |  |
|  | IL36G |  |
|  | IL1RN |  |
|  | IL20 |  |
|  | IL21 |  |
|  | IL23A |  |
|  | IL24 |  |
|  | IL25 |  |
|  | IL26 |  |
|  | IFNL3 |  |
|  | IL3 |  |
|  | IL31 |  |
|  | IL32 |  |
|  | IL33 |  |
|  | IL34 |  |
|  | IL5 |  |
|  | IL6ST |  |
|  | IL7 |  |
|  | IL9 |  |
|  | INHA |  |
|  | INHBA |  |
|  | INHBB |  |
|  | INHBC |  |
|  | INHBE |  |
|  | INS |  |
|  | INS-IGF2 |  |
|  | INSL3 |  |
|  | INSL4 |  |
|  | INSL5 |  |
|  | INSL6 |  |
|  | JAG1 |  |
|  | JAG2 |  |
|  | FGF7P6 |  |
|  | FGF7P3 |  |
|  | KITLG |  |
|  | KL |  |
|  | LACRT |  |
|  | LEFTY1 |  |
|  | LEFTY2 |  |
|  | LHB |  |
|  | LIF |  |
|  | LRSAM1 |  |
|  | LTB |  |
|  | LTBP2 |  |
|  | LTBP3 |  |
|  | LTBP4 |  |
|  | MDK |  |
|  | MIA |  |
|  | MLN |  |
|  | MSTN |  |
|  | NAMPT |  |
|  | NDP |  |
|  | NENF |  |
|  | NGF |  |
|  | NMB |  |
|  | NODAL |  |
|  | CCN3 |  |
|  | NPFF |  |
|  | NPPA |  |
|  | NPPB |  |
|  | NPPC |  |
|  | NPY |  |
|  | NRG1 |  |
|  | NRG2 |  |
|  | NRG3 |  |
|  | NRG4 |  |
|  | NRTN |  |
|  | NTF3 |  |
|  | NTF4 |  |
|  | NTS |  |
|  | NUDT6 |  |
|  | OGN |  |
|  | OSGIN1 |  |
|  | OSM |  |
|  | OSTN |  |
|  | OXT |  |
|  | ENDOU |  |
|  | PDGFA |  |
|  | PDGFB |  |
|  | PDGFC |  |
|  | PDGFD |  |
|  | PDGFRL |  |
|  | PGF |  |
|  | PMCH |  |
|  | PNOC |  |
|  | POMC |  |
|  | PPBPP2 |  |
|  | PPY |  |
|  | PRL |  |
|  | PRLH |  |
|  | PROK1 |  |
|  | PSPN |  |
|  | PTH |  |
|  | PTH2 |  |
|  | PTHLH |  |
|  | PTN |  |
|  | PYY |  |
|  | QRFP |  |
|  | RABEP1 |  |
|  | RABEP2 |  |
|  | REG1A |  |
|  | RETN |  |
|  | RETNLB |  |
|  | RLN1 |  |
|  | RLN2 |  |
|  | RLN3 |  |
|  | SCG2 |  |
|  | SCGB3A1 |  |
|  | SCT |  |
|  | AIMP1 |  |
|  | SECTM1 |  |
|  | SLURP1 |  |
|  | SPP1 |  |
|  | SST |  |
|  | STC1 |  |
|  | STC2 |  |
|  | TAC1 |  |
|  | TDGF1 |  |
|  | TDGF1P3 |  |
|  | TG |  |
|  | TGFA |  |
|  | TGFB2 |  |
|  | TGFB3 |  |
|  | THPO |  |
|  | TNFRSF11B |  |
|  | TNFSF12 |  |
|  | TNFSF13 |  |
|  | TNFSF13B |  |
|  | TNFSF14 |  |
|  | TNFSF15 |  |
|  | TNFSF18 |  |
|  | TNFSF8 |  |
|  | TNFSF9 |  |
|  | TOR2A |  |
|  | TRH |  |
|  | TSHB |  |
|  | TSLP |  |
|  | TXLNA |  |
|  | UCN |  |
|  | UCN2 |  |
|  | UCN3 |  |
|  | UTS2 |  |
|  | UTS2B |  |
|  | VEGFB |  |
|  | VEGFC |  |
|  | VGF |  |
|  | VIP |  |
|  | ACVR1B |  |
|  | ACVR1C |  |
|  | ACVR2A |  |
|  | ACVR2B |  |
|  | ACVRL1 |  |
|  | ADCYAP1R1 |  |
|  | ADIPOR1 |  |
|  | ADIPOR2 |  |
|  | ADRB1 |  |
|  | ADRB2 |  |
|  | AGTR1 |  |
|  | AGTR2 |  |
|  | AMHR2 |  |
|  | ANGPT1 |  |
|  | ANGPT4 |  |
|  | ANGPTL1 |  |
|  | ANGPTL2 |  |
|  | ANGPTL3 |  |
|  | ANGPTL4 |  |
|  | ANGPTL6 |  |
|  | APLNR |  |
|  | AR |  |
|  | AVPR1A |  |
|  | AVPR1B |  |
|  | AVPR2 |  |
|  | BMPR1A |  |
|  | BMPR1B |  |
|  | BMPR2 |  |
|  | BRD8 |  |
|  | C3AR1 |  |
|  | CALCR |  |
|  | CALCRL |  |
|  | CNTFR |  |
|  | CRHR1 |  |
|  | CRHR2 |  |
|  | CRIM1 |  |
|  | CRLF1 |  |
|  | CRLF2 |  |
|  | CRLF3 |  |
|  | CSF1R |  |
|  | CSF2RA |  |
|  | CSF2RB |  |
|  | CSF3R |  |
|  | EGFR |  |
|  | ENG |  |
|  | EPOR |  |
|  | ESR1 |  |
|  | ESR2 |  |
|  | ESRRA |  |
|  | ESRRB |  |
|  | ESRRG |  |
|  | FGFR1 |  |
|  | FGFR2 |  |
|  | FGFR3 |  |
|  | FGFR4 |  |
|  | FGFRL1 |  |
|  | FLT1 |  |
|  | FLT3 |  |
|  | FLT4 |  |
|  | FSHR |  |
|  | GALR2 |  |
|  | GALR3 |  |
|  | GCGR |  |
|  | GHR |  |
|  | GHRHR |  |
|  | GHSR |  |
|  | GIPR |  |
|  | GLP1R |  |
|  | GLP2R |  |
|  | GNRHR |  |
|  | GPER1 |  |
|  | HNF4A |  |
|  | HNF4G |  |
|  | HTR3A |  |
|  | HTR3B |  |
|  | HTR3C |  |
|  | HTR3D |  |
|  | HTR3E |  |
|  | IGF1R |  |
|  | IGF2R |  |
|  | IL10RA |  |
|  | IL10RB |  |
|  | IL11RA |  |
|  | IL12RB1 |  |
|  | IL12RB2 |  |
|  | IL13RA1 |  |
|  | IL13RA2 |  |
|  | IL15RA |  |
|  | IL2RB |  |
|  | IL17RA |  |
|  | IL17RB |  |
|  | IL17RC |  |
|  | IL17RD |  |
|  | IL17RE |  |
|  | IL18R1 |  |
|  | IL18RAP |  |
|  | IL1R1 |  |
|  | IL1R2 |  |
|  | IL1RAP |  |
|  | IL1RL1 |  |
|  | IL1RL2 |  |
|  | IL20RA |  |
|  | IL20RB |  |
|  | IL21R |  |
|  | IL22RA1 |  |
|  | IL22RA2 |  |
|  | IL23R |  |
|  | IL27RA |  |
|  | IL2RA |  |
|  | IL2RG |  |
|  | IL31RA |  |
|  | IL3RA |  |
|  | IL4R |  |
|  | IL5RA |  |
|  | IL6R |  |
|  | IL9R |  |
|  | INSR |  |
|  | KDR |  |
|  | LEPR |  |
|  | LGR4 |  |
|  | LGR5 |  |
|  | LGR6 |  |
|  | LHCGR |  |
|  | LIFR |  |
|  | LTBR |  |
|  | MC1R |  |
|  | MC2R |  |
|  | MC3R |  |
|  | MC4R |  |
|  | MCHR1 |  |
|  | MCHR2 |  |
|  | MET |  |
|  | MLNR |  |
|  | MPL |  |
|  | MTNR1A |  |
|  | MTNR1B |  |
|  | NGFR |  |
|  | NMBR |  |
|  | NPR1 |  |
|  | NPR3 |  |
|  | NR0B1 |  |
|  | NR0B2 |  |
|  | NR1D1 |  |
|  | NR1D2 |  |
|  | NR1H2 |  |
|  | NR1H3 |  |
|  | NR1H4 |  |
|  | NR1I2 |  |
|  | NR1I3 |  |
|  | NR2C1 |  |
|  | NR2C2 |  |
|  | NR2E1 |  |
|  | NR2E3 |  |
|  | NR2F1 |  |
|  | NR2F2 |  |
|  | NR2F6 |  |
|  | NR3C1 |  |
|  | NR3C2 |  |
|  | NR4A1 |  |
|  | NR4A2 |  |
|  | NR4A3 |  |
|  | NR5A1 |  |
|  | NR5A2 |  |
|  | NR6A1 |  |
|  | NRP1 |  |
|  | NRP2 |  |
|  | OGFR |  |
|  | OPRD1 |  |
|  | OPRK1 |  |
|  | OPRL1 |  |
|  | OPRM1 |  |
|  | OSMR |  |
|  | OXTR |  |
|  | PGR |  |
|  | PGRMC2 |  |
|  | PPARA |  |
|  | PPARD |  |
|  | PRLHR |  |
|  | PRLR |  |
|  | PTGER1 |  |
|  | PTGER2 |  |
|  | PTGER3 |  |
|  | PTGER4 |  |
|  | PTGFR |  |
|  | PTH1R |  |
|  | PTH2R |  |
|  | RARA |  |
|  | RARB |  |
|  | RARG |  |
|  | RORA |  |
|  | RORB |  |
|  | RORC |  |
|  | RXFP1 |  |
|  | RXFP2 |  |
|  | RXRA |  |
|  | RXRB |  |
|  | RXRG |  |
|  | S1PR1 |  |
|  | S1PR2 |  |
|  | SCTR |  |
|  | SDC1 |  |
|  | SDC2 |  |
|  | SDC3 |  |
|  | SDC4 |  |
|  | SORT1 |  |
|  | SSTR1 |  |
|  | SSTR2 |  |
|  | SSTR5 |  |
|  | ST2 |  |
|  | TACR1 |  |
|  | TEK |  |
|  | TGFBR1 |  |
|  | TGFBR3 |  |
|  | THRA |  |
|  | THRB |  |
|  | TIE1 |  |
|  | TNFRSF10C |  |
|  | TNFRSF10D |  |
|  | TNFRSF11A |  |
|  | TNFRSF12A |  |
|  | TNFRSF13B |  |
|  | TNFRSF13C |  |
|  | TNFRSF14 |  |
|  | TNFRSF17 |  |
|  | TNFRSF18 |  |
|  | TNFRSF19 |  |
|  | TNFRSF21 |  |
|  | TNFRSF25 |  |
|  | TNFRSF4 |  |
|  | TNFRSF6B |  |
|  | TNFRSF8 |  |
|  | TNFRSF9 |  |
|  | TRHR |  |
|  | TSHR |  |
|  | TUBB3 |  |
|  | VIPR1 |  |
|  | VIPR2 |  |
|  | PTPN11 |  |
|  | ICAM2 |  |
|  | ITGAL |  |
|  | ITGB2 |  |
|  | PAK1 |  |
|  | NCR2 |  |
|  | TYROBP |  |
|  | LCK |  |
|  | FCGR3A |  |
|  | FCGR3B |  |
|  | NCR1 |  |
|  | NCR3 |  |
|  | CD247 |  |
|  | ZAP70 |  |
|  | LCP2 |  |
|  | LAT |  |
|  | PLCG1 |  |
|  | SH3BP2 |  |
|  | FYN |  |
|  | SHC2 |  |
|  | SHC4 |  |
|  | SHC3 |  |
|  | SHC1 |  |
|  | GRB2 |  |
|  | SOS1 |  |
|  | SOS2 |  |
|  | ARAF |  |
|  | BRAF |  |
|  | RAF1 |  |
|  | HCST |  |
|  | CD48 |  |
|  | CD244 |  |
|  | PRKCA |  |
|  | PRKCG |  |
|  | SH2D1B |  |
|  | SH2D1A |  |
|  | GZMB |  |
|  | PRF1 |  |
|  | CASP3 |  |
|  | BID |  |
|  | CD3D |  |
|  | CD3E |  |
|  | CD3G |  |
|  | PTPRC |  |
|  | ITK |  |
|  | TEC |  |
|  | NCK1 |  |
|  | NCK2 |  |
|  | GRAP2 |  |
|  | PAK2 |  |
|  | PAK3 |  |
|  | PAK4 |  |
|  | PAK6 |  |
|  | PAK5 |  |
|  | RHOA |  |
|  | CDC42 |  |
|  | CD28 |  |
|  | ICOS |  |
|  | MAP3K8 |  |
|  | MAP3K14 |  |
|  | CTLA4 |  |
|  | CBLC |  |
|  | CBL |  |
|  | CBLB |  |
|  | CDK4 |  |
|  | RASGRP1 |  |
|  | PDK1 |  |
|  | PRKCQ |  |
|  | TRAC |  |
|  | TRAJ1 |  |
|  | TRAJ2 |  |
|  | TRAJ3 |  |
|  | TRAJ4 |  |
|  | TRAJ5 |  |
|  | TRAJ6 |  |
|  | TRAJ7 |  |
|  | TRAJ8 |  |
|  | TRAJ9 |  |
|  | TRAJ10 |  |
|  | TRAJ11 |  |
|  | TRAJ12 |  |
|  | TRAJ13 |  |
|  | TRAJ14 |  |
|  | TRAJ15 |  |
|  | TRAJ16 |  |
|  | TRAJ17 |  |
|  | TRAJ18 |  |
|  | TRAJ19 |  |
|  | TRAJ20 |  |
|  | TRAJ21 |  |
|  | TRAJ22 |  |
|  | TRAJ23 |  |
|  | TRAJ24 |  |
|  | TRAJ25 |  |
|  | TRAJ26 |  |
|  | TRAJ27 |  |
|  | TRAJ28 |  |
|  | TRAJ29 |  |
|  | TRAJ30 |  |
|  | TRAJ31 |  |
|  | TRAJ32 |  |
|  | TRAJ33 |  |
|  | TRAJ34 |  |
|  | TRAJ35 |  |
|  | TRAJ36 |  |
|  | TRAJ37 |  |
|  | TRAJ38 |  |
|  | TRAJ39 |  |
|  | TRAJ40 |  |
|  | TRAJ41 |  |
|  | TRAJ42 |  |
|  | TRAJ43 |  |
|  | TRAJ44 |  |
|  | TRAJ45 |  |
|  | TRAJ46 |  |
|  | TRAJ47 |  |
|  | TRAJ48 |  |
|  | TRAJ49 |  |
|  | TRAJ50 |  |
|  | TRAJ52 |  |
|  | TRAJ53 |  |
|  | TRAJ54 |  |
|  | TRAJ56 |  |
|  | TRAJ57 |  |
|  | TRAJ58 |  |
|  | TRAJ59 |  |
|  | TRAJ61 |  |
|  | TRAV1-1 |  |
|  | TRAV1-2 |  |
|  | TRAV2 |  |
|  | TRAV3 |  |
|  | TRAV4 |  |
|  | TRAV5 |  |
|  | TRAV7 |  |
|  | TRAV8-1 |  |
|  | TRAV8-2 |  |
|  | TRAV8-3 |  |
|  | TRAV8-4 |  |
|  | TRAV8-6 |  |
|  | TRAV8-7 |  |
|  | TRAV9-1 |  |
|  | TRAV9-2 |  |
|  | TRAV10 |  |
|  | TRAV12-1 |  |
|  | TRAV12-2 |  |
|  | TRAV12-3 |  |
|  | TRAV13-1 |  |
|  | TRAV13-2 |  |
|  | TRAV14DV4 |  |
|  | TRAV16 |  |
|  | TRAV17 |  |
|  | TRAV18 |  |
|  | TRAV19 |  |
|  | TRAV20 |  |
|  | TRAV21 |  |
|  | TRAV22 |  |
|  | TRAV23DV6 |  |
|  | TRAV24 |  |
|  | TRAV25 |  |
|  | TRAV26-1 |  |
|  | TRAV26-2 |  |
|  | TRAV27 |  |
|  | TRAV29DV5 |  |
|  | TRAV30 |  |
|  | TRAV34 |  |
|  | TRAV35 |  |
|  | TRAV36DV7 |  |
|  | TRAV38-1 |  |
|  | TRAV38-2DV8 |  |
|  | TRAV39 |  |
|  | TRAV40 |  |
|  | TRAV41 |  |
|  | TRBC1 |  |
|  | TRBC2 |  |
|  | TRBD1 |  |
|  | TRBD2 |  |
|  | TRBJ1-1 |  |
|  | TRBJ1-2 |  |
|  | TRBJ1-3 |  |
|  | TRBJ1-4 |  |
|  | TRBJ1-5 |  |
|  | TRBJ1-6 |  |
|  | TRBJ2-1 |  |
|  | TRBJ2-2 |  |
|  | TRBJ2-3 |  |
|  | TRBJ2-4 |  |
|  | TRBJ2-5 |  |
|  | TRBJ2-6 |  |
|  | TRBJ2-7 |  |
|  | TRBV2 |  |
|  | TRBV3-1 |  |
|  | TRBV4-1 |  |
|  | TRBV4-2 |  |
|  | TRBV4-3 |  |
|  | TRBV5-1 |  |
|  | TRBV5-4 |  |
|  | TRBV5-5 |  |
|  | TRBV5-6 |  |
|  | TRBV5-7 |  |
|  | TRBV5-8 |  |
|  | TRBV6-1 |  |
|  | TRBV6-2 |  |
|  | TRBV6-3 |  |
|  | TRBV6-4 |  |
|  | TRBV6-5 |  |
|  | TRBV6-6 |  |
|  | TRBV6-7 |  |
|  | TRBV6-8 |  |
|  | TRBV6-9 |  |
|  | TRBV7-2 |  |
|  | TRBV7-3 |  |
|  | TRBV7-4 |  |
|  | TRBV7-6 |  |
|  | TRBV7-7 |  |
|  | TRBV7-8 |  |
|  | TRBV7-9 |  |
|  | TRBV9 |  |
|  | TRBV10-1 |  |
|  | TRBV10-2 |  |
|  | TRBV10-3 |  |
|  | TRBV11-1 |  |
|  | TRBV11-2 |  |
|  | TRBV11-3 |  |
|  | TRBV12-3 |  |
|  | TRBV12-4 |  |
|  | TRBV12-5 |  |
|  | TRBV13 |  |
|  | TRBV14 |  |
|  | TRBV15 |  |
|  | TRBV16 |  |
|  | TRBV17 |  |
|  | TRBV18 |  |
|  | TRBV19 |  |
|  | TRBV20-1 |  |
|  | TRBV24-1 |  |
|  | TRBV25-1 |  |
|  | TRBV27 |  |
|  | TRBV28 |  |
|  | TRBV29-1 |  |
|  | TRBV30 |  |
|  | TRDC |  |
|  | TRDD1 |  |
|  | TRDD2 |  |
|  | TRDD3 |  |
|  | TRDJ1 |  |
|  | TRDJ2 |  |
|  | TRDJ3 |  |
|  | TRDJ4 |  |
|  | TRDV1 |  |
|  | TRDV2 |  |
|  | TRDV3 |  |
|  | TRGV9 |  |
|  | TRGV8 |  |
|  | TRGV5 |  |
|  | TRGV4 |  |
|  | TRGV3 |  |
|  | TRGV2 |  |
|  | TRGJP2 |  |
|  | TRGJP1 |  |
|  | TRGJP |  |
|  | TRGJ2 |  |
|  | TRGJ1 |  |
|  | TRGC2 |  |
|  | TRGC1 |  |
|  | TRAV6 |  |
