# Supplementary material for: Robust machine−learning based prognostic index using cytotoxic T lymphocyte evasion genes highlights potential therapeutic targets in colorectal cancer
Source: Cancer Cell Int. 2024 Jan 31;24:52. doi: 10.1186/s12935-024-03239-y (PMC10829178; doi:10.1186/s12935-024-03239-y)
Supplement: Supplementary file 1 — Additional file 1: Figure S1. (A) Expression and (B) prognostic significance of 31 core CERGs in TCGA-CRC dataset. Figure S2. IHC score of HOXC6 (A), G0S2 (B), and MX2 (C) in normal tissues and CRC. **p < 0.01; ***p < 0.001. Table S1. Published signatures applied for model comparison. Table S2. Sequences for qRT-PCR primers. Table S3. Detailed si-RNA sequences used in the study. Table S4. 182 CERGs from published research and 1793 IRGs from Immport database. Table S5. Published signatures applied for model comparison. C-index of each combination of machine learning method for developing the prognostic signature. Table S6. AUC value of each combination of machine learning method for constructing the immunotherapy-related signature. [file 12935_2024_3239_MOESM1_ESM.zip › Supplementary Material/Supplementary Table S5.docx]

**Supplementary Table S5: C-index of each combination of machine learning method for developing the prognostic signature.**

| TCGA | GSE17536 | GSE17537 | GSE29621 | GSE38832 | GSE39582 | GSE72970 |
| --- | --- | --- | --- | --- | --- | --- |
| Lasso+StepCox[both] | 0.734749239 | 0.59659671 | 0.648863636 | 0.605951307 | 0.745131245 | 0.624412245 |
| survivalSVM | 0.503473601 | 0.564946115 | 0.609090909 | 0.486925158 | 0.535563082 | 0.52346224 |
| CoxBoost+survivalSVM | 0.520444621 | 0.554736245 | 0.628409091 | 0.521190261 | 0.49491956 | 0.524750847 |
| Ridge | 0.749603017 | 0.619739081 | 0.668181818 | 0.643823264 | 0.743014395 | 0.615542791 |
| Lasso+survivalSVM | 0.530600768 | 0.568689733 | 0.631818182 | 0.503155996 | 0.519475021 | 0.531632555 |
| SuperPC | 0.582010057 | 0.566420874 | 0.646590909 | 0.524797115 | 0.542337003 | 0.547520803 |
| CoxBoost+Ridge | 0.762141061 | 0.613159387 | 0.670454545 | 0.637511271 | 0.743861135 | 0.62406953 |
| Enet[alpha=0.1] | 0.759891491 | 0.611344299 | 0.667045455 | 0.637511271 | 0.751481795 | 0.62552264 |
| CoxBoost+Enet[alpha=0.1] | 0.761347095 | 0.613159387 | 0.669318182 | 0.635707845 | 0.748941575 | 0.625303302 |
| Enet[alpha=0.2] | 0.760718539 | 0.611911514 | 0.669318182 | 0.634806132 | 0.754868755 | 0.62590648 |
| Enet[alpha=0.3] | 0.757311102 | 0.606012479 | 0.665909091 | 0.62037872 | 0.749788315 | 0.628072436 |
| CoxBoost+Enet[alpha=0.3] | 0.756682546 | 0.607146909 | 0.664772727 | 0.622182146 | 0.751058425 | 0.627729722 |
| CoxBoost+Enet[alpha=0.2] | 0.756285563 | 0.60567215 | 0.661363636 | 0.619477006 | 0.749788315 | 0.628086145 |
| Enet[alpha=0.4] | 0.755293106 | 0.605331821 | 0.664772727 | 0.621280433 | 0.751058425 | 0.62774343 |
| CoxBoost+Enet[alpha=0.4] | 0.754763795 | 0.60453772 | 0.660227273 | 0.61767358 | 0.749364945 | 0.628867534 |
| Lasso+CoxBoost | 0.749933836 | 0.600113443 | 0.664772727 | 0.6122633 | 0.749364945 | 0.630539981 |
| Enet[alpha=0.5] | 0.75363901 | 0.603289847 | 0.665909091 | 0.62037872 | 0.751481795 | 0.628387734 |
| CoxBoost | 0.753903665 | 0.603516733 | 0.664772727 | 0.614066727 | 0.751905165 | 0.628223231 |
| CoxBoost+Enet[alpha=0.5] | 0.753572846 | 0.602949518 | 0.663636364 | 0.616771867 | 0.749788315 | 0.62900462 |
| Enet[alpha=0.6] | 0.751654096 | 0.604310834 | 0.668181818 | 0.618575293 | 0.750211685 | 0.627949059 |
| CoxBoost+Enet[alpha=0.6] | 0.752514225 | 0.60226886 | 0.6625 | 0.61767358 | 0.749788315 | 0.629607797 |
| CoxBoost+Enet[alpha=0.7] | 0.750992457 | 0.601247873 | 0.663636364 | 0.615870153 | 0.749788315 | 0.629511837 |
| CoxBoost+Enet[alpha=0.8] | 0.750496229 | 0.60113443 | 0.668181818 | 0.61496844 | 0.750635055 | 0.630032764 |
| Enet[alpha=0.8] | 0.750264655 | 0.600907544 | 0.664772727 | 0.61496844 | 0.749788315 | 0.629690049 |
| Enet[alpha=0.9] | 0.749371444 | 0.6 | 0.663636364 | 0.61496844 | 0.749364945 | 0.630169849 |
| Lasso | 0.747651184 | 0.599546228 | 0.664772727 | 0.613165014 | 0.750635055 | 0.630060181 |
| Enet[alpha=0.7] | 0.750463147 | 0.602495746 | 0.668181818 | 0.616771867 | 0.750635055 | 0.629648923 |
| CoxBoost+Enet[alpha=0.9] | 0.749404526 | 0.6 | 0.663636364 | 0.61496844 | 0.749364945 | 0.630183558 |
| CoxBoost+Lasso | 0.748643642 | 0.599319342 | 0.661363636 | 0.6122633 | 0.749364945 | 0.630608524 |
| Lasso+plsRcox | 0.767202594 | 0.617016449 | 0.673863636 | 0.646528404 | 0.738357324 | 0.618353051 |
| CoxBoost+plsRcox | 0.767533413 | 0.627112876 | 0.657954545 | 0.661857529 | 0.744284505 | 0.62387761 |
| CoxBoost+StepCox[forward] | 0.747022628 | 0.598411798 | 0.648863636 | 0.607754734 | 0.744284505 | 0.631458456 |
| Lasso+StepCox[forward] | 0.750033082 | 0.601020987 | 0.646590909 | 0.610459874 | 0.743014395 | 0.631143159 |
| RSF+survivalSVM | 0.52682943 | 0.524560408 | 0.528409091 | 0.505861136 | 0.592294666 | 0.511247892 |
| CoxBoost+SuperPC | 0.552931057 | 0.553601815 | 0.6125 | 0.502254283 | 0.477561389 | 0.535731421 |
| StepCox[forward] | 0.75155485 | 0.588882587 | 0.635227273 | 0.603246168 | 0.740050804 | 0.626660452 |
| plsRcox | 0.744409157 | 0.629495179 | 0.651136364 | 0.646528404 | 0.731583404 | 0.631540708 |
| RSF+Ridge | 0.742986635 | 0.592626205 | 0.621590909 | 0.634806132 | 0.719729043 | 0.638669171 |
| RSF+Enet[alpha=0.1] | 0.742490406 | 0.591378332 | 0.620454545 | 0.633002705 | 0.714648603 | 0.637928907 |
| Lasso+SuperPC | 0.58326717 | 0.57424844 | 0.643181818 | 0.533814247 | 0.554614733 | 0.552853442 |
| RSF+plsRcox | 0.743813683 | 0.593987521 | 0.642045455 | 0.645626691 | 0.733276884 | 0.637161227 |
| RSF+StepCox[forward] | 0.737395792 | 0.588088486 | 0.610227273 | 0.614066727 | 0.707027942 | 0.633034943 |
| RSF+Enet[alpha=0.2] | 0.740869393 | 0.590470788 | 0.619318182 | 0.627592426 | 0.714648603 | 0.638024867 |
| RSF+Enet[alpha=0.3] | 0.739546116 | 0.590357345 | 0.615909091 | 0.626690712 | 0.717188823 | 0.63751765 |
| RSF+Enet[alpha=0.6] | 0.739314543 | 0.590243902 | 0.619318182 | 0.628494139 | 0.717188823 | 0.637490233 |
| RSF+Lasso | 0.737792775 | 0.590470788 | 0.617045455 | 0.626690712 | 0.715918713 | 0.636681426 |
| RSF+Enet[alpha=0.7] | 0.739810771 | 0.59092456 | 0.618181818 | 0.626690712 | 0.718458933 | 0.637558776 |
| RSF+Enet[alpha=0.5] | 0.739546116 | 0.590357345 | 0.618181818 | 0.628494139 | 0.717188823 | 0.637298312 |
| RSF+CoxBoost | 0.740373164 | 0.591038003 | 0.618181818 | 0.631199279 | 0.717188823 | 0.638833674 |
| RSF+Enet[alpha=0.9] | 0.738355167 | 0.58979013 | 0.617045455 | 0.627592426 | 0.718458933 | 0.637161227 |
| RSF+Enet[alpha=0.4] | 0.739413789 | 0.590017016 | 0.617045455 | 0.627592426 | 0.715918713 | 0.637284604 |
| RSF+Enet[alpha=0.8] | 0.738090512 | 0.590017016 | 0.615909091 | 0.626690712 | 0.716342083 | 0.636791095 |
| RSF+StepCox[both] | 0.730183935 | 0.585252411 | 0.6 | 0.600541028 | 0.705334462 | 0.630293227 |
| RSF+StepCox[backward] | 0.730183935 | 0.585252411 | 0.6 | 0.600541028 | 0.705334462 | 0.630293227 |
| StepCox[both]+Ridge | 0.738355167 | 0.588655701 | 0.65 | 0.607754734 | 0.746824725 | 0.624220324 |
| StepCox[backward]+Ridge | 0.738123594 | 0.587407828 | 0.65 | 0.608656447 | 0.747671465 | 0.624028404 |
| StepCox[both]+plsRcox | 0.742093423 | 0.593533749 | 0.628409091 | 0.621280433 | 0.739627434 | 0.620820596 |
| StepCox[backward]+plsRcox | 0.742093423 | 0.593533749 | 0.628409091 | 0.621280433 | 0.739627434 | 0.620820596 |
| StepCox[both]+Enet[alpha=0.9] | 0.733459045 | 0.586046512 | 0.652272727 | 0.603246168 | 0.745977985 | 0.622643837 |
| StepCox[backward]+Enet[alpha=0.9] | 0.73355829 | 0.585933069 | 0.648863636 | 0.603246168 | 0.745554615 | 0.623850193 |
| StepCox[both]+Enet[alpha=0.1] | 0.735344714 | 0.587180942 | 0.652272727 | 0.607754734 | 0.746401355 | 0.62368569 |
| StepCox[backward]+Enet[alpha=0.1] | 0.73580786 | 0.587294385 | 0.652272727 | 0.607754734 | 0.745131245 | 0.62387761 |
| StepCox[both]+Enet[alpha=0.8] | 0.733657536 | 0.586159955 | 0.647727273 | 0.603246168 | 0.746401355 | 0.623836484 |
| StepCox[backward]+Enet[alpha=0.8] | 0.733459045 | 0.586046512 | 0.652272727 | 0.603246168 | 0.745977985 | 0.622602712 |
| StepCox[both]+Enet[alpha=0.2] | 0.735245468 | 0.587294385 | 0.652272727 | 0.609558161 | 0.745977985 | 0.624110656 |
| StepCox[backward]+Enet[alpha=0.2] | 0.735774778 | 0.587180942 | 0.65 | 0.611361587 | 0.746401355 | 0.625207342 |
| StepCox[both]+Lasso | 0.733492127 | 0.585933069 | 0.648863636 | 0.602344454 | 0.745554615 | 0.623946153 |
| StepCox[backward]+Lasso | 0.733525208 | 0.586046512 | 0.652272727 | 0.603246168 | 0.745977985 | 0.622643837 |
| StepCox[both]+Enet[alpha=0.6] | 0.733955273 | 0.586273398 | 0.648863636 | 0.603246168 | 0.747248095 | 0.62368569 |
| StepCox[backward]+Enet[alpha=0.6] | 0.7337237 | 0.586386841 | 0.651136364 | 0.603246168 | 0.745554615 | 0.623247015 |
| CoxBoost+GBM | 0.503969829 | 0.496880318 | 0.5 | 0.508566276 | 0.5 | 0.500973309 |
| StepCox[both]+Enet[alpha=0.7] | 0.733889109 | 0.586273398 | 0.646590909 | 0.605049594 | 0.745131245 | 0.624151781 |
| StepCox[backward]+Enet[alpha=0.7] | 0.733789864 | 0.586046512 | 0.648863636 | 0.603246168 | 0.745977985 | 0.623809067 |
| Lasso+StepCox[backward] | 0.734749239 | 0.59659671 | 0.648863636 | 0.605951307 | 0.745131245 | 0.624412245 |
| StepCox[both] | 0.732598915 | 0.587521271 | 0.652272727 | 0.599639315 | 0.745131245 | 0.621478608 |
| StepCox[backward] | 0.732598915 | 0.587521271 | 0.652272727 | 0.599639315 | 0.745131245 | 0.621478608 |
| CoxBoost+StepCox[both] | 0.734749239 | 0.59659671 | 0.648863636 | 0.605951307 | 0.745131245 | 0.624412245 |
| CoxBoost+StepCox[backward] | 0.734749239 | 0.59659671 | 0.648863636 | 0.605951307 | 0.745131245 | 0.624412245 |
| StepCox[both]+Enet[alpha=0.4] | 0.735046976 | 0.587294385 | 0.647727273 | 0.608656447 | 0.742591025 | 0.624933171 |
| StepCox[backward]+Enet[alpha=0.4] | 0.735046976 | 0.587294385 | 0.647727273 | 0.608656447 | 0.742591025 | 0.624933171 |
| StepCox[both]+Enet[alpha=0.3] | 0.734914649 | 0.587634714 | 0.65 | 0.607754734 | 0.745131245 | 0.624234033 |
| StepCox[backward]+Enet[alpha=0.3] | 0.734914649 | 0.587634714 | 0.65 | 0.607754734 | 0.745131245 | 0.624234033 |
| StepCox[both]+CoxBoost | 0.73355829 | 0.586273398 | 0.65 | 0.603246168 | 0.745131245 | 0.623329266 |
| StepCox[backward]+CoxBoost | 0.733591372 | 0.586159955 | 0.645454545 | 0.604147881 | 0.743437765 | 0.625275885 |
| StepCox[both]+Enet[alpha=0.5] | 0.73355829 | 0.585933069 | 0.652272727 | 0.603246168 | 0.745554615 | 0.622465626 |
| StepCox[backward]+Enet[alpha=0.5] | 0.734451502 | 0.587067499 | 0.648863636 | 0.609558161 | 0.742591025 | 0.625179925 |
| CoxBoost+RSF | 0.502514225 | 0.487975043 | 0.517613636 | 0.484220018 | 0.502328535 | 0.511940176 |
| RSF+SuperPC | 0.493118963 | 0.53522405 | 0.557954545 | 0.491433724 | 0.576629975 | 0.500747118 |
| RSF | 0.502663094 | 0.507487238 | 0.543181818 | 0.527953111 | 0.518839966 | 0.500438675 |
| Lasso+GBM | 0.501455604 | 0.493703914 | 0.5 | 0.5 | 0.5 | 0.504441581 |
| RSF+GBM | 0.51194257 | 0.489676687 | 0.5 | 0.5 | 0.5 | 0.507772767 |
| GBM | 0.510238851 | 0.486783891 | 0.545454545 | 0.541929666 | 0.503598645 | 0.498992419 |
| StepCox[both]+survivalSVM | 0.516673283 | 0.534997164 | 0.5875 | 0.593327322 | 0.544030483 | 0.500418112 |
| StepCox[backward]+survivalSVM | 0.516673283 | 0.534997164 | 0.5875 | 0.593327322 | 0.544030483 | 0.500418112 |
| Lasso+RSF | 0.502514225 | 0.515428247 | 0.528409091 | 0.524797115 | 0.5 | 0.504798004 |
| StepCox[both]+GBM | 0.500463147 | 0.509699376 | 0.5 | 0.5 | 0.5 | 0.505695916 |
| StepCox[backward]+GBM | 0.500545852 | 0.509699376 | 0.5 | 0.5 | 0.5 | 0.503118703 |
| StepCox[both]+RSF | 0.500909753 | 0.521894498 | 0.505681818 | 0.524797115 | 0.502328535 | 0.499314571 |
| StepCox[backward]+RSF | 0.503423978 | 0.521894498 | 0.483522727 | 0.524797115 | 0.5 | 0.499314571 |
| StepCox[both]+SuperPC | 0.58326717 | 0.57424844 | 0.643181818 | 0.533814247 | 0.554614733 | 0.552853442 |
| StepCox[backward]+SuperPC | 0.58326717 | 0.57424844 | 0.643181818 | 0.533814247 | 0.554614733 | 0.552853442 |
