# Supplementary material for: Robust machine−learning based prognostic index using cytotoxic T lymphocyte evasion genes highlights potential therapeutic targets in colorectal cancer
Source: Cancer Cell Int. 2024 Jan 31;24:52. doi: 10.1186/s12935-024-03239-y (PMC10829178; doi:10.1186/s12935-024-03239-y)
Supplement: Supplementary file 1 — Additional file 1: Figure S1. (A) Expression and (B) prognostic significance of 31 core CERGs in TCGA-CRC dataset. Figure S2. IHC score of HOXC6 (A), G0S2 (B), and MX2 (C) in normal tissues and CRC. **p < 0.01; ***p < 0.001. Table S1. Published signatures applied for model comparison. Table S2. Sequences for qRT-PCR primers. Table S3. Detailed si-RNA sequences used in the study. Table S4. 182 CERGs from published research and 1793 IRGs from Immport database. Table S5. Published signatures applied for model comparison. C-index of each combination of machine learning method for developing the prognostic signature. Table S6. AUC value of each combination of machine learning method for constructing the immunotherapy-related signature. [file 12935_2024_3239_MOESM1_ESM.zip › Supplementary Material/Supplementary Table S6.docx]

**Supplementary Table S6: AUC value of each combination of machine learning method for constructing the immunotherapy-related signature.**

| iMvigor210 | GSE35640 | GSE78220 | GSE91061 | GSE100797 | GSE179351 | PRJEB25780 |
| --- | --- | --- | --- | --- | --- | --- |
| SVM | 0.588235294 | 0.5 | 0.5 | 0.5 | 0.5 | 0.5 |
| glmBoost+SVM | 0.529411765 | 0.5 | 0.5 | 0.5 | 0.5 | 0.5 |
| Ridge | 0.753580563 | 0.530748663 | 0.627777778 | 0.59650053 | 0.573333333 | 0.575757576 |
| Lasso+SVM | 0.5 | 0.5 | 0.5 | 0.5 | 0.5 | 0.5 |
| glmBoost+Ridge | 0.735613811 | 0.506684492 | 0.566666667 | 0.473488865 | 0.54 | 0.562770563 |
| Enet[alpha=0.1] | 0.75415601 | 0.495989305 | 0.583333333 | 0.567338282 | 0.56 | 0.554112554 |
| glmBoost+Enet[alpha=0.1] | 0.736892583 | 0.518716578 | 0.561111111 | 0.469777306 | 0.513333333 | 0.567099567 |
| Enet[alpha=0.2] | 0.746035806 | 0.522727273 | 0.588888889 | 0.54718982 | 0.473333333 | 0.554112554 |
| Enet[alpha=0.3] | 0.73286445 | 0.532085561 | 0.6 | 0.539766702 | 0.453333333 | 0.554112554 |
| glmBoost+Enet[alpha=0.3] | 0.73887468 | 0.526737968 | 0.555555556 | 0.468186638 | 0.493333333 | 0.441558442 |
| glmBoost+Enet[alpha=0.2] | 0.738171355 | 0.521390374 | 0.555555556 | 0.468186638 | 0.493333333 | 0.567099567 |
| Enet[alpha=0.4] | 0.730370844 | 0.545454545 | 0.594444444 | 0.543478261 | 0.46 | 0.558441558 |
| glmBoost+Enet[alpha=0.4] | 0.738363171 | 0.528074866 | 0.55 | 0.467656416 | 0.5 | 0.445887446 |
| Lasso+glmBoost | 0.738299233 | 0.537433155 | 0.538888889 | 0.53340403 | 0.493333333 | 0.445887446 |
| Enet[alpha=0.5] | 0.724424552 | 0.550802139 | 0.594444444 | 0.544008484 | 0.46 | 0.558441558 |
| glmBoost | 0.726150895 | 0.560160428 | 0.583333333 | 0.545068929 | 0.473333333 | 0.545454545 |
| glmBoost+Enet[alpha=0.5] | 0.738810742 | 0.530748663 | 0.544444444 | 0.469777306 | 0.506666667 | 0.441558442 |
| Enet[alpha=0.6] | 0.723913043 | 0.556149733 | 0.594444444 | 0.544538706 | 0.466666667 | 0.554112554 |
| glmBoost+Enet[alpha=0.6] | 0.738235294 | 0.532085561 | 0.55 | 0.533934252 | 0.506666667 | 0.441558442 |
| glmBoost+Enet[alpha=0.7] | 0.737595908 | 0.532085561 | 0.55 | 0.534464475 | 0.506666667 | 0.445887446 |
| glmBoost+Enet[alpha=0.8] | 0.738618926 | 0.530748663 | 0.538888889 | 0.53340403 | 0.506666667 | 0.441558442 |
| Enet[alpha=0.8] | 0.730946292 | 0.556149733 | 0.577777778 | 0.537645811 | 0.493333333 | 0.536796537 |
| Enet[alpha=0.9] | 0.728644501 | 0.564171123 | 0.577777778 | 0.540827147 | 0.48 | 0.54978355 |
| Lasso | 0.728069054 | 0.564171123 | 0.577777778 | 0.539766702 | 0.48 | 0.545454545 |
| Enet[alpha=0.7] | 0.718861893 | 0.561497326 | 0.588888889 | 0.547720042 | 0.466666667 | 0.536796537 |
| glmBoost+Enet[alpha=0.9] | 0.738682864 | 0.530748663 | 0.538888889 | 0.534464475 | 0.506666667 | 0.441558442 |
| glmBoost+Lasso | 0.738363171 | 0.526737968 | 0.533333333 | 0.465005302 | 0.506666667 | 0.445887446 |
| glmBoost+Stepglm[forward] | 0.73842711 | 0.52540107 | 0.533333333 | 0.46659597 | 0.513333333 | 0.445887446 |
| Lasso+Stepglm[forward] | 0.73842711 | 0.52540107 | 0.533333333 | 0.46659597 | 0.513333333 | 0.445887446 |
| Stepglm[forward] | 0.774296675 | 0.524064171 | 0.588888889 | 0.591728526 | 0.52 | 0.458874459 |
| plsRglm | 0.738171355 | 0.549465241 | 0.583333333 | 0.525450689 | 0.56 | 0.567099567 |
| Stepglm[both]+Ridge | 0.751342711 | 0.528074866 | 0.627777778 | 0.567338282 | 0.426666667 | 0.45021645 |
| Stepglm[backward]+Ridge | 0.752237852 | 0.528074866 | 0.627777778 | 0.571580064 | 0.44 | 0.558441558 |
| Stepglm[both]+plsRglm | 0.727365729 | 0.489304813 | 0.627777778 | 0.525980912 | 0.513333333 | 0.545454545 |
| Stepglm[backward]+plsRglm | 0.727365729 | 0.489304813 | 0.627777778 | 0.525980912 | 0.513333333 | 0.545454545 |
| Stepglm[both]+Enet[alpha=0.9] | 0.754987212 | 0.532085561 | 0.622222222 | 0.575821845 | 0.44 | 0.567099567 |
| Stepglm[backward]+Enet[alpha=0.9] | 0.755626598 | 0.522727273 | 0.616666667 | 0.577412513 | 0.446666667 | 0.54978355 |
| Stepglm[both]+Enet[alpha=0.1] | 0.753005115 | 0.528074866 | 0.627777778 | 0.571580064 | 0.446666667 | 0.562770563 |
| Stepglm[backward]+Enet[alpha=0.1] | 0.753132992 | 0.526737968 | 0.627777778 | 0.572640509 | 0.446666667 | 0.562770563 |
| Stepglm[both]+Enet[alpha=0.8] | 0.754795396 | 0.528074866 | 0.622222222 | 0.576352068 | 0.44 | 0.558441558 |
| Stepglm[backward]+Enet[alpha=0.8] | 0.754795396 | 0.528074866 | 0.622222222 | 0.576352068 | 0.44 | 0.558441558 |
| Stepglm[both]+Enet[alpha=0.2] | 0.752109974 | 0.532085561 | 0.622222222 | 0.571049841 | 0.426666667 | 0.45021645 |
| Stepglm[backward]+Enet[alpha=0.2] | 0.754539642 | 0.526737968 | 0.622222222 | 0.5747614 | 0.44 | 0.567099567 |
| Stepglm[both]+Lasso | 0.754539642 | 0.529411765 | 0.622222222 | 0.576882291 | 0.44 | 0.562770563 |
| Stepglm[backward]+Lasso | 0.754603581 | 0.526737968 | 0.616666667 | 0.576352068 | 0.44 | 0.558441558 |
| Stepglm[both]+Enet[alpha=0.6] | 0.755626598 | 0.522727273 | 0.616666667 | 0.577942736 | 0.44 | 0.54978355 |
| Stepglm[backward]+Enet[alpha=0.6] | 0.754859335 | 0.526737968 | 0.622222222 | 0.576352068 | 0.44 | 0.558441558 |
| glmBoost+GBM | 0.868925831 | 0.510695187 | 0.455555556 | 0.53340403 | 0.586666667 | 0.463203463 |
| Stepglm[both]+Enet[alpha=0.7] | 0.75511509 | 0.524064171 | 0.616666667 | 0.576352068 | 0.44 | 0.54978355 |
| Stepglm[backward]+Enet[alpha=0.7] | 0.75556266 | 0.522727273 | 0.616666667 | 0.577412513 | 0.446666667 | 0.554112554 |
| Stepglm[both] | 0.75415601 | 0.520053476 | 0.627777778 | 0.583244963 | 0.466666667 | 0.558441558 |
| Stepglm[backward] | 0.75415601 | 0.520053476 | 0.627777778 | 0.583244963 | 0.466666667 | 0.558441558 |
| Stepglm[both]+Enet[alpha=0.4] | 0.753644501 | 0.537433155 | 0.622222222 | 0.572640509 | 0.433333333 | 0.45021645 |
| Stepglm[backward]+Enet[alpha=0.4] | 0.754731458 | 0.528074866 | 0.622222222 | 0.575821845 | 0.44 | 0.567099567 |
| Stepglm[both]+Enet[alpha=0.3] | 0.754219949 | 0.53342246 | 0.622222222 | 0.575291622 | 0.446666667 | 0.558441558 |
| Stepglm[backward]+Enet[alpha=0.3] | 0.754219949 | 0.524064171 | 0.616666667 | 0.575821845 | 0.44 | 0.562770563 |
| Stepglm[both]+glmBoost | 0.742199488 | 0.554812834 | 0.566666667 | 0.546659597 | 0.486666667 | 0.484848485 |
| Stepglm[backward]+glmBoost | 0.742199488 | 0.554812834 | 0.566666667 | 0.546659597 | 0.486666667 | 0.484848485 |
| Stepglm[both]+Enet[alpha=0.5] | 0.754923274 | 0.529411765 | 0.622222222 | 0.576352068 | 0.446666667 | 0.567099567 |
| Stepglm[backward]+Enet[alpha=0.5] | 0.754667519 | 0.522727273 | 0.616666667 | 0.576352068 | 0.44 | 0.554112554 |
| glmBoost+RF | 0.997953964 | 0.516042781 | 0.544444444 | 0.53552492 | 0.546666667 | 0.441558442 |
| Lasso+GBM | 0.865025575 | 0.50802139 | 0.477777778 | 0.540827147 | 0.613333333 | 0.558441558 |
| GBM | 0.9342711 | 0.522727273 | 0.533333333 | 0.483563097 | 0.573333333 | 0.515151515 |
| Stepglm[both]+SVM | 0.514705882 | 0.5 | 0.5 | 0.5 | 0.5 | 0.5 |
| Stepglm[backward]+SVM | 0.522058824 | 0.5 | 0.5 | 0.5 | 0.5 | 0.5 |
| Lasso+RF | 0.998529412 | 0.524064171 | 0.538888889 | 0.552492047 | 0.52 | 0.432900433 |
| Stepglm[both]+GBM | 0.891368286 | 0.529411765 | 0.5 | 0.556203606 | 0.66 | 0.562770563 |
| Stepglm[backward]+GBM | 0.902493606 | 0.532085561 | 0.488888889 | 0.563096501 | 0.686666667 | 0.523809524 |
| Stepglm[both]+RF | 0.999936061 | 0.445187166 | 0.444444444 | 0.566808059 | 0.646666667 | 0.502164502 |
| LDA | 0.773657289 | 0.512032086 | 0.572222222 | 0.589077413 | 0.566666667 | 0.437229437 |
| glmBoost+LDA | 0.74028133 | 0.53342246 | 0.533333333 | 0.530222694 | 0.52 | 0.445887446 |
| Stepglm[both]+LDA | 0.755242967 | 0.538770053 | 0.605555556 | 0.584305408 | 0.426666667 | 0.571428571 |
| Stepglm[backward]+LDA | 0.755242967 | 0.538770053 | 0.605555556 | 0.584305408 | 0.426666667 | 0.571428571 |
| Lasso+LDA | 0.74028133 | 0.53342246 | 0.533333333 | 0.530222694 | 0.52 | 0.445887446 |
| Stepglm[backward]+RF | 0.999936061 | 0.554812834 | 0.427777778 | 0.580063627 | 0.373333333 | 0.545454545 |
| XGBoost | 0.70834399 | 0.518048128 | 0.558333333 | 0.526776246 | 0.363333333 | 0.571428571 |
| Lasso+XGBoost | 0.795588235 | 0.50802139 | 0.555555556 | 0.448038176 | 0.6 | 0.61038961 |
| glmBoost+XGBoost | 0.702749361 | 0.532085561 | 0.583333333 | 0.513520679 | 0.633333333 | 0.547619048 |
| Stepglm[both]+XGBoost | 0.795588235 | 0.50802139 | 0.555555556 | 0.448038176 | 0.6 | 0.61038961 |
| Stepglm[backward]+XGBoost | 0.702749361 | 0.532085561 | 0.583333333 | 0.513520679 | 0.633333333 | 0.547619048 |
| NaiveBayes | 0.71898977 | 0.610962567 | 0.583333333 | 0.579003181 | 0.72 | 0.636363636 |
| Lasso+NaiveBayes | 0.709462916 | 0.526737968 | 0.561111111 | 0.50795334 | 0.526666667 | 0.476190476 |
| glmBoost+NaiveBayes | 0.709462916 | 0.526737968 | 0.561111111 | 0.50795334 | 0.526666667 | 0.476190476 |
| Stepglm[both]+NaiveBayes | 0.715473146 | 0.522727273 | 0.655555556 | 0.575821845 | 0.6 | 0.493506494 |
| Stepglm[backward]+NaiveBayes | 0.715473146 | 0.522727273 | 0.655555556 | 0.575821845 | 0.6 | 0.493506494 |
