# Supplementary figures and images for: Robust machine−learning based prognostic index using cytotoxic T lymphocyte evasion genes highlights potential therapeutic targets in colorectal cancer
Source: Cancer Cell Int. 2024 Jan 31;24:52. doi: 10.1186/s12935-024-03239-y (PMC10829178; doi:10.1186/s12935-024-03239-y)

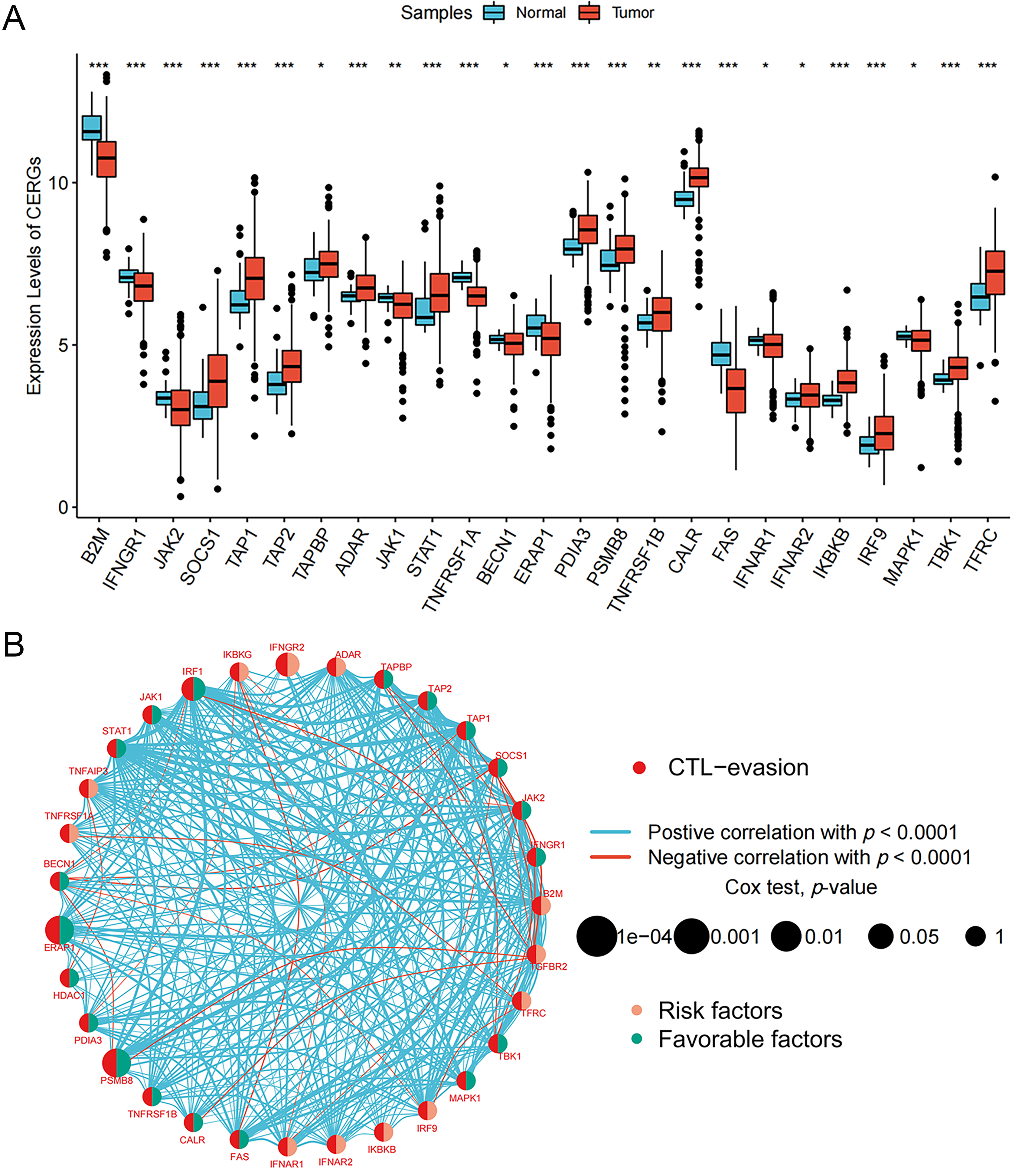

Supplement: Supplementary file 1 — Additional file 1: Figure S1. (A) Expression and (B) prognostic significance of 31 core CERGs in TCGA-CRC dataset. Figure S2. IHC score of HOXC6 (A), G0S2 (B), and MX2 (C) in normal tissues and CRC. **p < 0.01; ***p < 0.001. Table S1. Published signatures applied for model comparison. Table S2. Sequences for qRT-PCR primers. Table S3. Detailed si-RNA sequences used in the study. Table S4. 182 CERGs from published research and 1793 IRGs from Immport database. Table S5. Published signatures applied for model comparison. C-index of each combination of machine learning method for developing the prognostic signature. Table S6. AUC value of each combination of machine learning method for constructing the immunotherapy-related signature. [file 12935_2024_3239_MOESM1_ESM.zip › Supplementary Material/Supplementary Figure S1.tif]

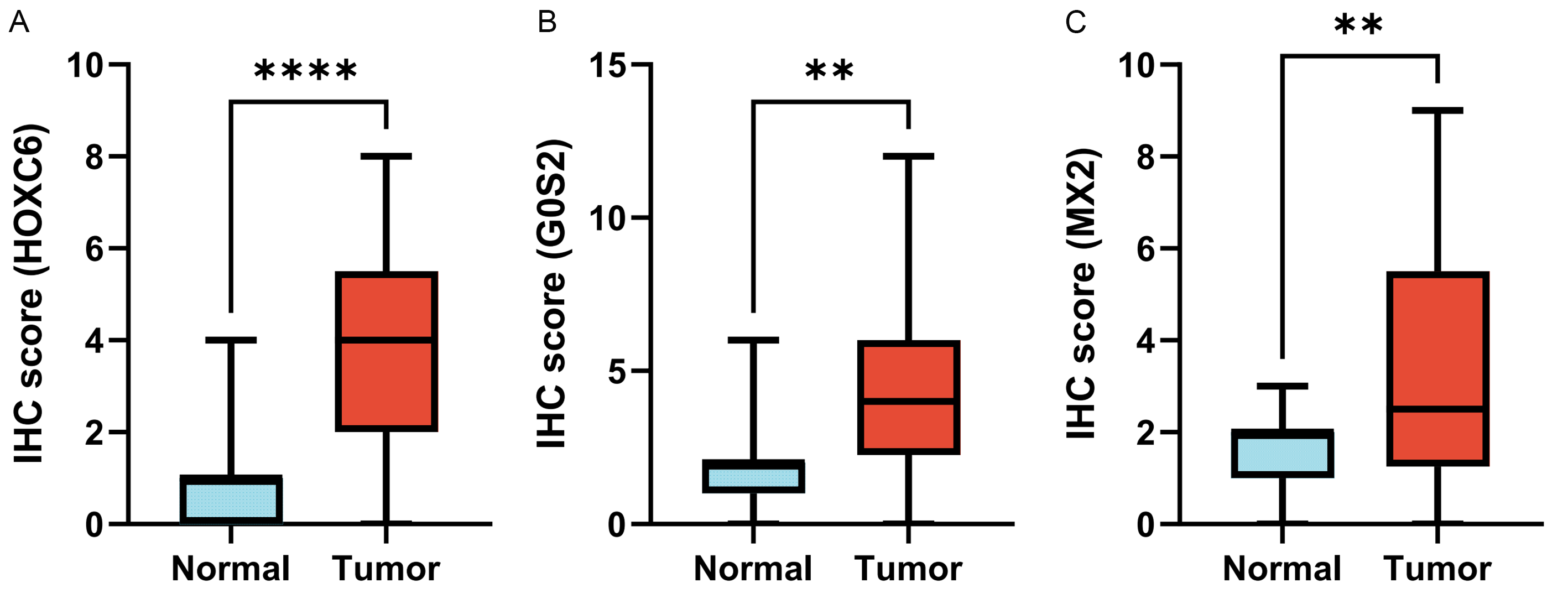

Supplement: Supplementary file 1 — Additional file 1: Figure S1. (A) Expression and (B) prognostic significance of 31 core CERGs in TCGA-CRC dataset. Figure S2. IHC score of HOXC6 (A), G0S2 (B), and MX2 (C) in normal tissues and CRC. **p < 0.01; ***p < 0.001. Table S1. Published signatures applied for model comparison. Table S2. Sequences for qRT-PCR primers. Table S3. Detailed si-RNA sequences used in the study. Table S4. 182 CERGs from published research and 1793 IRGs from Immport database. Table S5. Published signatures applied for model comparison. C-index of each combination of machine learning method for developing the prognostic signature. Table S6. AUC value of each combination of machine learning method for constructing the immunotherapy-related signature. [file 12935_2024_3239_MOESM1_ESM.zip › Supplementary Material/Supplementary Figure S2.tif]
